# Land cover as a driver of fish community changes in New York’s Oswego River Watershed

**DOI:** 10.1371/journal.pone.0327293

**Published:** 2025-07-14

**Authors:** Kate M. Henderson, Megan Hazlett, Joshua A. Drew

**Affiliations:** Department of Environmental Biology, The State University of New York College of Environmental Science and Forestry, Syracuse, New York, United States of America; University of Nevada, Reno, UNITED STATES OF AMERICA

## Abstract

Freshwater fish communities in New York State, USA, have been impacted by a variety of threats over the last century, including changes in land cover. Land cover exerts a powerful influence on aquatic communities at multiple spatial scales, and alterations to systems can persist even after restoration actions are taken. Our research examines how land cover changes were correlated with changes in fish species richness in a nearly 100-year dataset from New York’s Oswego River Watershed. The watershed was heavily agricultural in the early 1900s and was modified by both reforestation and urbanization in the subsequent century, two changes which we may expect to have opposite effects on biodiversity. Linear mixed effects models showed that species richness correlated positively with natural and urban land cover and negatively with agricultural land cover, with increases in the richness of sediment-tolerant, temperature-tolerant, and nonnative species driving the urban increase. Understanding how historical changes in land cover have affected species richness can help inform predictions about future changes to fish communities as formerly agricultural regions experience the conflicting effects of reforestation and urbanization.

## Introduction

Land cover exerts major influences on aquatic communities through multiple pathways and at multiple spatial scales [1]; however, it can be difficult to study the effects of land cover change on fish and other aquatic species without long-term data sets for a region. Conversion from natural land cover to agriculture causes an increase in sunlight exposure and water temperatures; variable changes to hydrology; a loss of large wood in stream channels; and increased non-point-source pollution through input of fine sediments, nutrients, and pesticides [1–5]. Urban land cover has a strongly negative impact on many fish species through mechanisms such as pollution, erratic hydrology, loss of channel and habitat structure, and increased temperature [1,6,7]. Urbanization and the commensurate increase in impervious surfaces of terrestrial areas may decrease aquatic species richness through the loss of habitat specialists, species sensitive to changes in temperature or water chemistry, or species at the edge of their ranges which were already at the boundaries of their tolerances [8,9]. Both urbanization and agricultural conversion can lead to the loss and fragmentation of wetlands, resulting in the loss of important spawning habitat [10]. However, disturbance on its own does not necessarily lead to a decline in the total number of species present—in some cases deforestation and habitat degradation can lead to an increase in aquatic species richness as tolerant generalist species move into areas once dominated by endemic specialists, and at intermediate levels of disturbance both generalists and specialists can be present [11]. In these ecosystems, widespread generalists are the “winners” of disturbance and can further expand their ranges into new areas [12].

There are also important temporal components to environmental change and its impacts on biodiversity. Alterations to species composition persist even when restoration actions are taken in urban ecosystems [13], and aquatic communities may remain altered for decades after agricultural land is restored to forests – Harding et al. [14] found that land use in the 1950s was a better predictor of fish and aquatic invertebrate diversity in two North Carolina rivers than 1990s land use. Similar time-lag responses to land cover have been seen in South American fishes [15], Michigan water quality [16], and threshold responses of United States fish communities [17]. If we want to fully understand the impacts of land cover change, we must consider them at a time span of decades to centuries rather than individual years.

Typically, we would study how land cover changes affect communities by using a space-for-time exchange. For ecosystems where we lack a historical record with which to establish baselines, we sample extant, but less disturbed systems within a similar biogeographic area [18,19]. Although a space for time exchange approach includes simplifying assumptions, this methodology has been widely used to characterize previous ecological states for anthropogenically disturbed ecosystems [20]. However, there are alternatives to using these space-for-time exchanges—when possible, it can be preferable to use long-term datasets from a single site to understand what has shaped its current status rather than making assumptions about the similarity of a reference site to the historical condition of a disturbed site. Historical ecology is valuable because it allows us to understand change over time by using past data to reconstruct ecosystems [21,22]. Historical ecology approaches can reveal unexpected results that are counter to ideas based on incomplete data and/or research conducted in ecosystems that are already disturbed. As such, historical datasets can provide important insights for conservation [21]. Ecosystems with adequate historical data can provide insight into how past land use changes have affected biodiversity and help inform conservation actions aimed to reduce the impacts of any future land cover changes on fish communities. However, many systems of interest do not have sufficiently detailed historical sampling records to detect these changes. It is important to study systems where historical records exist in order to inform our understanding about both those ecosystems and nearby watersheds with less data availability.

**Fig 1:**
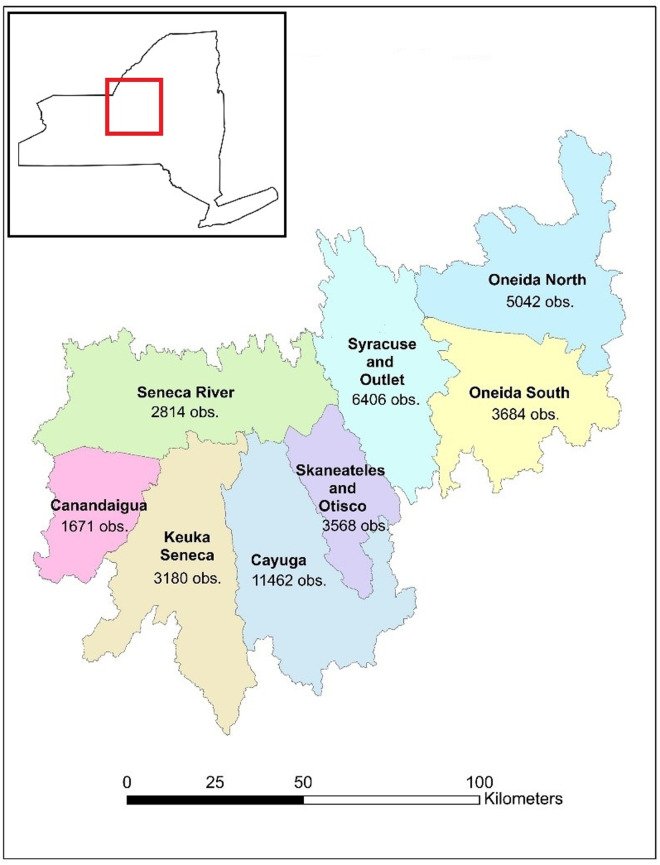
Oswego River Watershed Map. A map of the Oswego River Watershed, showing the eight sub-basins we used in this study. The number under each sub-basin name indicates the number of fish observations from that sub-basin. The location of the study area within New York State is indicated in red.

The Oswego River Watershed is an area with a rich historical data set, and which has experienced major changes in the environment. This watershed is part of the economically and ecologically important Laurentide Great Lakes drainage basin and encompasses 13,130 km^2^ of Central New York in the United States, draining an area that extends through the Finger Lakes region and Oneida Lake to Lake Ontario (Fig 1). The Oswego River Watershed has the highest fish species richness of any New York Great Lakes tributary system—110 total species present as of 2004, of which 92 were native (58.2% of New York’s native freshwater species and 62.5% of the state’s total freshwater species)— due in part to the presence of the Finger Lakes [23]. Historical data are available in New York from watershed surveys led by Emmeline Moore for the New York Conservation Department in the 1920s and 1930s [24–26]. The Oswego River Watershed was surveyed in the summer of 1927, with 100 species of fish recorded and nearly 1,300 observations entered in the New York State Department of Environmental Conservation’s (NYSDEC) records [27, 28], records available online at https://www.nysm.nysed.gov/nysm-fish-atlas-database and summarized in [29]. These historical data make the Oswego River Watershed a valuable study system because we can directly examine change over time at specific sites.

This watershed has experienced numerous anthropogenic alterations over the past century and continues to be in flux. As a result of both historical and contemporary data this study system allows us to tease apart the impacts of nonnative fish introductions and land use changes, including restoration efforts, on fish biodiversity. The watershed has experienced species introductions via the Erie Canal as early as 1825 [28,29], extirpations and range reductions of native species due to disturbances prior to the 1930s [23,30], and the introduction of more than a dozen fish species through both intentional stocking and unintentional introductions since the 1940s [23]. An additional driver of changes in fish communities is land cover change. The Oswego River Watershed, like Central New York as a whole, was originally the land of the Haudenosaunee Confederacy who managed the land through small scale, fire-based agriculture, and subsistence hunting [31]. With European colonization the land saw an increase in larger-scale infrastructure and associated deforestation and by the 1900s most of the land was heavily agricultural. In the subsequent century, large areas of agricultural land have been abandoned and allowed to revert to their original forested states [32], which may have improved suitable habitat conditions for native endemic species. However, the area has also experienced urbanization, which may negatively impact some species.

We quantified how changes in land cover potentially affect fish communities in the Oswego River Watershed over the 20^th^ and early 21^st^ centuries. We investigated whether land cover change impacted species richness in sub-basins of the watershed with different land cover histories, and also examined whether certain groups (based on tolerances and native or nonnative status) were more impacted by altered land cover than others. Understanding patterns within the watershed can improve our ability to make plans for the Great Lakes region as a whole, particularly for watersheds with less historical survey data available. We hypothesized an increased species richness with declines in agricultural land cover and increases in natural land cover and decreases in species richness associated with increased urbanization. We also hypothesized that nonnative generalist and tolerant species would be more abundant under urbanization than would native, environmentally sensitive, and specialist species due to reduced habitat complexity and degraded water conditions.

## Materials and methods

Our goal in this paper was to examine how species richness changed over time across the Oswego River Drainage as the region’s land cover shifted. To test for potential effects of land cover on species richness we used linear mixed effects models. Our land cover percentages were autocorrelated, so models were run individually using single land cover types. Broad trends obtained using nonmetric multidimensional scaling (NMDS) were similar to the ones from the individual linear mixed effects models, so we focus on the mixed effects models in this article. We initially used the full species richness data set to test the overarching effect that land cover has on the fish present, and then ran the models using subsets of the fish data (for example, only native or only nonnative species) to determine whether specific tolerance levels or native/nonnative status of fish correlated more strongly with the overall changes.

### Data sources

We obtained fish survey data from the New York Fish Atlas database, which contains observations from the New York State Department of Environmental Conservation’s surveys as well as from museum records (available online at https://www.nysm.nysed.gov/nysm-fish-atlas-database). The database contains 37,906 observations from the Oswego River Watershed (S1 Fig), spanning from 1856 to 2014. Because of the small number of observations available from the earliest years of the database, we used fish data from 1927–2014. The survey data included information such as species name, date and year collected, county, latitude, and longitude. Early fish surveys used techniques such as gill netting, seine netting, and angling [24], while electrofishing is also used in modern times. We obtained historical land cover from U.S. Geological Survey (USGS) models [33] for 1938, 1945, 1955, 1965, 1975, 1985, and 1992. The data are at a 250-m resolution with 14 land cover classes. We used modern NLCD land cover data from 2004 and 2016 [34]; because these data were originally at a 30-m resolution, we used the Resample tool in a geographic information system (GIS) software ESRI ArcGIS 10.8.1 to match the resolution of the historical land cover data. Elevation data were obtained from USGS digital elevation models in the Cornell University Geospatial Information Library [35].

### Data preparation

**Fig 2:**
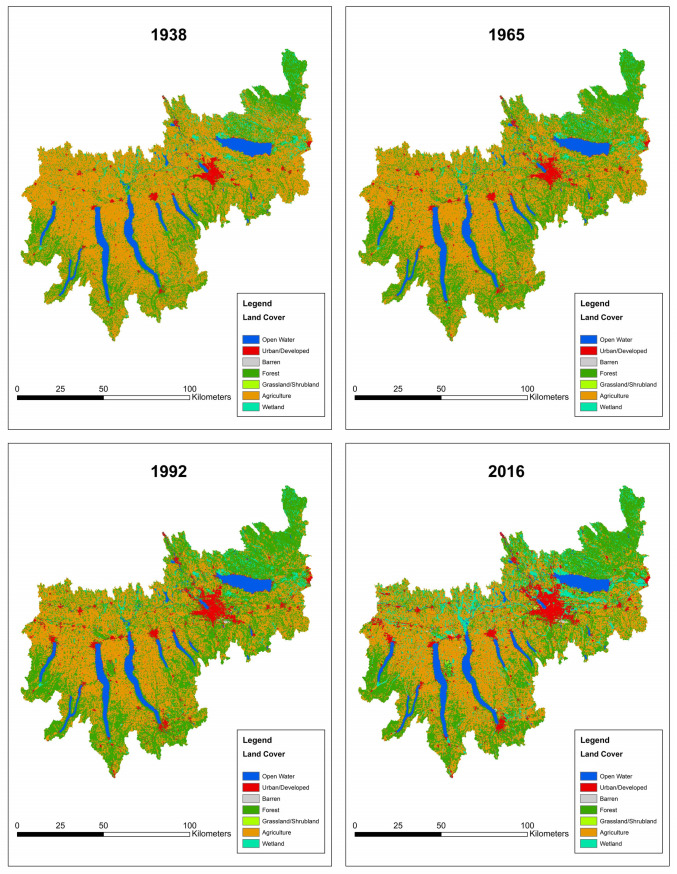
Land cover in the Oswego River Watershed in 1938 and 2016. Land cover types of focus include urban/developed (red), agricultural (orange), forested (dark green), and wetland (teal). Data are from USGS at [33,34].

We combined the land cover categories ‘Deciduous Forest’, ‘Evergreen Forest’, and ‘Mixed Forest’ into a single ‘Forest’ category, as well as ‘Herbaceous Wetland’ and ‘Woody Wetland’ into the ‘Wetland’ category. Due to discrepancies in how wetland and forest were classified in different years, and because both represent important natural habitats, these two land cover classes were also combined into a single ‘Natural’ category. ‘Pasture/Hay’ and ‘Cultivated Cropland’ were collapsed into the ‘Agriculture’ category. All intensity levels of developed land were collapsed into a single ‘Urban/Developed’ category. The three land cover types of focus throughout the study are summarized in Table 1, and land cover for 1938 (the first year available), 1965, 1992, and 2016 (the last year of the study) are shown in Fig 2 (data from [33,34]). Two periods of fish data were also combined—due to a lack of land cover data from the 1920s and fish survey data from the 1930s, fish data from the 1927 watershed survey were combined with 1930s land cover for analysis. This was the only combined time unit—all other decades had fish survey and land cover data from that decade only.

**Table 1:** Land cover in the Oswego River Watershed.

	Urban	Agricultural	Natural
**1930**	2.19%	60.27%	31.59%
**1940**	2.33%	56.89%	34.76%
**1950**	2.69%	53.35%	37.87%
**1960**	3.29%	48.4%	42.18%
**1970**	3.98%	45.07%	44.78%
**1980**	4.38%	44.37%	45.05%
**1990**	4.9%	42.22%	46.62%
**2000**	6.28%	41.25%	44.88%
**2010**	6.72%	40.77%	44.95%

A summary table of the percent coverage of the three land cover types of focus in the Oswego River Watershed over time

**Fig 3:**
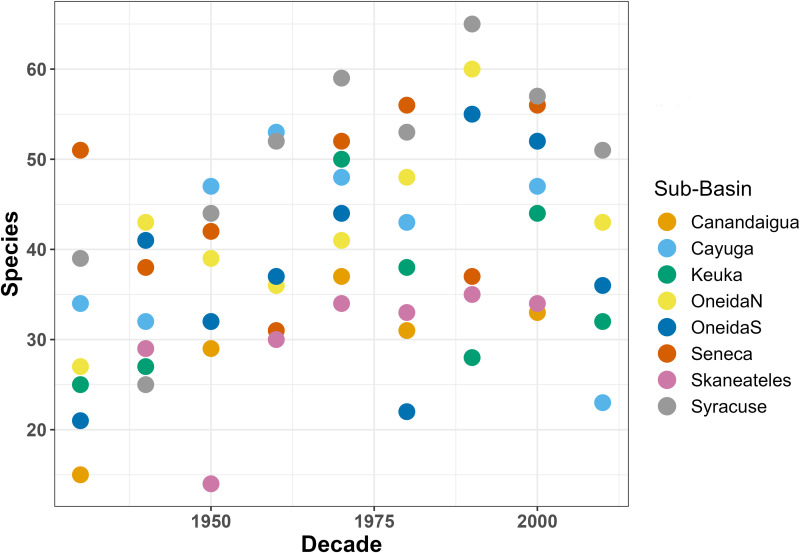
Species richness in each sub-basin of the Oswego River Watershed over time. This scatterplot shows species richness in the Oswego River Watershed over the study period, with color coding by sub-basin. Only combinations of sub-basin and decade with at least 50 observations are included.

We delineated the Oswego River Watershed using StreamStats v4.4.0 from USGS and divided the watershed into eight sub-basins, accounting for both hydrology and data availability. The sub-basins were 1) Canandaigua Lake and the surrounding areas in the southwestern portion of the watershed, 2) the Cayuga Lake drainage basin, 3) the Keuka and Seneca Lakes drainage basins, 4) the northern half of the Oneida Lake drainage basin, 5) the southern half of the Oneida Lake drainage basin, 6) the Seneca River drainage basin, 7) the Skaneateles and Otisco Lakes drainage basins, and 8) the area that drains Syracuse and the outlet to Lake Ontario (Fig 1). To investigate the potential impact of watershed versus riparian zone land cover on species richness, we calculated the percentage of land cover for both the full sub-basins and for a subset of the data containing a 100-meter “riparian” buffer around water bodies. The characteristics of each sub-basin are described in Table 2, and fish species richness in each sub-basin over time is shown in Fig 3.

**Table 2:** Characteristics of each sub-basin in the study.

Sub-Basin	Minimum Species Richness	Maximum Species Richness	Minimum Urban %	Maximum Urban %	Minimum Agricultural %	Maximum Agricultural %	Minimum Natural %	Maximum Natural %
Canandaigua	15 (1930)	37 (1960, 1970)	0.87 (1930)	3.36 (2000)	41.3 (2000)	58.1 (1930)	36.1 (1930)	49.7 (2000)
Cayuga	23 (2010)	55 (1990)	1.02 (1930)	3.81 (2010)	49.1 (1990)	64.7 (1930)	25.7 (1930)	39.1 (1990)
Keuka-Seneca	25 (1930)	50 (1970)	1.29 (1930)	4.54 (2010)	44.2 (2010)	59.4 (1930)	28.4 (1930)	41.4 (1990)
Oneida (North)	27 (1930)	60 (1990)	0.86 (1930)	3.00 (2010)	13.2 (2010)	35.8 (1930)	57.5 (1930)	75.8 (2010)
Oneida (South)	21 (1930)	55 (1990)	2.19 (1930)	8.74 (2010)	37.1 (2010)	61.0 (1930)	29.7 (1930)	47.2 (1990)
Seneca River	31 (1960)	56 (1980, 2000)	1.51 (1930)	5.09 (2000)	56.6 (2000)	79.0 (1930)	18.9 (1930)	36.3 (2000)
Skaneateles-Otisco	14 (1950)	35 (1990)	2.55 (1940)	5.07 (2000)	47.0 (1990)	55.9 (1940)	34.2 (1940)	41.8 (1970)
Syracuse	25 (1940)	65 (1990)	7.21 (1930)	18.2 (2010)	32.7 (2010)	61.2 (1930)	29.1 (1930)	46.7 (1990)

Minimum and maximum values for species richness, percent urban land cover, percent agricultural land cover, and percent natural land cover for each sub-basin. The decade where each value was observed is shown in parenthesis.

In addition to examining overall species richness, we also examined richness of groups of fish with traits that may be more or less vulnerable to land cover change—temperature tolerance, native or nonnative status, and sediment tolerance. Species with a critical temperature threshold greater than 31 °Celsius (the mean value for all 116 species ever observed in the watershed, summarized by [36]) were classified as tolerant of high temperatures, while species with critical temperature thresholds of 31 °C or lower were considered less temperature-tolerant. Species were classified as native or nonnative based on the *Atlas of the Inland Fishes of New York* [27] and the USGS Nonindigenous Aquatic Species List [37]. Finally, 68 species were classified as sediment-tolerant or sediment-intolerant based on [38] and [39], while the 48 species not mentioned in the literature were excluded from the sediment-tolerance analysis. Species classifications are summarized in S1 Table.

### Analysis

We used a paired t-test to determine whether land cover differed between the full land cover datasets and the buffer land cover datasets. For our fish dataset, we summarized the species richness for each sub-basin in each decade. We excluded site-decade combinations with fewer than 50 fish observations for lack of data (S2 Table). This filtering left us with 67 unique site-decade combinations. Because of uneven data availability between sites and decades, we also ran a more conservative, rarefied model using only site-decade combinations with at least 250 observations and using only a random selection of 250 fish observations to represent each site-decade combination that were pulled from the original observation list when more than 250 were originally recorded. These models generated similar trends to our 50 + observation models (S3 Table); therefore, we report the more inclusive 50 + observation models for the rest of this paper.

We analyzed the effect of land cover on species richness using linear mixed effects models from the package *lme4* in R version 3.6.2 [40]. Percent agricultural land cover, percent natural land cover, or percent urban land cover was used as the fixed effect for each model, while both sub-basin and decade were used as random effects. Due to the correlations between percent land cover types, each was analyzed in a separate model (Pearson correlation coefficients of 0.80 for natural and urban, −0.91 for agricultural and urban, and −0.98 for natural and agricultural). The models were compared to a null model with only random effects using ANOVA in order to determine whether significantly more variance was explained by including a fixed-effects variable at α = 0.05. A Shapiro-Wilk test was used to assure that the species richness data met the assumption of normality needed for the linear mixed effects models.

In addition to the linear mixed effects models created using full species richness, we re-ran the models using the species richness of each of six subsets of the fish assemblage: native species, nonnative species, sediment-tolerant species, sediment-intolerant species, high-temperature-tolerant species, and high-temperature-intolerant species. These results were examined to determine whether specific subgroups of fish with specific characteristics controlled the broader trends in the full richness models.

### Land cover data

Percent land cover differed among the full sub-basins and the 100-m riparian buffer areas (Table 3). Agricultural land cover was approximately 3.5% greater in the full sub-basins than the riparian zone, representing 48% of land cover as opposed to 44.5%, while natural land cover was nearly 6% greater in the 100-m buffers (both significant at p < 0.001). Anthropogenic impact via agriculture and urbanization was slightly stronger in the watershed as a whole than in the riparian zone, which may be important if the two scales differ in their influence on aquatic systems. However, models using total land cover and buffer land cover produced the same general trends, and as such, we report full land cover for the rest of this article.

**Table 3:** Paired t-test comparisons of land cover between full and buffer datasets.

	Full	100-m Buffer	Test Statistic	p-value
**Urban**	4.08%	4.06%	0.752	0.47
**Agricultural**	48.07%	44.49%	8.498	**<0.001**
**Natural**	41.41%	47.30%	−13.909	**<0.001**

Comparison of percentage urban, agricultural, and natural land cover between the full land cover datasets and the buffer land cover datasets for the watershed in each decade, with α = 0.05 (df = 8).

## Results

### Full species richness

Species richness ranged from 14 (Skaneateles, 1950s) to 65 (Syracuse, 1990s) species present in different combinations of sub-basin and decade. These data were normally distributed (Shapiro–Wilk test, W = 0.98, p = 0.54), meeting the requirements for a linear mixed effects model. The model results for full land cover are summarized in Table 4, with positive effects observed between percent urban and natural land cover and fish species richness, and a negative correlation for the agricultural model. Sub-basin explained a large portion of variance (31.32–106.11, S4 Table)—more than 50% for some models—while decade explained only a small amount (5.00–20.12, S4 Table). The distribution of the residuals met the assumptions for a linear mixed effects model for all models (S2 Fig).

**Table 4:** Summary of the linear mixed effects models using full species richness.

	Fixed effect	Fixed effect standard error	Fixed effect intercept	Fixed effect intercept standard error	ANOVA p-value
**Urban**	1.51	0.52	32.93	3.39	0.0056
**Agricultural**	−0.58	0.15	66.75	8.05	0.0071
**Natural**	0.74	0.19	8.35	8.64	0.022

A table showing the fixed effect, standard error of the fixed effect, fixed effect intercept, standard error of the fixed effect intercept, and ANOVA p-value (α = 0.05) for the models for each land cover type.

**Fig 4:**
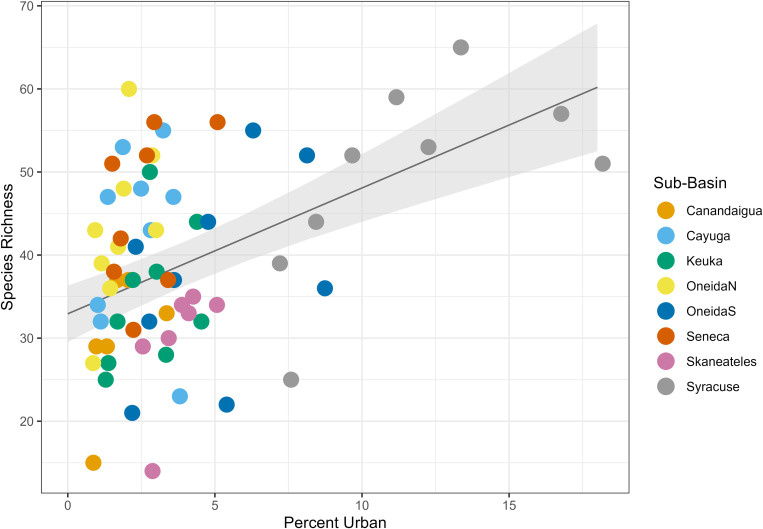
Urban land cover linear mixed effects model. Plot of the linear mixed effects model results for the effect of percent urban land cover on species richness. Gray shading shows standard error, and color coding on the data points indicates sub-basin.

**Fig 5:**
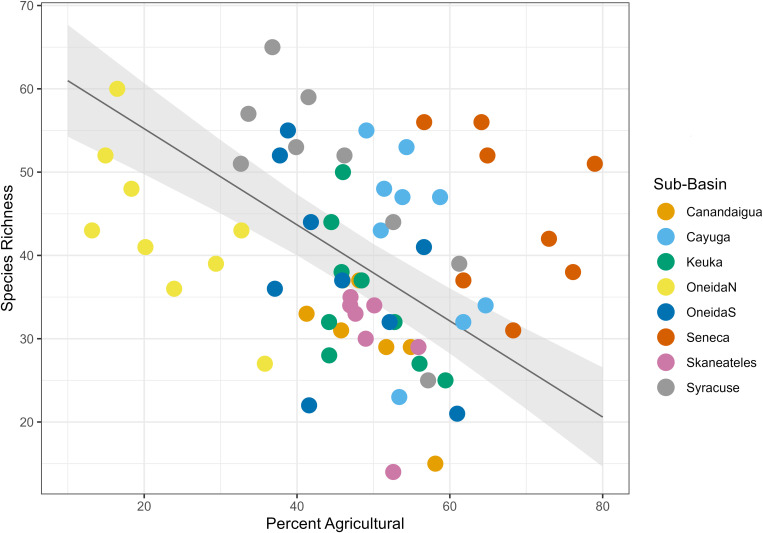
Agricultural land cover linear mixed effects model. Plot of the linear mixed effects model results for the effect of percent agricultural land cover on species richness. Gray shading shows standard error, and color coding on the data points indicates sub-basin.

**Fig 6:**
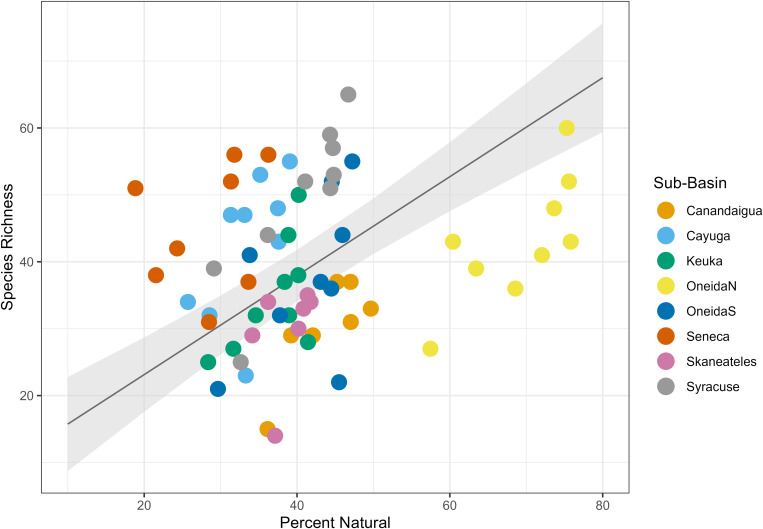
Natural land cover linear mixed effects model. Plot of the linear mixed effects model results for the effect of percent natural land cover on species richness. Gray shading shows standard error, and color coding on the data points indicates sub-basin.

We found that land type had a variable relationship with species richness. Percent urban land cover had a positive relationship with species richness (Table 4, Fig 4), with a fixed effect coefficient of 1.51 and a standard error of 0.52, such that for each 1% increase in urban land cover in the dataset, species richness increased by an average of 1.51 species. The linear mixed effects model explained significantly more variance than the null model (ANOVA, p = 0.0056). Percent agricultural land cover had a negative relationship with species richness (Table 4, Fig 5), with a fixed effect coefficient of −0.58 and a standard error of 0.15, meaning an average loss of 0.58 species for each additional percentage of agricultural land. The linear mixed effects model explained significantly more variance than the null model (ANOVA, p = 0.0071). Finally, percent natural land cover had a positive relationship with species richness (Table 4, Fig 6) with a fixed effect coefficient of 0.74 and a standard error of 0.19, meaning a gain of 0.74 species for each additional percentage of natural land. This model also explained significantly more variance than the null model l (ANOVA, p = 0.022).

### Patterns across species

**Fig 7:**
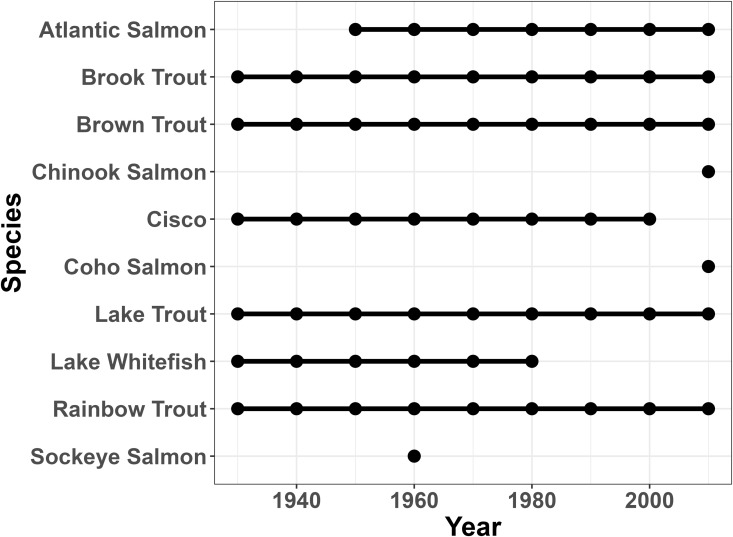
Salmonidae presence in the watershed over time. Presence timeline for members of the family Salmonidae over time in the entire Oswego River Watershed.

**Fig 8:**
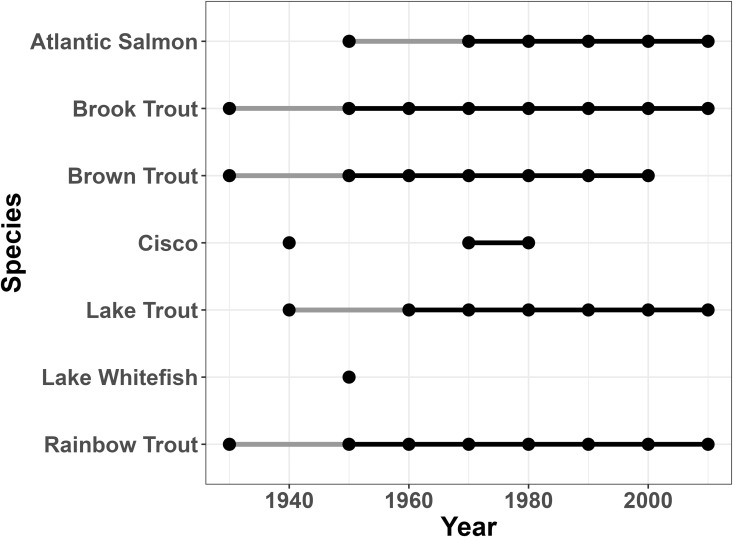
Salmonidae presence in the Cayuga sub-basin over time. Presence timeline for members of the family Salmonidae over time in the Cayuga sub-basin.

**Fig 9:**
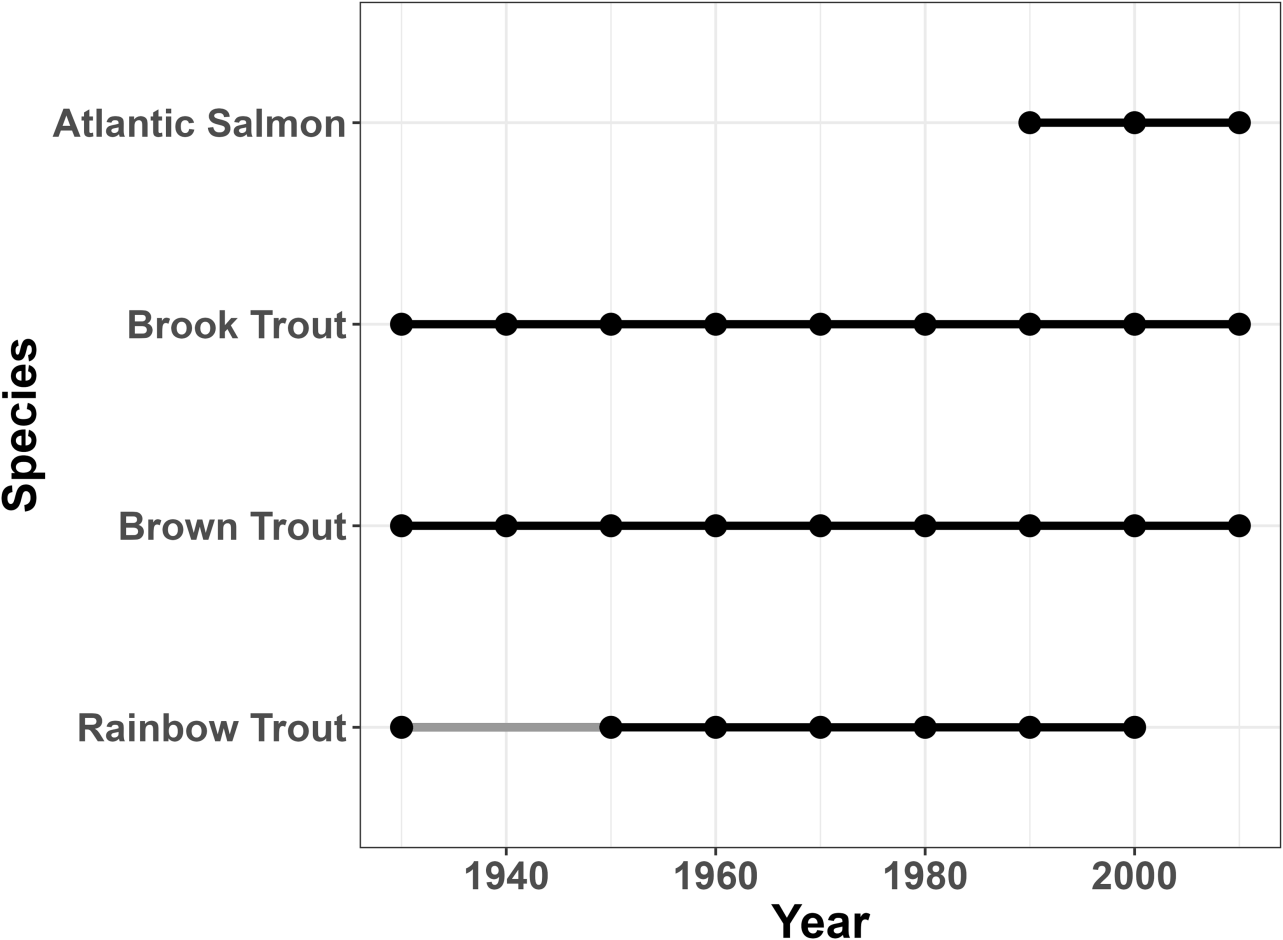
Salmonidae presence in the Oneida North sub-basin over time. Presence timeline for members of the family Salmonidae over time in the Oneida North sub-basin.

**Fig 10:**
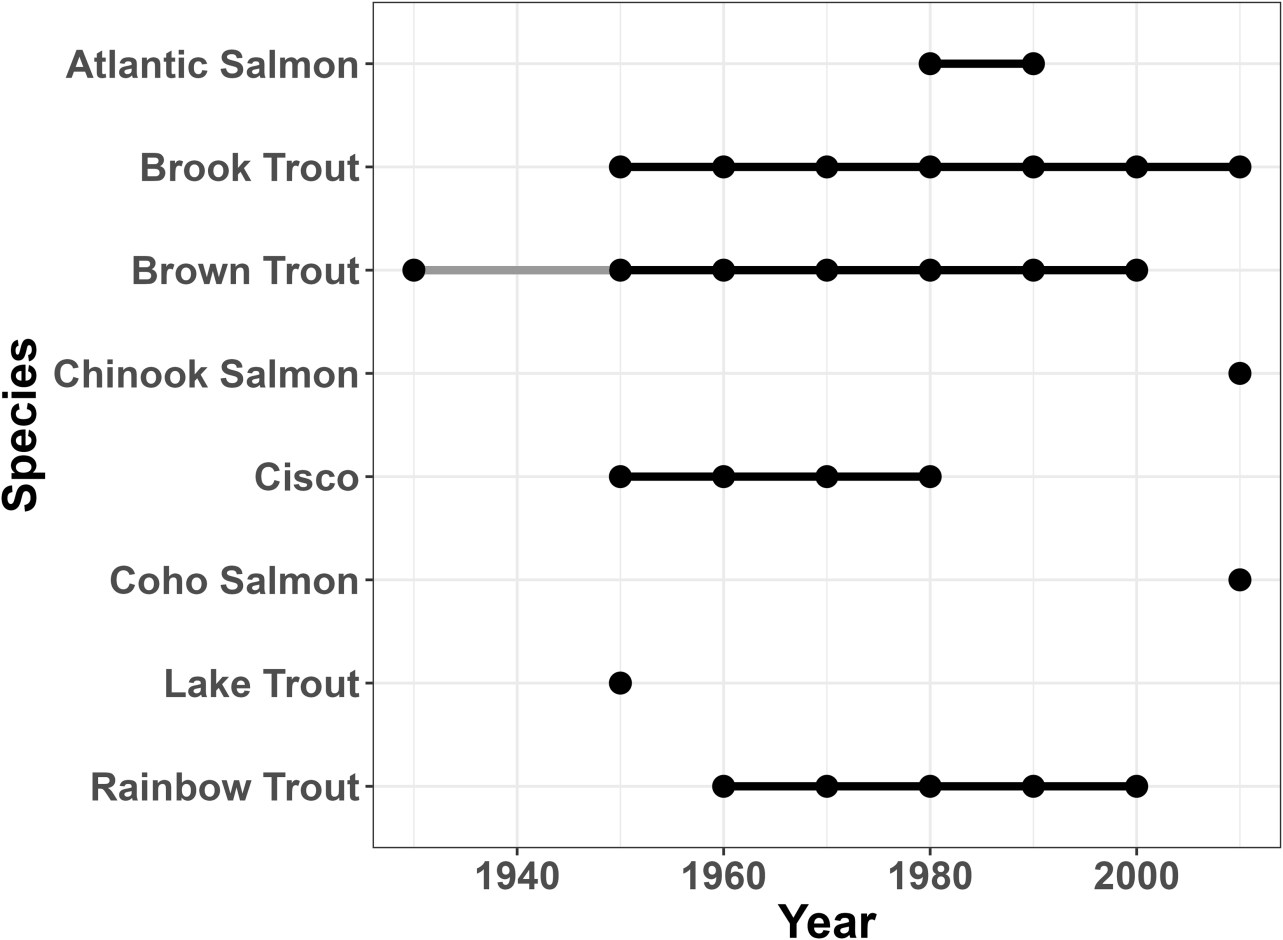
Salmonidae presence in the Syracuse sub-basin over time. Presence timeline for members of the family Salmonidae over time in the Syracuse sub-basin.

In addition to examining general trends in the changes in species richness across the watershed, we can also examine which species and subgroupings of species drive these patterns. Table 5 shows species gains and losses in the watershed across the study period, and tables for each subunit are available in S5 Table. S6 Table tracks each individual species over time. In the early years of the study, gains mainly represent historically-present species being detected in the study for the first time, while later gains represent reintroductions of rare or extirpated species (Atlantic Salmon, *Salmo salar* first appearing in the table in the 1950s) or arrivals of nonnative species such as Common Rudd (*Scardinius erythrophthalmus*) in the 1990s and Round Goby (*Neogobius melanostomus*) in the 2000s (S6 Table). Overall, we see a steady core of species present in the Oswego River Watershed as a whole, with some fluctuation that could be based on sampling effort (missing and rejoining species which only avoided detection for a single sampling period), with other longer-term changes of species joining the assemblage for the first time or dropping out of the assemblage for two or more decades. This stability gives us confidence that species detection was good even as sampling methodology changed over the course of nine decades of field sampling. More variability was present in each sub-basin (S5 and S6 Tables), which could be driven partially by the shifting land cover patterns within sub-basins. Fig 7 shows Salmonidae presence in the entire Oswego River Watershed, and Figs 8–10 show Salmonidae presence in three sub-basins as an example—since Salmonidae are a group of large game fish and one of the focal groups of the original 1927 watershed survey, we are confident that sampling effort was high across time. Additional timelines for the family Centrarchidae are available in S3 Fig.

**Table 5:** Species gains and losses over time in the Oswego River Watershed.

	1930	1940	1950	1960	1970	1980	1990	2000	2010
Add (First Time)	80	10	7	3	2	1	3	3	2
Reappear (Had been absent 1 period)	NA	NA	6	3	5	3	6	3	1
Rejoin (Had been absent 2 + periods)	NA	NA	NA	1	5	2	3	2	1
Remained	NA	68	67	73	71	72	73	79	73
Missing (absent 1 period)	NA	12	11	7	9	11	5	6	14
Lost (absent 2 periods)	NA	NA	6	8	2	6	5	2	5
**Total Richness**	80	78	80	80	83	78	85	87	77

A table showing species added (appeared in the watershed for the first time), reappeared (present after having been absent for the previous decade), rejoined (present after having been absent for two or more decades), remained, missing (absent after being present in the previous decade), and lost (absent for two consecutive decades).

**Fig 11:**
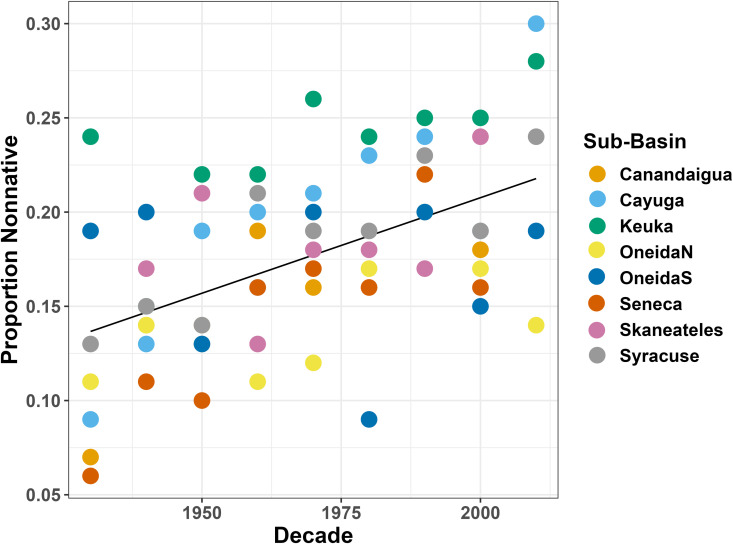
Proportion of nonnative species in the watershed over time. A plot showing the proportion of nonnative species over time, by watershed subunit.

We also see a significant increase in the proportion of nonnative species in the watershed over time (ANOVA comparison of linear mixed effects model of nonnative species proportion by decade against a null model, p < 0.001) (Fig 11).

### Species richness of fish subgroupings

In addition to examining trends in overall species richness, we investigated species richness for specific groups of species with regard to land cover changes-- native vs nonnative, tolerant vs intolerant of suspended sediments, and tolerant vs intolerant of high temperatures. All data sets were normally distributed (Shapiro-Wilk test at α = 0.05), and the number of species in each set is summarized in Table 6. Results are summarized in Table 7, with full results available in S7 Table.

**Table 6:** Number of species in different groupings.

Species Grouping	Min # of Species	Max # of Species
Native	11 (Skaneateles, 1950)	50 (Syracuse, 1990)
Nonnative	1 (Canandaigua, 1930)	15 (Syracuse, 1990)
Sediment-Tolerant	6 (Skaneateles, 1950)	37 (Syracuse, 1990)
Sediment-Intolerant	2 (Keuka, 1940)	15 (Seneca River, 1930)
Temperature-Tolerant	5 (Skaneateles, 1950)	34 (Syracuse, 1990)
Temperature-Intolerant	8 (Syracuse, 1940)	33 (Oneida North, 1990)

The numbers of species present in combinations of sub-basin and decade for each grouping of species type. Information in parenthesis indicates the site and decade of the observation

**Table 7:** Summary of the linear mixed effects models using different groupings of species.

Species Grouping	Land Cover	Fixed Effect	p-value
Native	Urban	1.11	**0.014**
Native	Agricultural	−0.39	**0.010**
Native	Natural	0.49	**0.016**
Nonnative	Urban	0.37	**0.0046**
Nonnative	Agricultural	−0.20	**0.036**
Nonnative	Natural	0.047	0.44
Sediment-Tolerant	Urban	1.15	**<0.001**
Sediment-Tolerant	Agricultural	−0.45	**<0.001**
Sediment-Tolerant	Natural	--	--
Sediment-Intolerant	Urban	0.20	0.14
Sediment-Intolerant	Agricultural	−0.071	0.076
Sediment-Intolerant	Natural	0.084	0.068
Temperature-Tolerant	Urban	1.15	**<0.001**
Temperature-Tolerant	Agricultural	−0.32	**0.0031**
Temperature-Tolerant	Natural	0.34	**0.030**
Temperature-Intolerant	Urban	0.36	0.16
Temperature-Intolerant	Agricultural	−0.17	**0.040**
Temperature-Intolerant	Natural	0.18	**0.047**

A summary table of the fixed effect and significance p-value for the model for each species grouping and each land cover type.

#### Model summaries by species grouping.

**Fig 12:**
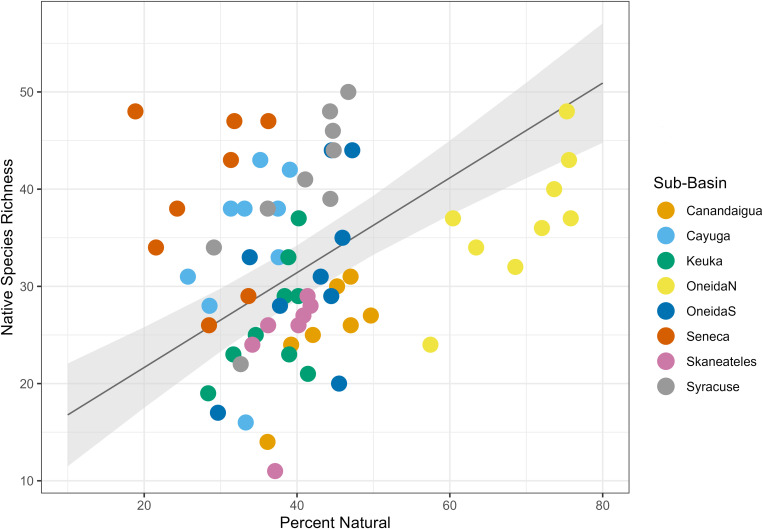
Natural land cover mixed effects model for native species. Plot of the linear mixed effects model results for the effect of percent natural land cover on native species richness. Gray shading shows standard error, and color coding on the data points indicates sub-basin.

**Fig 13:**
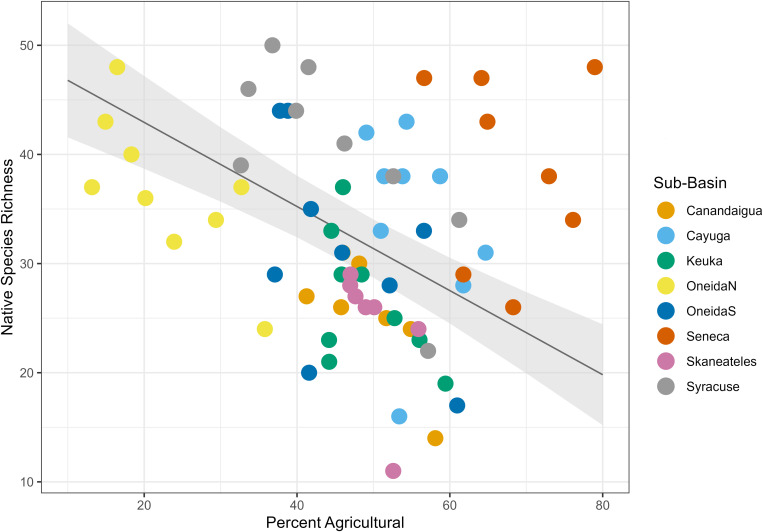
Agricultural land cover mixed effects model for native species. Plot of the linear mixed effects model results for the effect of percent agricultural land cover on native species richness. Gray shading shows standard error, and color coding on the data points indicates sub-basin.

**Fig 14:**
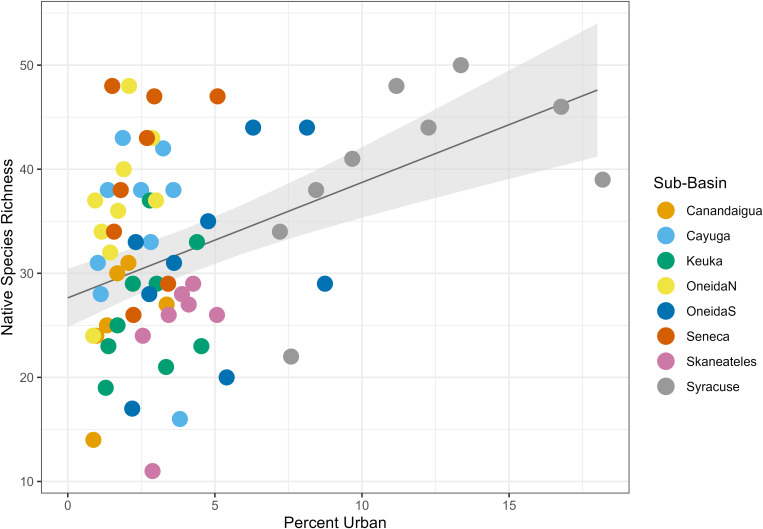
Urban land cover mixed effects model for native species. Plot of the linear mixed effects model results for the effect of percent urban land cover on native species richness. Gray shading shows standard error, and color coding on the data points indicates sub-basin.

**Fig 15:**
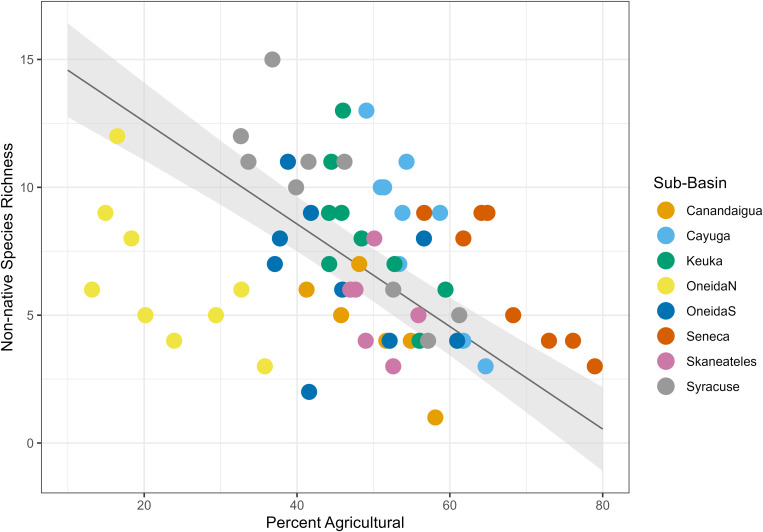
Urban land cover mixed effects model for non-native species. Plot of the linear mixed effects model results for the effect of percent urban land cover on non-native species richness. Gray shading shows standard error, and color coding on the data points indicates sub-basin.

**Fig 16:**
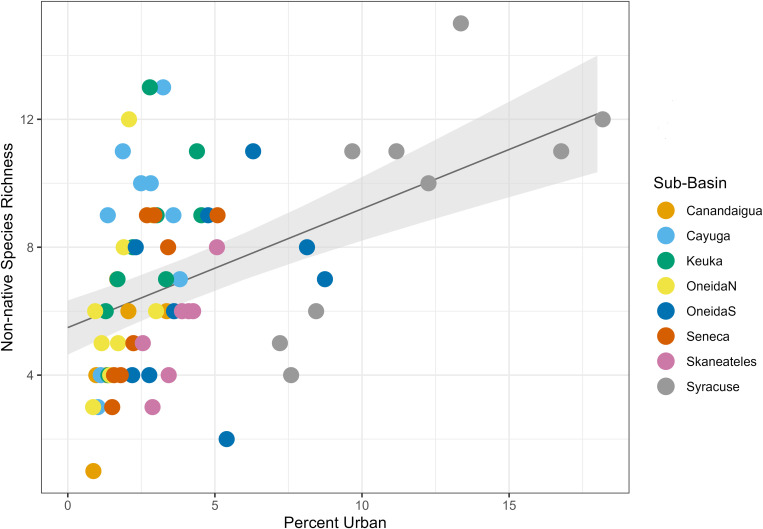
Agricultural land cover mixed effects model for non-native species. Plot of the linear mixed effects model results for the effect of percent agricultural land cover on non-native species richness. Gray shading shows standard error, and color coding on the data points indicates sub-basin.

Native species models displayed the same trends as the full species richness models. There was a positive relationship with natural land cover (p = 0.016), a negative relationship with agricultural land cover (p = 0.10), and a positive relationship with urban land cover (p = 0.014) (Table 7, Figs 12–14). Nonnative species had a significantly positive relationship with urban land cover (p = 0.0046), a negative relationship with agricultural land cover (p = 0.036) (Table 7, Figs 15 and 16), and no relationship with natural land cover (p = 0.44). The main divergence between these groups was that native species had a positive relationship with natural land cover, while the nonnative species did not.

**Fig 17:**
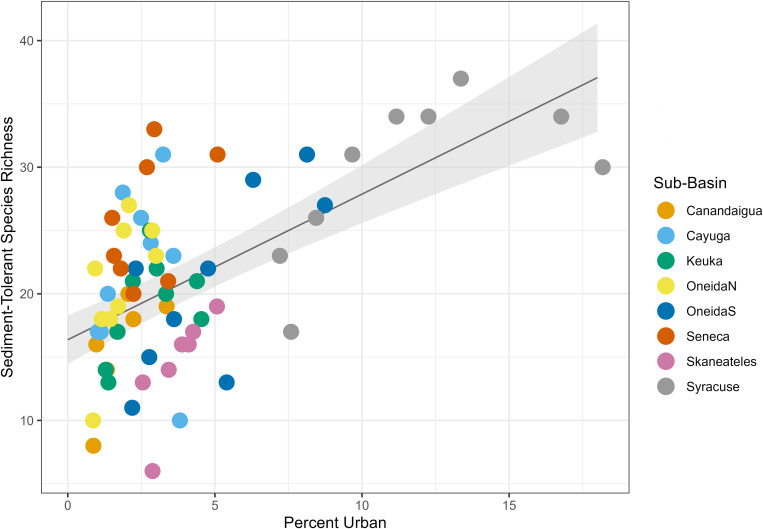
Urban land cover mixed effects model for sediment-tolerant species. Plot of the linear mixed effects model results for the effect of percent urban land cover on sediment-tolerant species richness. Gray shading shows standard error, and color coding on the data points indicates sub-basin.

**Fig 18:**
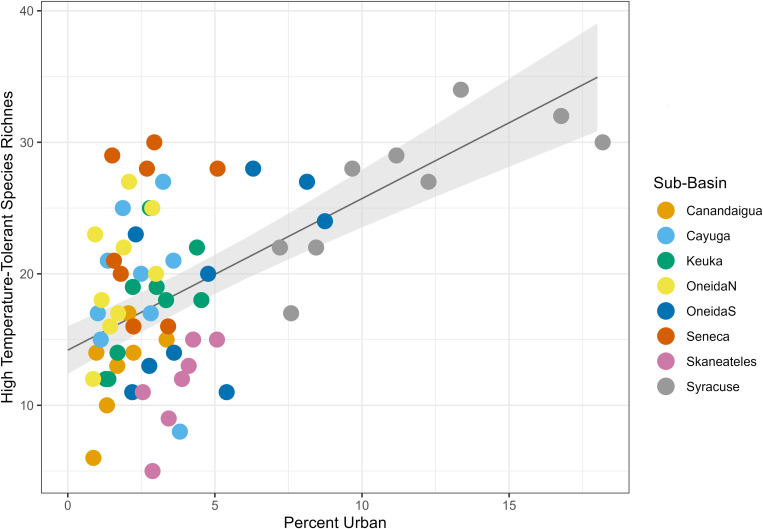
Urban land cover mixed effects model for temperature-tolerant species. Plot of the linear mixed effects model results for the effect of percent urban land cover on temperature-tolerant species richness. Gray shading shows standard error, and color coding on the data points indicates sub-basin.

In addition to these groups, sediment-tolerant species and temperature tolerant species both had positive relationships with urban land cover (p < 0.001 and p < 0.001 for both, Table 7, Figs 17 and 18). These was not a significant relationship between sediment-intolerant or high-temperature-intolerant species and urban land (p = 0.14 and p = 0.16).

#### Model summaries by land cover type.

Agricultural land cover had consistently negative correlations with species richness (Table 7). ANOVA comparisons with a null model were statistically significant for sediment tolerant species (p < 0.001), high-temperature-tolerant species (p = 0.003), high-temperature-intolerant species (p = 0.04), native species (p = 0.01), and nonnative species (p = 0.036). Only the model for sediment-intolerant species was statistically insignificant (p = 0.076).

Natural land cover had a consistently positive correlation with species richness (Table 7). The relationship was statistically significant for temperature-tolerant species (p = 0.03), temperature-intolerant species (p = 0.047), and native species (p = 0.016). The models were not significant for sediment-intolerant species (p = 0.068) or nonnative species (p = 0.44). A model could not be fitted for sediment-tolerant species due to a singular fit error (complexity of the model cannot be supported by the data).

As mentioned in the previous subsection, urban land cover had significant positive relationships with sediment-tolerant (p < 0.001), temperature-tolerant (p < 0.001), native (p = 0.014), and nonnative species (p = 0.0046) (Table 7), but not with sediment-intolerant (p = 0.14) or temperature-intolerant (p = 0.16) species.

## Discussion

Our results indicate that a near century of change in land cover patterns across the Oswego River Basin has had an impact on the species richness of fishes present within those waters. Specifically, we found a strong positive relationship between the increase in natural land cover and species richness, and an associated negative relationship between agriculture and decreased species richness. Perhaps counterintuitively we also found a positive relationship among changes in urban land cover and species richness, indicating the complexity of relationships within urban ecosystems. These trends held steady in models created using individual groupings of species, even though those models included less data, and in models using buffer land cover only. Carlson & Daniels [23] found an increase in species richness over time in the Oswego watershed, similar to our results; both studies are based upon extensive historical data emphasizing the importance of long-term data in contextualizing present day species distributions.

### Land cover effects

Consistent with the published literature, our results suggest species richness is positively associated with natural landscapes and declines are evident with increases in agricultural land use [41,42]. Other studies have shown that agricultural land cover has indirect negative impacts on stream habitat and biotic integrity, and these effects are long-lasting even when land cover changes [43]. Agricultural land cover is associated with altered temperatures and hydrology; channelization and major impacts on stream geometry; and increases in nutrient, sediment, and pesticide pollution, so it is unsurprising that many species would be negatively impacted [1,3–5,44].

In contrast, increases in species richness associated with increased urban land cover is counterintuitive [9,13,41]. There are several explanations for our findings of higher species richness with more urban land cover. The first is that areas with natural land cover in riparian buffers within the urban zone may represent more suitable habitat for aquatic species than agricultural land, as observed for aquatic macroinvertebrates [45]. Additionally, although urban land cover was low in the early 20th century, the watershed was impacted by water pollution [46]. Increases in urbanization occurred along with environmental legislation such as the Clean Water Act, ameliorating the effects of water pollution on fish communities. It is also worth noting that while changes in natural land cover and the associated changes in species richness occurred over larger spatial areas, the changes in urban land cover represented a doubling of urban land in most sub-basins but was still only a small percentage of total land cover (Table 1).

Another explanation is that stocking can artificially raise fish richness, disconnected from any landscape effects. The NYSDEC supports a large recreational freshwater fishery in the state by stocking more than 200 million fish per year in 1,200 streams, lakes, rivers, and ponds across New York State, representing 20 species [47]. Hours of angler use is one criterion for stocking, and the NYSDEC stocks trout in both coldwater streams with natural trout populations and streams where summer temperatures or habitat quality do not allow natural populations to persist [48]. The stocked species are a mix of native species such as brook trout (*Salvelinus fontinalis*) and lake trout (*S. namaycush*), and nonnative species such as brown trout (*Salmo trutta*) and Chinook salmon (*Oncorhynchus tshawytscha*) [49]. If species are preferentially stocked near areas with higher human populations, e.g., salmonids stocked in the region around Syracuse, a major metropolitan area, this could result in an increase in species richness.

In addition, two distinct features of the Syracuse sub-basin may increase species richness there, despite it having the highest proportion of urban land cover in the study. First, the sub-basin also includes the Oswego River’s outlet to Lake Ontario and could be influenced by fish populations in the lake; for example, Chinook salmon and coho salmon (*O. kisutch*), from Lake Ontario, were only detected in the Syracuse sub-basin in the Oswego River watershed. Second, numerous coldwater springs around the city make streams such as Ninemile Creek suitable for species such as trout [50], potentially mitigating the effects of increased water temperatures often associated with urban centers.

The success of tolerant and nonnative species in disturbed areas could also be responsible for an increase in species richness. Urban land cover models could make significant predictions for sediment-tolerant species but not sediment-intolerant species and temperature-tolerant but not temperature-intolerant species (Table 7). At the HUC-8 watershed scale across North America, [51] found that anthropogenic activity correlated with higher numbers of invasive fish species, which they theorized was due to habitat disturbance and increased propagule pressure near higher human populations. These disturbed ecosystems may offer niches where tolerant species can be successful due to reduced competition from more sensitive native species, and where introduced species can secure habitat. This corresponds with our findings of an increased proportion of nonnative fish in the assemblage over time in the Oswego River Watershed.

Another takeaway from our findings is that our graphical analyses did not show a threshold, or “tipping point”, in the relationship between species richness and natural land cover (Fig 4). This result suggests that any degree of habitat restoration may cause an increase in fish species richness in the area, rather than a specific threshold needing to be reached in order for the project to have the potential for success.

Finally, historical data are valuable for understanding the phenomenon of shifting baselines, where each generation takes the state of the ecosystem at the beginning of their life as ‘normal’ but changes over longer time scales go unnoticed. Shifting baselines have been well-documented in marine fisheries [52,53], as well as freshwater systems [54], and understanding true baselines is important for effective conservation [55]

### Land cover effect limitations

**Fig 19:**
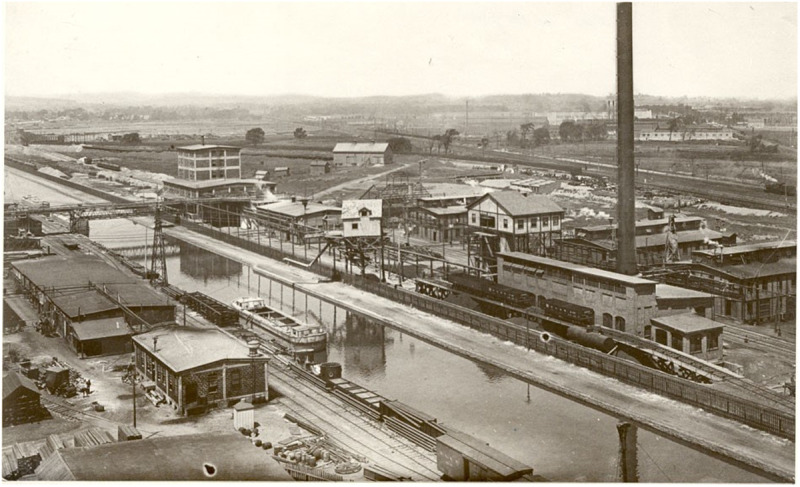
The Solvay Process Plant on the Erie Canal in the early 1900s. Photo from the Solvay Process, available from the Library of Congress. Detroit Publishing Co, P. Solvay Process Co.‘s works, Syracuse, i.e., Solvary. Solvay United States New York State New York, None. [Between 1890 and 1901] [Photograph] Retrieved from the Library of Congress, https://www.loc.gov/item/2016801688/.

**Fig 20:**
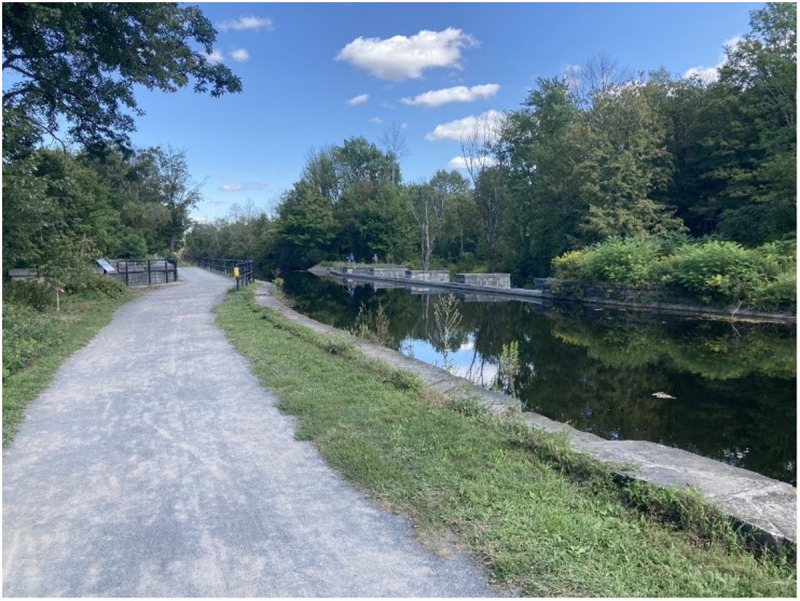
The Ninemile Creek Aqueduct in 2021. Photo taken approximately 6 kilometers west of Solvay and 500 meters north of Highway 5. Photo taken by the authors.

Land cover change tells only part of the story of shifting species richness in the Oswego River Watershed. While most linear mixed effect models were statistically significant in comparison to a null model, land cover is not the only factor that influences an aquatic system, and the increase in species richness with urban land cover could be partially or completely driven by factors not included in our models. We included decade as a random effect in our models to account for time, but in the final models, decade often explained only a small portion of the variance. Since overall species richness increased over the course of the study due to factors other than land cover, the models may not have fully accounted for the effect of time. One major factor is an improvement in water quality over the course of the dataset—while the amount of urban land cover was lower in the 1920s, that urban land was much more polluted than today’s larger developed areas. Anthropogenic pollution has had a major influence on aquatic communities in the Oswego River Watershed specifically, and in New York as a whole, for well over a century. For example, the 1927 watershed survey identified seven major categories of pollution—sewage, paper mill waste, wool mill waste, cannery waste, oil, cannery waste, and sulfur—that polluted 108 miles of streams and the outlets of the majority of the Finger Lakes [56]. Creameries and milk plants had polluted headwaters prior to the study, but most had stopped operating before 1920 [56]. However, the toxic legacy of these pollution sources can remain [57]. Industrial pollution and sewage were also major problems that made parts of the watershed unsuitable for aquatic life-- [46] characterized a stream near Rome, New York that received raw sewage from the city as a “veritable open sewer” that was “uninhabitable to fish life for miles”, while Ninemile Creek and the Erie Canal ran through the Solvay Process soda ash plant, which would become a major source of pollution in Onondaga Lake (Figs 19 and 20). Aquatic conditions have improved in the subsequent century due to stricter pollution control measures and legislation such as the Clean Water Act. For example, superfund site Onondaga Lake has experienced improved water quality in the latter half of the 20^th^ century due to stricter regulations and improved wastewater treatment. Species richness has increased, although the lake continues to exceed pollution standards and fish populations rely on recruitment from and stocking in surrounding water bodies [58,59]. It is important to note that different levels of anthropogenic impact can exist within a land cover type, and these intensities have varied throughout the watershed with time. In the case of the Oswego River drainage it appears that while the amount of urban landcover has increased, the more modern urban landscape has a suite of features that reduce the environmental impact when compared to the intensive industry of early 19^th^ century cities.

## Conclusions

A large historical database such as the one that exists for the Oswego River watershed provides excellent opportunities for investigating long-term change, which is one tool for improving our ability to predict the impacts of future changes. The State of New York’s continued efforts to add to this dataset will also improve our ability to make understand changes that have occurred in the region, including those about subgroupings of fish, such as temperature-intolerant species or native species. Information on historical trends relating land cover to species richness is another source of information that can be used in models such as the USGS Aquatic GAP that have the goal of evaluating aquatic biodiversity to support more effective species conservation. Applied historical ecology is an important tool for understanding dynamic ecosystems, and a better understanding of historical trends is important for making informed management decisions [60]. Historical ecology is also important because historical data sources can generate unexpected results that allow for new hypotheses to be developed and tested [21]. From our study, urban aquatic ecology is an interesting area for future study, examining forces driving increased species richness in areas with more urban land cover—for example, potential increases in tolerant and nonnative species. In our study, the urban increases seem to be driven most strongly by high species richness in area surrounding Syracuse and going through the outlet to Lake Ontario; those are ideal sites for future research.

## Supporting information

S1 Fig Fish data available by decade. Bars depict the number of fish sample records in the NY Fish Atlas database for each decade from the 1920s to the 2010s.
(DOCX)

S2 Fig Residual plots. Q-Q plots for normal distribution of the residuals for the models for A. Species richness and agricultural land cover, B. Species richness and natural land cover, C. Species richness and urban land cover.
(DOCX)

S3 Fig Centrarchidae timelines. Presence timelines for members of the family Centrarchidae over time in (A) the entire Oswego River Watershed, (B) the Cayuga sub-basin, (C) the Oneida North sub-basin, and (D) the Syracuse sub-basin.
(DOCX)

S1 Table Species classifications for analysis. Sediment tolerance from Trebitz et al. (2007) (marked with 1) and Whittier and Hughes (1998) (marked with 2), critical temperatures from Hazlett (2021) (marked with *), and species origins from the USGS Nonindigenous Aquatic Species List (marked with 3) and the *Atlas of the Inland Fishes of New York* (marked with 4).
(DOCX)

S2 Table Fish observations and species richness by site and decade. The number of fish counted in surveys in each sub-basin of the watershed during each decade, and the species richness from that count. Combinations of sub-basin and decade with fewer than 50 observations were excluded from the analysis for a lack of data, since these species counts are likely artificially low.
(DOCX)

S3 Table Full and reduced model comparisons Comparison between the results of linear mixed effects models using the full dataset (all site-decade combinations with at least 50 observations) and the reduced/rarefied dataset (only site-decade combinations with at least 250 observations, and only a random subset of 250 observations used for site-decade combinations with larger numbers of observations).
(DOCX)

S4 Table Sources of random variation in each model. Tables depict the amount of variance explained by decade, sub-basin, and residuals for each model.
(DOCX)

S5 Table Species gains and losses over time in each sub-basin of the Oswego River Watershed. Tables for each sub-basin showing species added (appeared in the watershed for the first time), reappeared (present after having been absent for the previous decade), rejoined (present after having been absent for two or more decades), remained, missing (absent after being present in the previous decade), and lost (absent for two consecutive decades).
(DOCX)

S6 Table Tracking the presence or absence of each species through the full watershed and each sub-basin over time. Table tracking presence and absence for each individual species in the full watershed and then in each sub-basin. A name indicates that the species was present, and a blank cell indicates that the species was absent.
(DOCX)

S7 Table Results of the linear mixed effects model for each grouping of species. Tables showing the fixed effect, standard error of the fixed effect, fixed effect intercept, standard error of the fixed effect intercept, and ANOVA p-value (α = 0.05) for the models for each land cover type for each species grouping.
(DOCX)

S8 Table Full and Buffer Models. Comparing linear mixed effect model results for full land cover and buffer land cover.
(DOCX)
