# Supplementary material for: Land cover as a driver of fish community changes in New York’s Oswego River Watershed
Source: PLoS One. 2025 Jul 14;20(7):e0327293. doi: 10.1371/journal.pone.0327293 (PMC12258583; doi:10.1371/journal.pone.0327293)

**S1 Figure.**  **Fish data available by decade.** Bars depict the number of fish sample records in the NY Fish Atlas database for each decade from the 1920s to the 2010s.


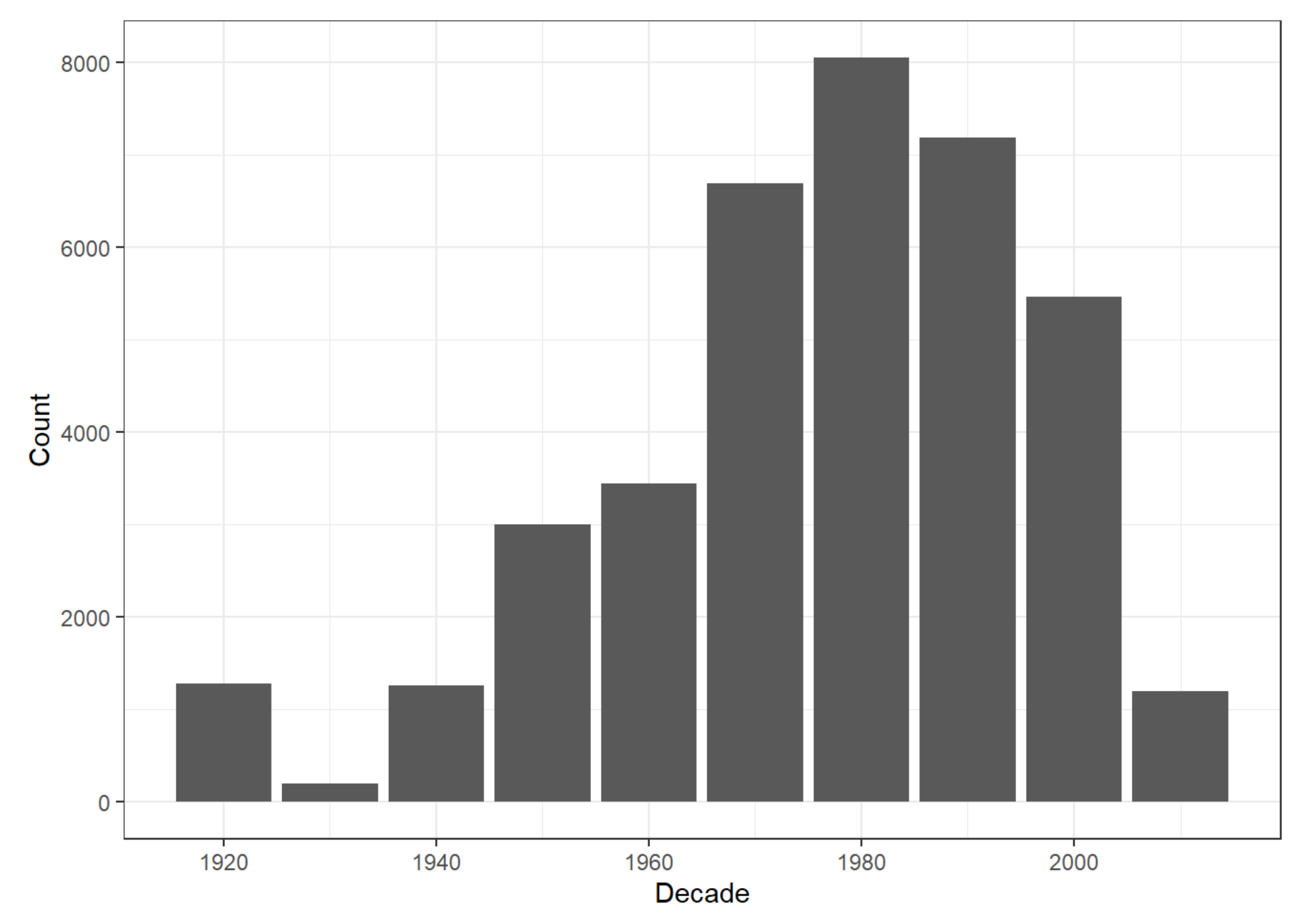

Supplement: S1 Fig — Bars depict the number of fish sample records in the NY Fish Atlas database for each decade from the 1920s to the 2010s. (DOCX) [file pone.0327293.s001.docx]
