# Supplementary material for: Land cover as a driver of fish community changes in New York’s Oswego River Watershed
Source: PLoS One. 2025 Jul 14;20(7):e0327293. doi: 10.1371/journal.pone.0327293 (PMC12258583; doi:10.1371/journal.pone.0327293)

**S2 Figure. Residual plots.** Q-Q plots for normal distribution of the residuals for the models for A. Species richness and agricultural land cover, B. Species richness and natural land cover, C. Species richness and urban land cover.

Full Species Richness


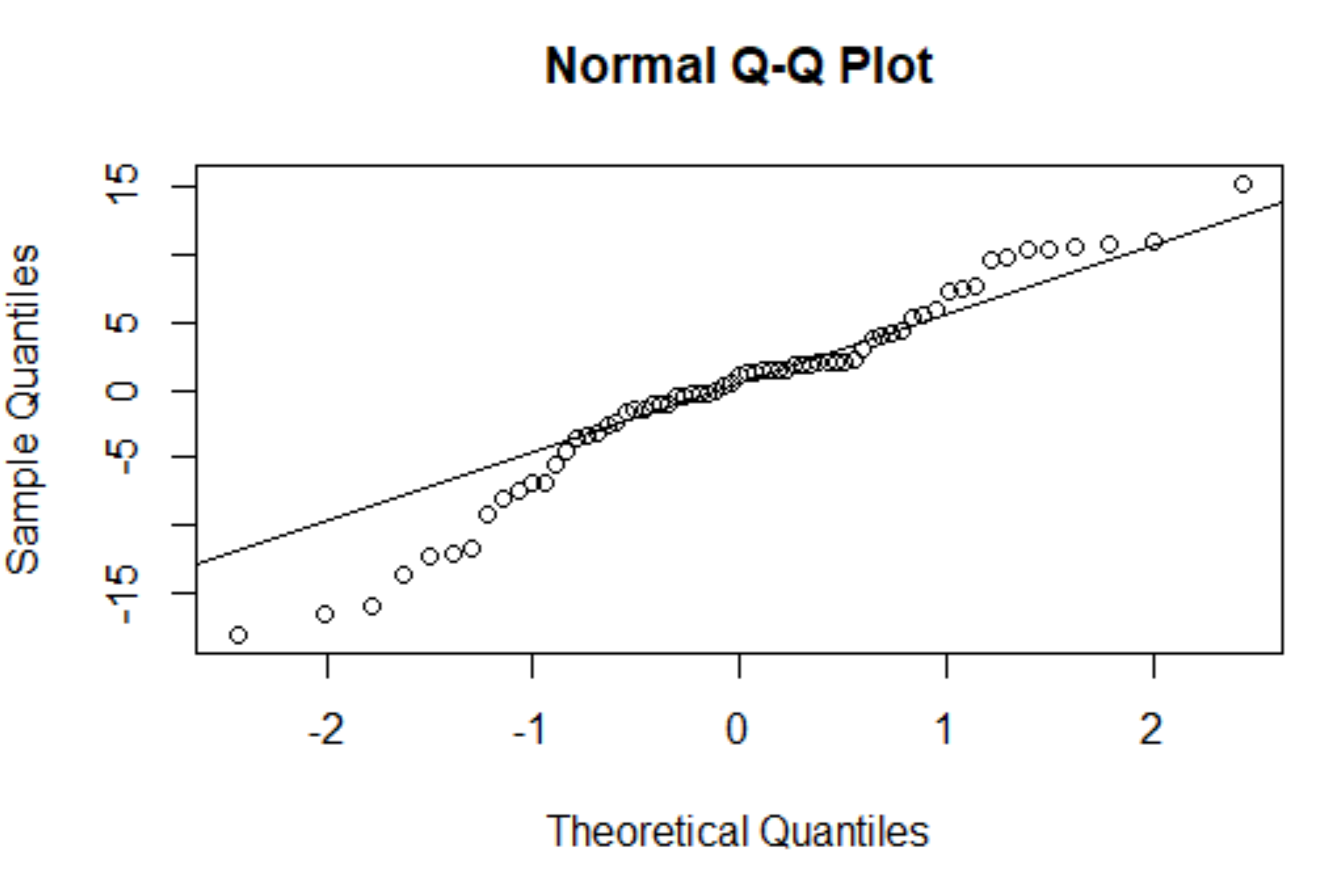

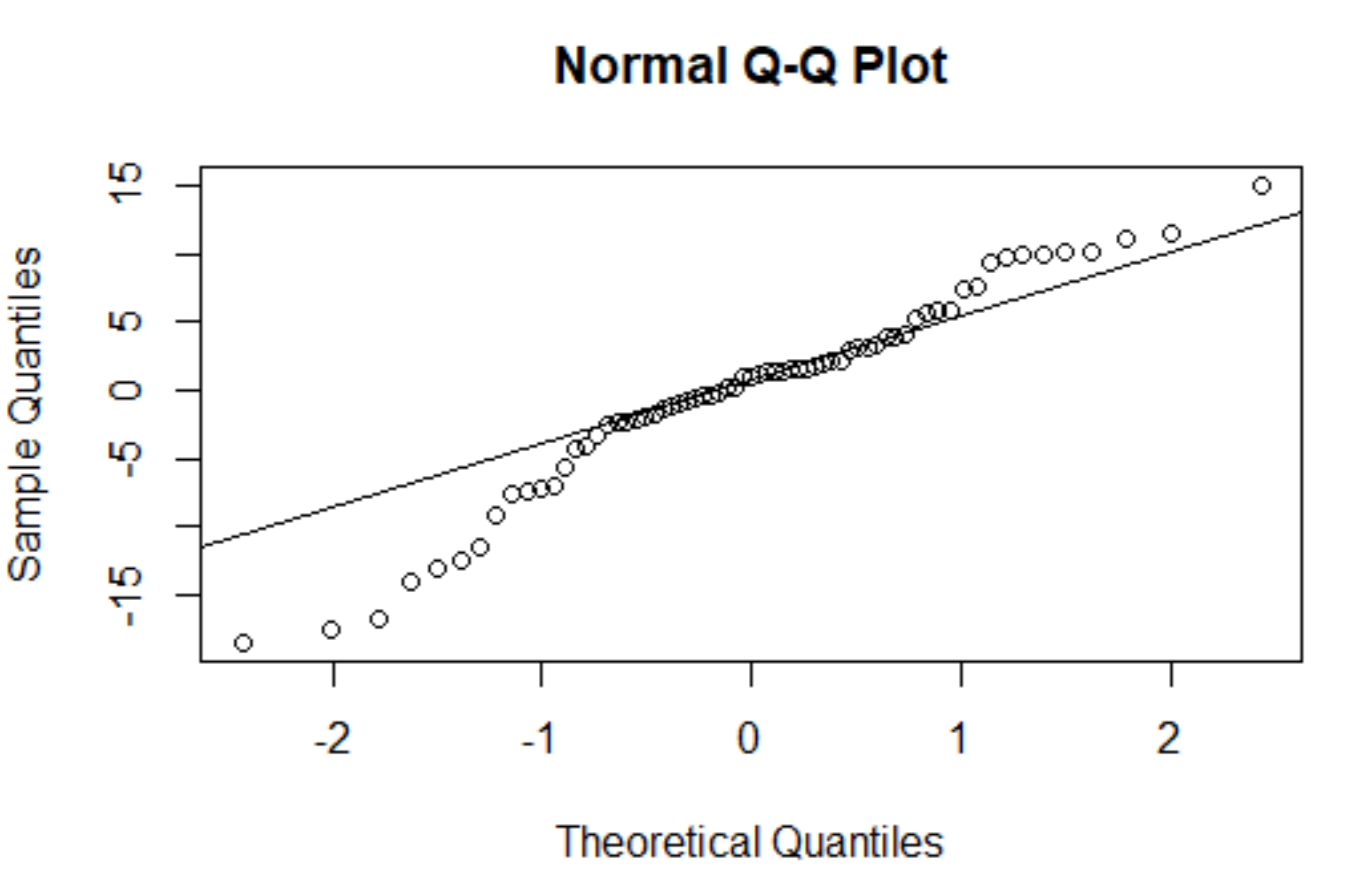


**B**

**A**


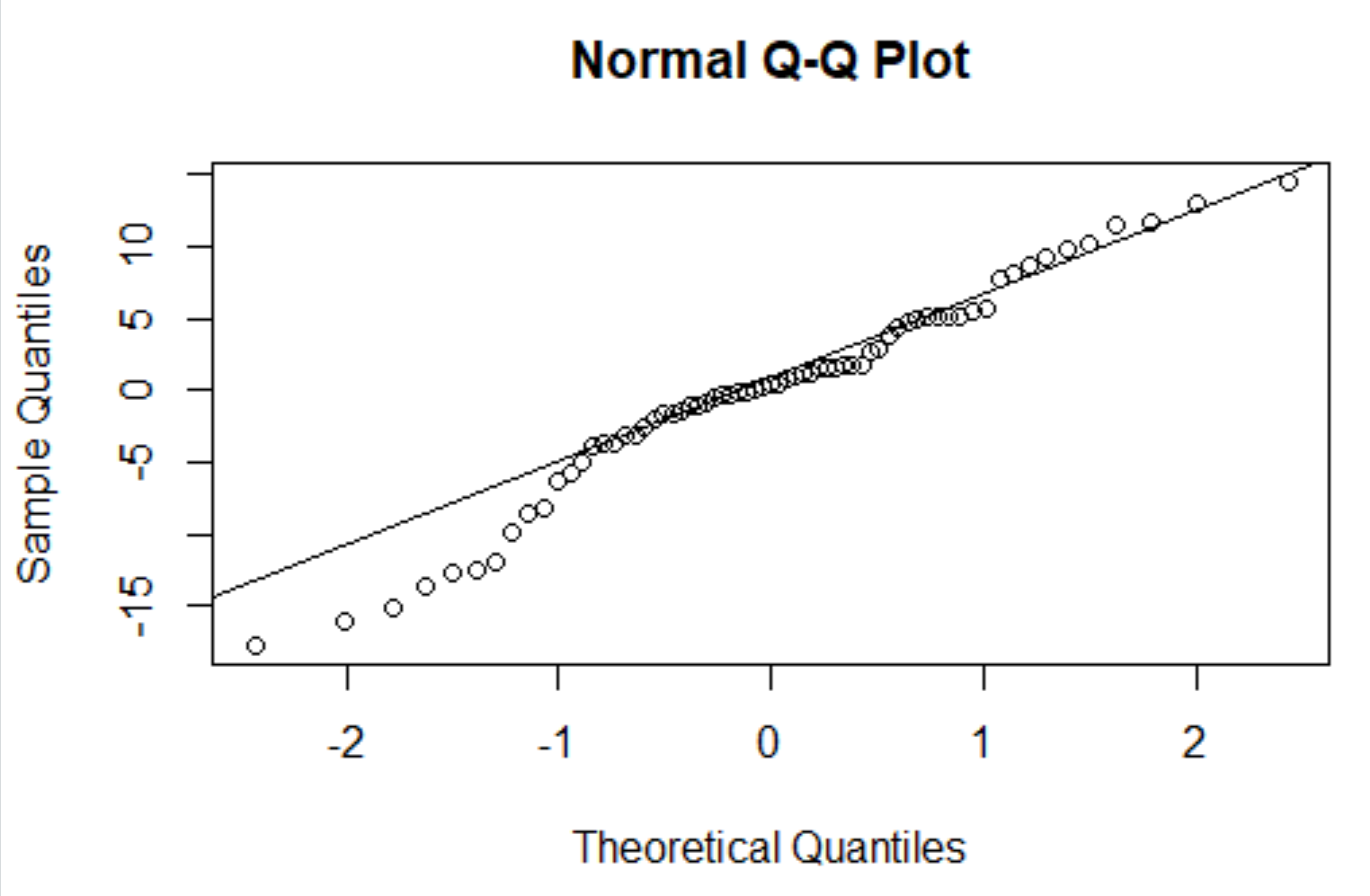


**C**

Sediment-Tolerant Species Richness


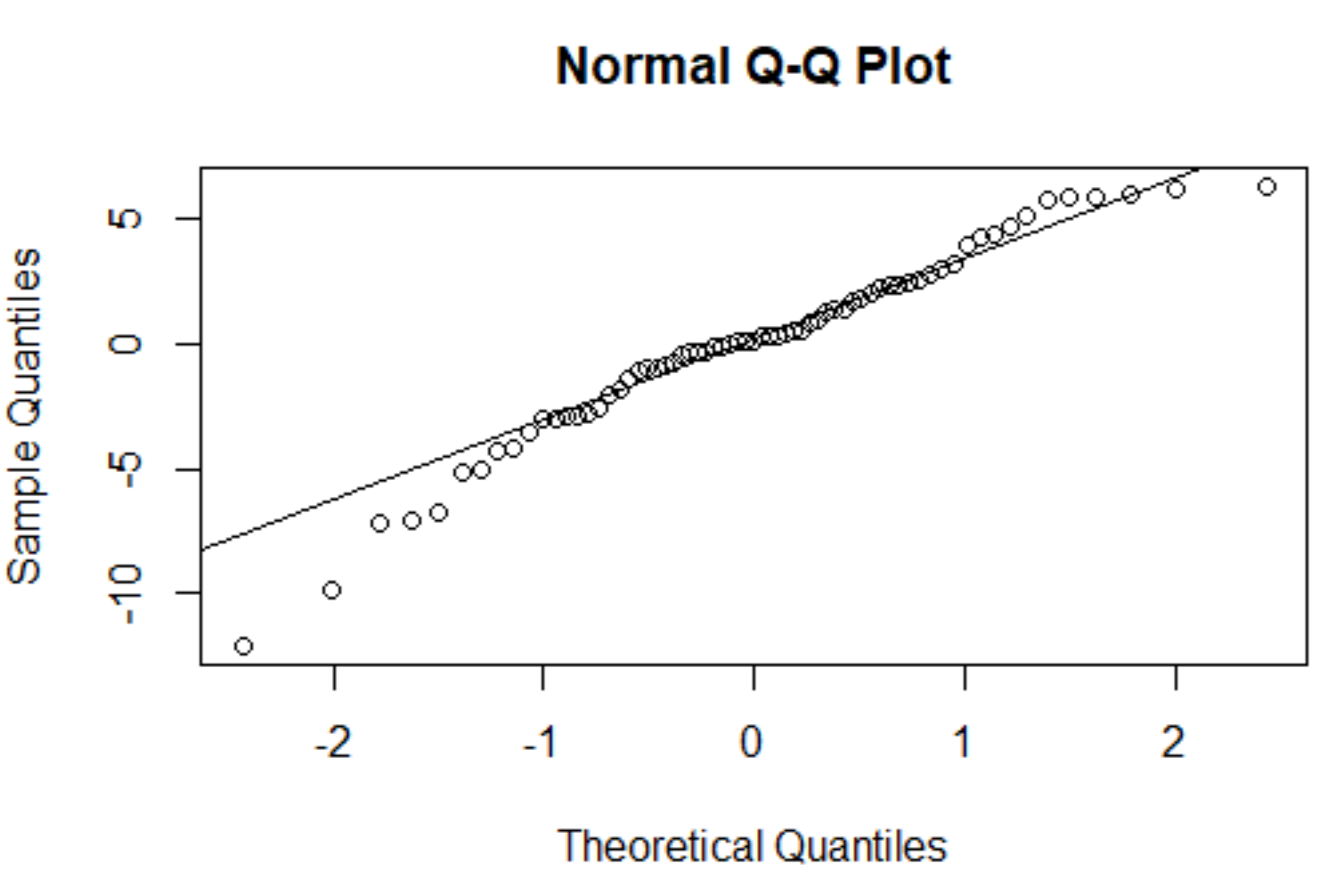

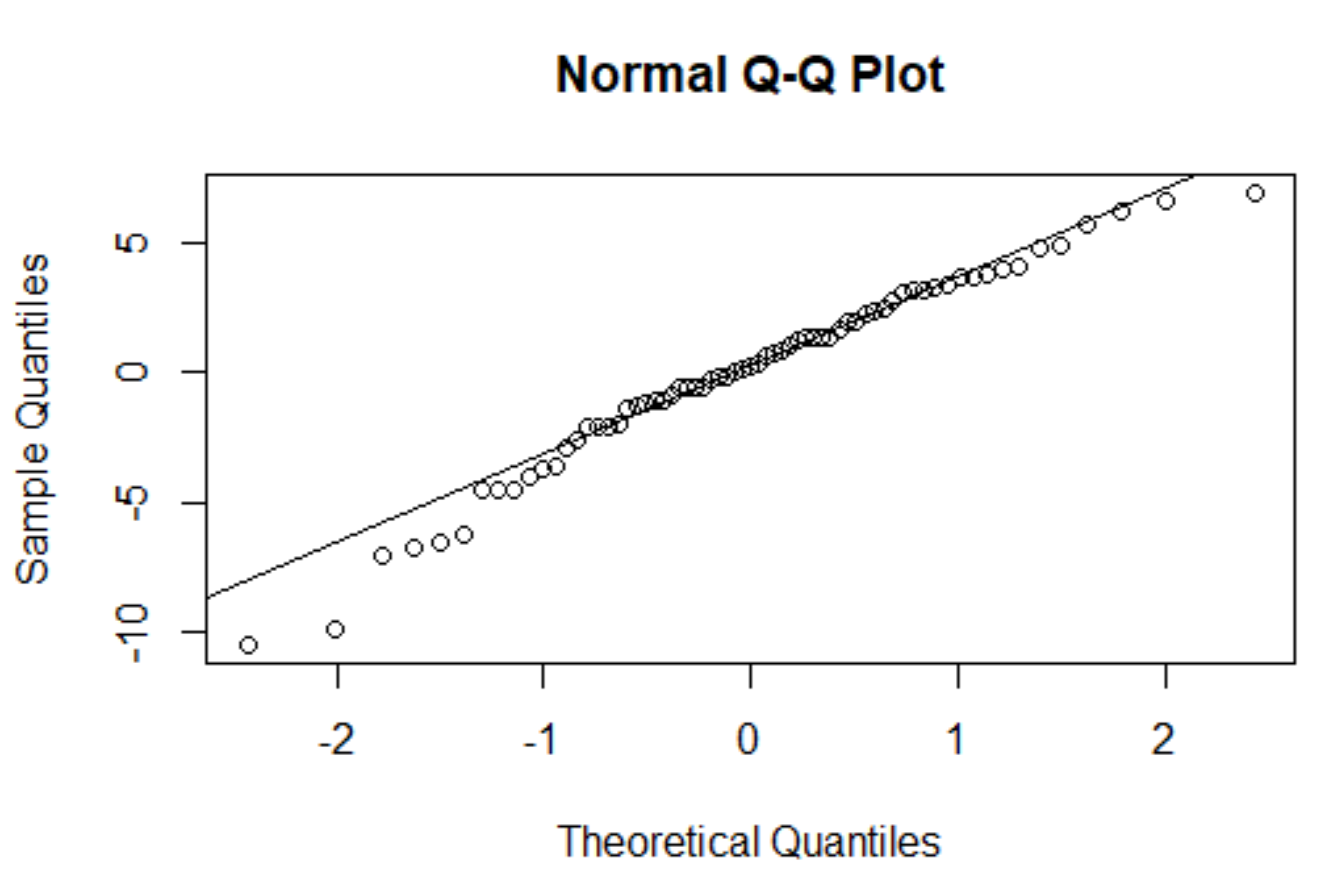


**A**

**C**

Sediment-Intolerant Species Richness


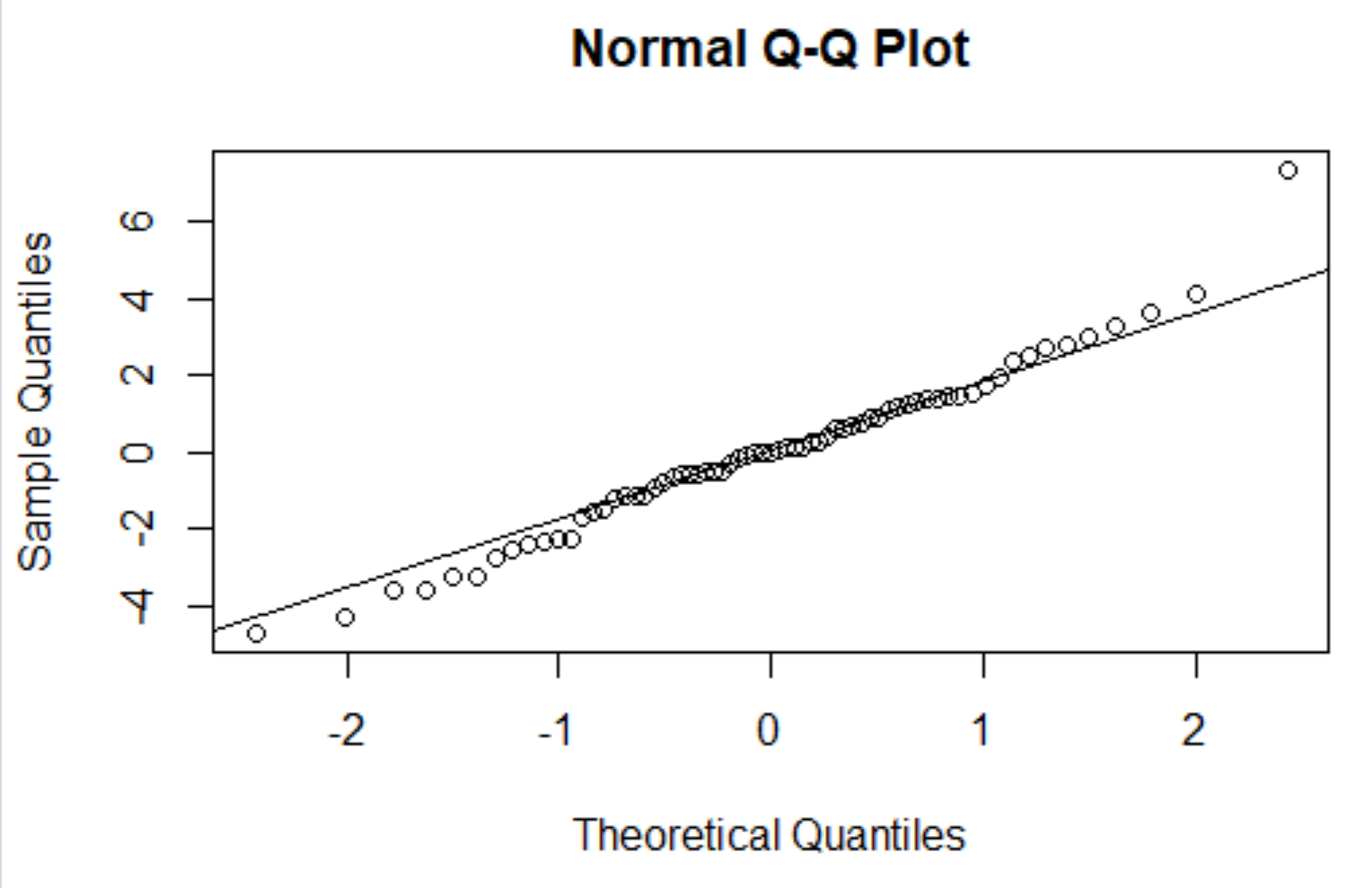

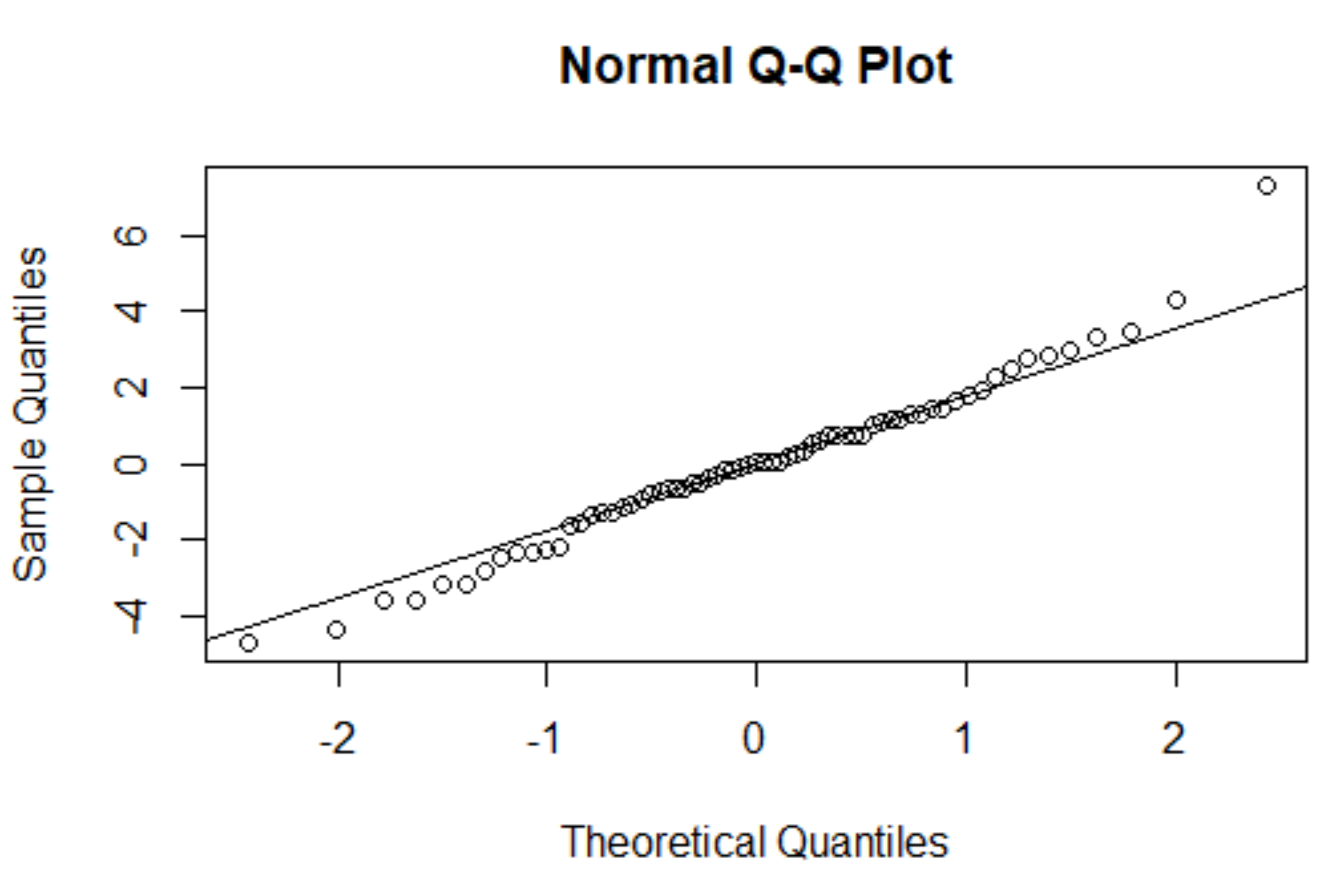


**B**

**A**


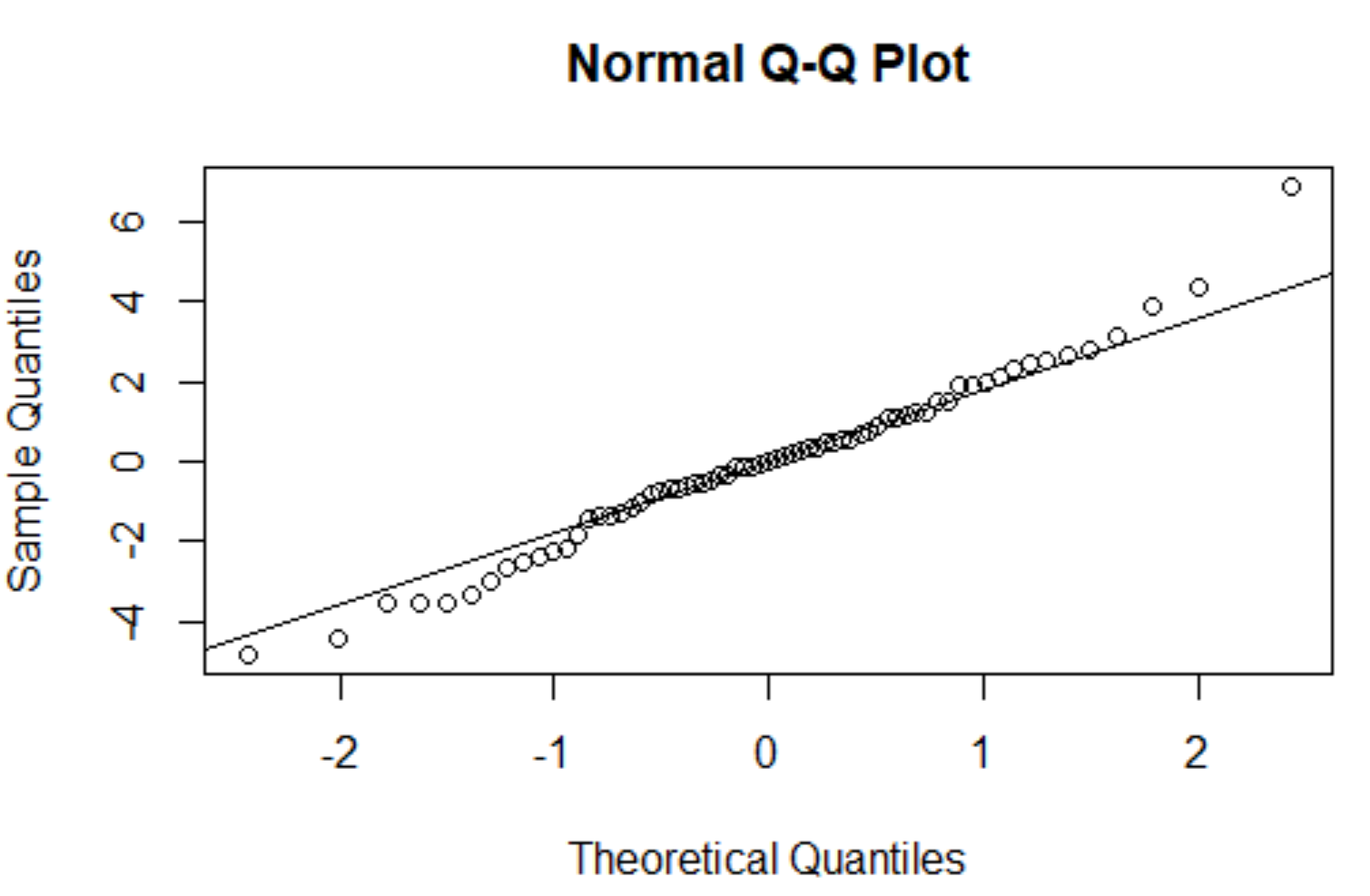


**C**

High-Temperature-Tolerant Species Richness


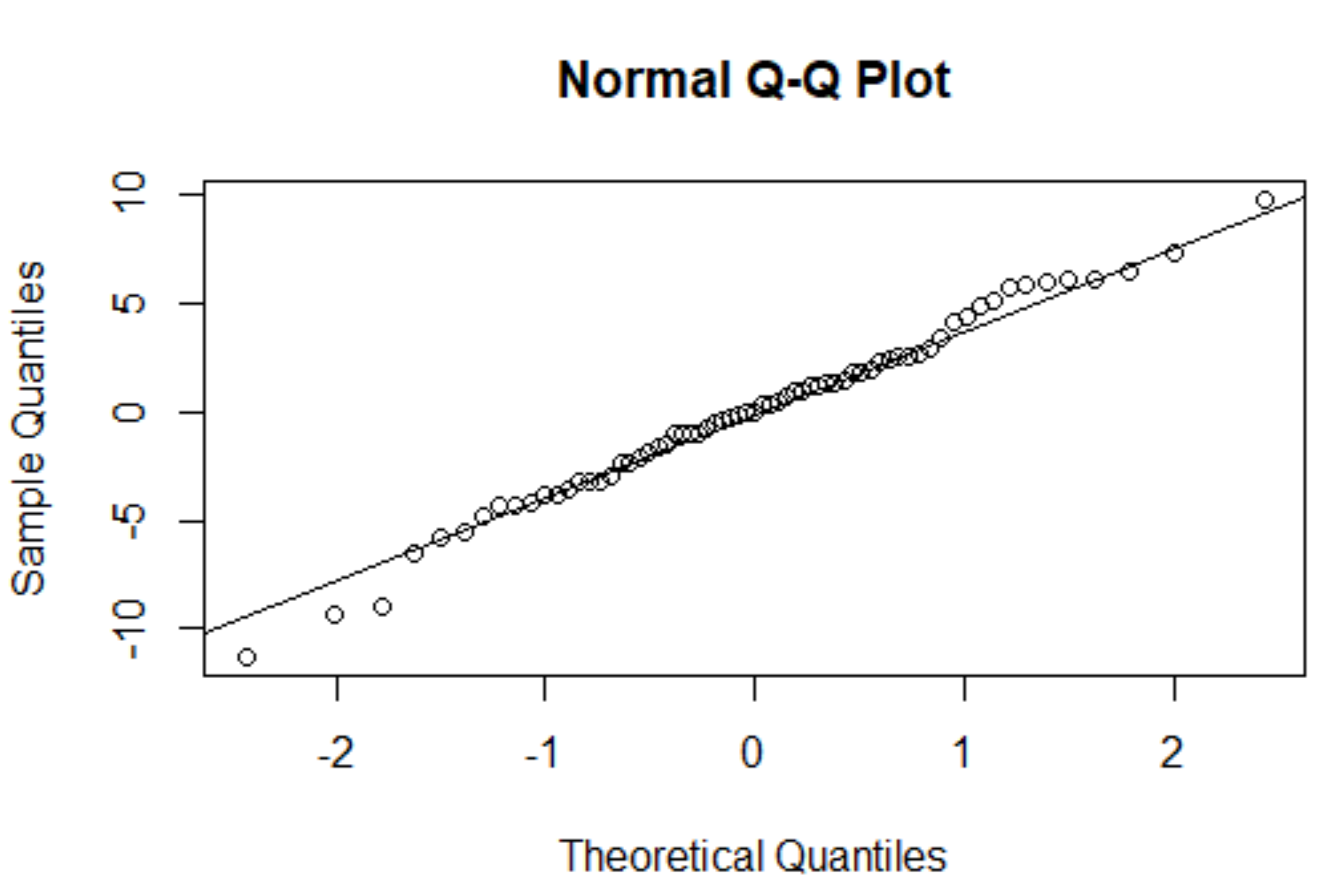

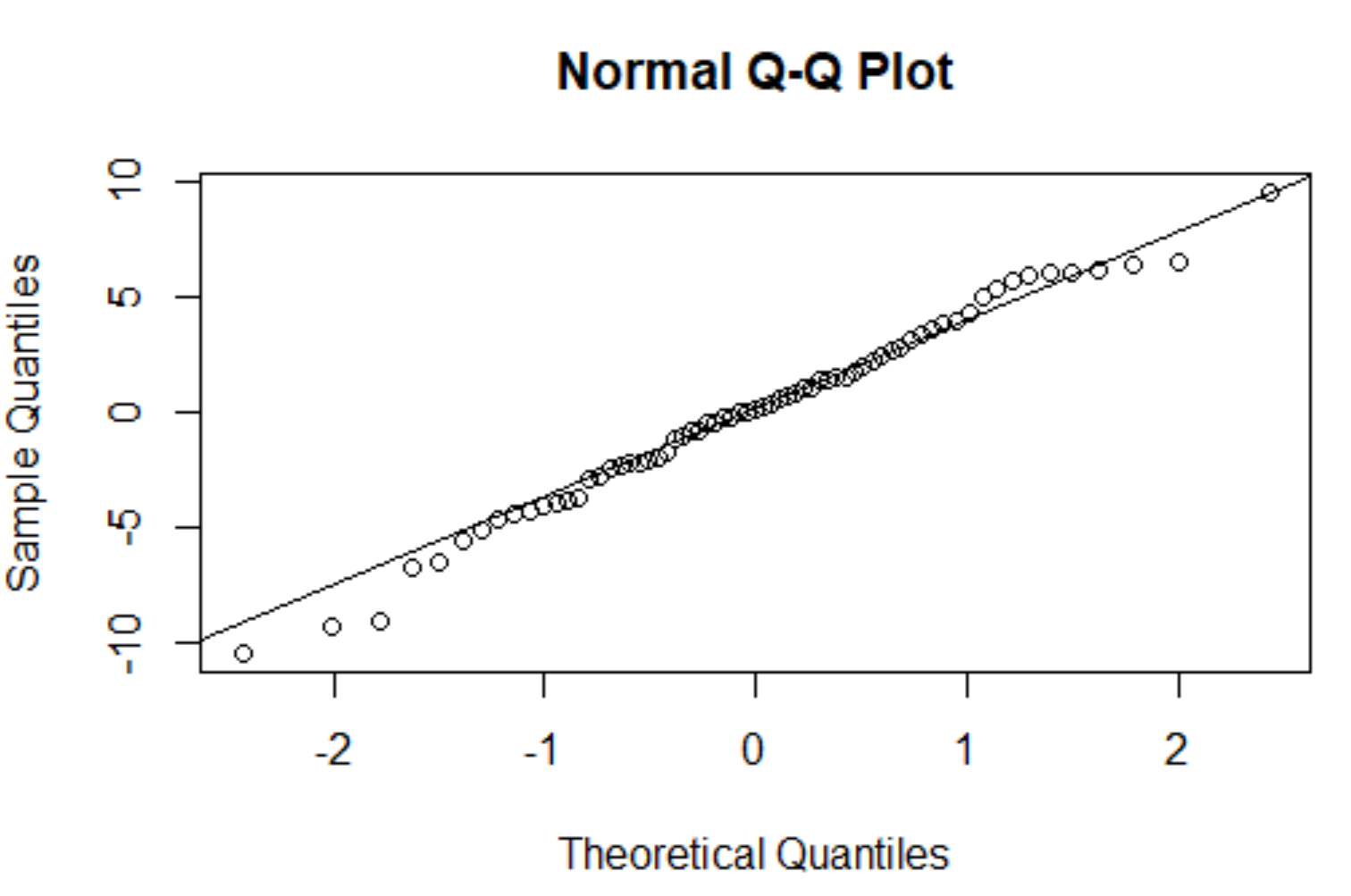


**B**

**A**


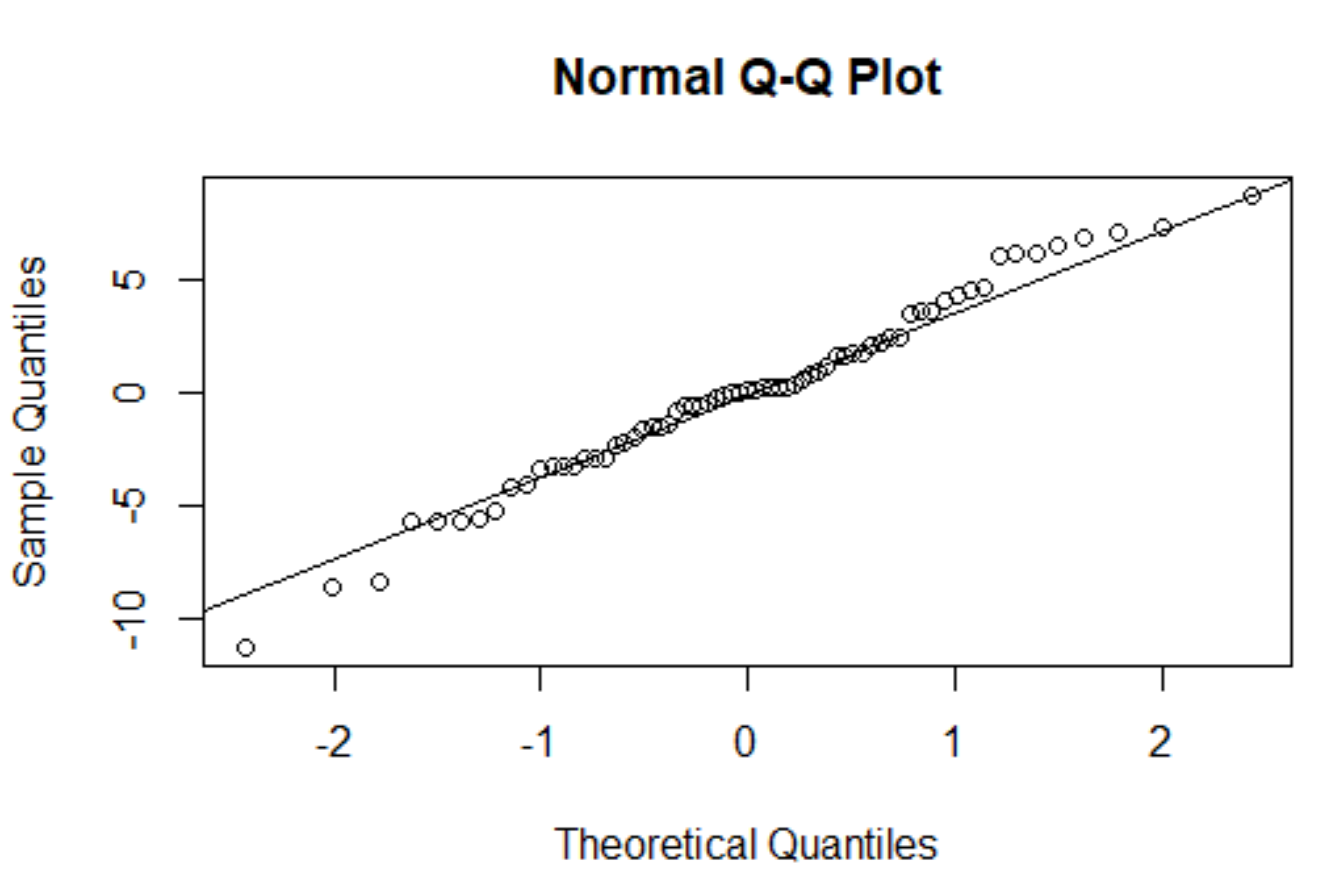


**C**

High-Temperature-Intolerant Species


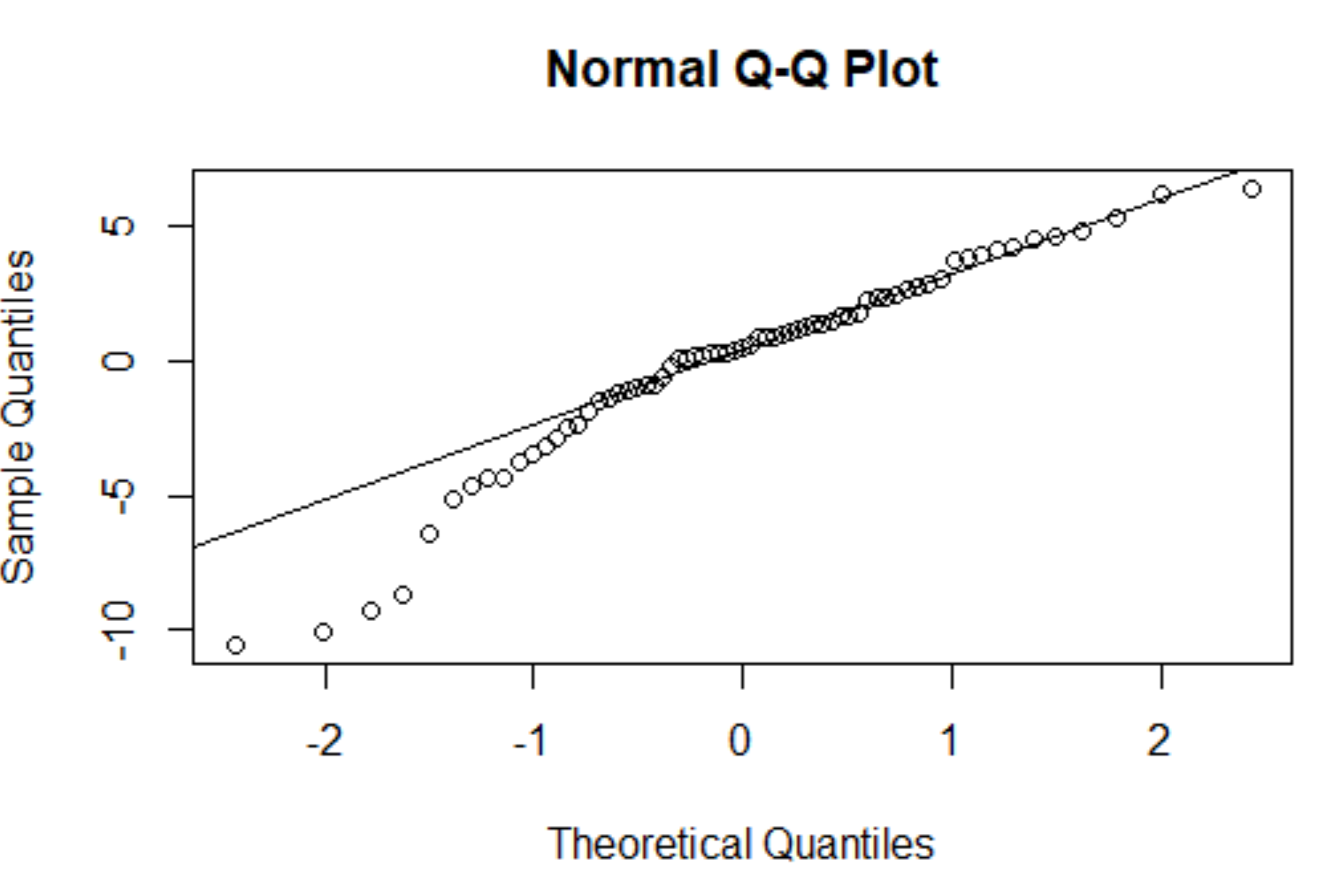

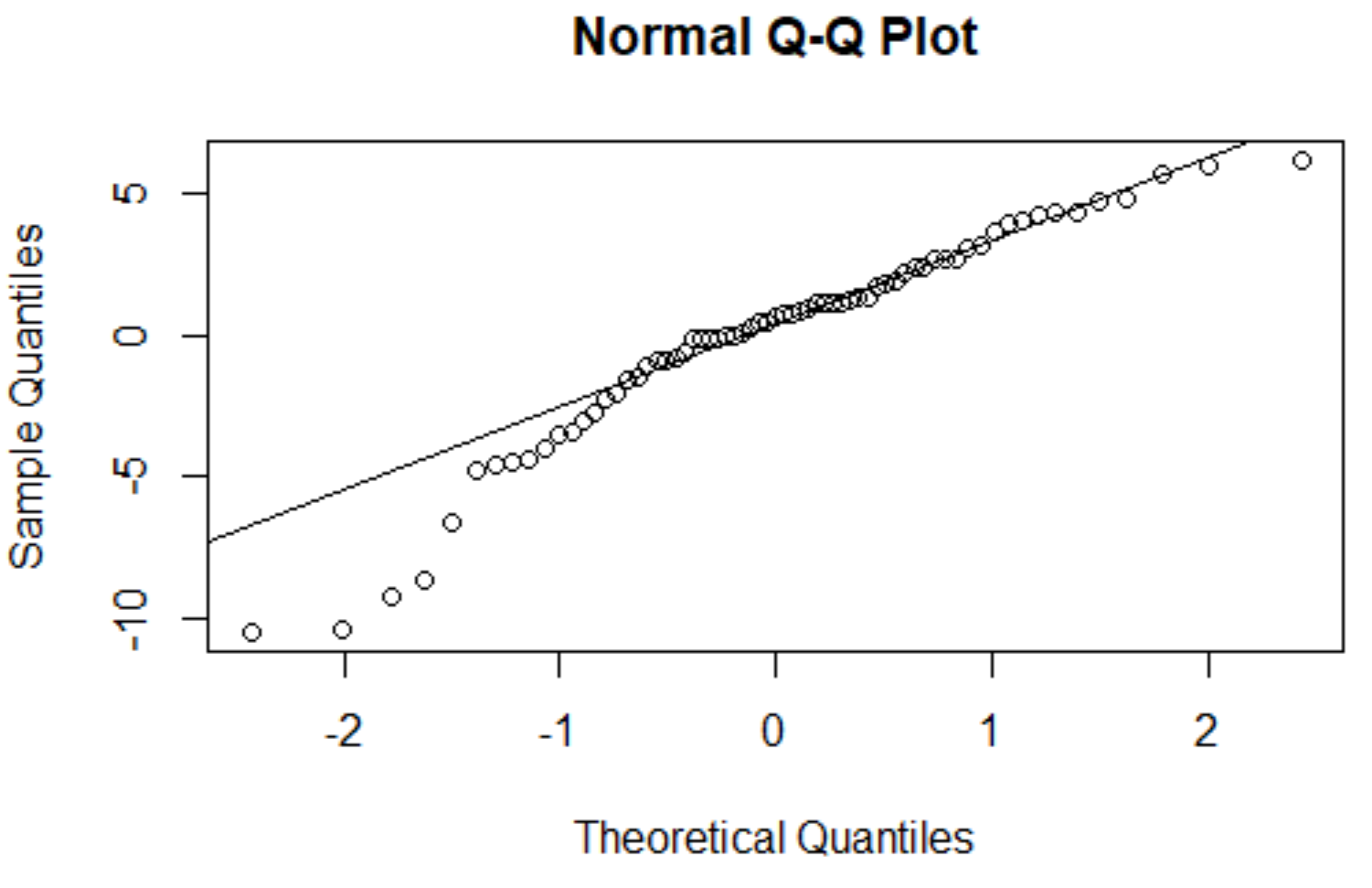


**A**
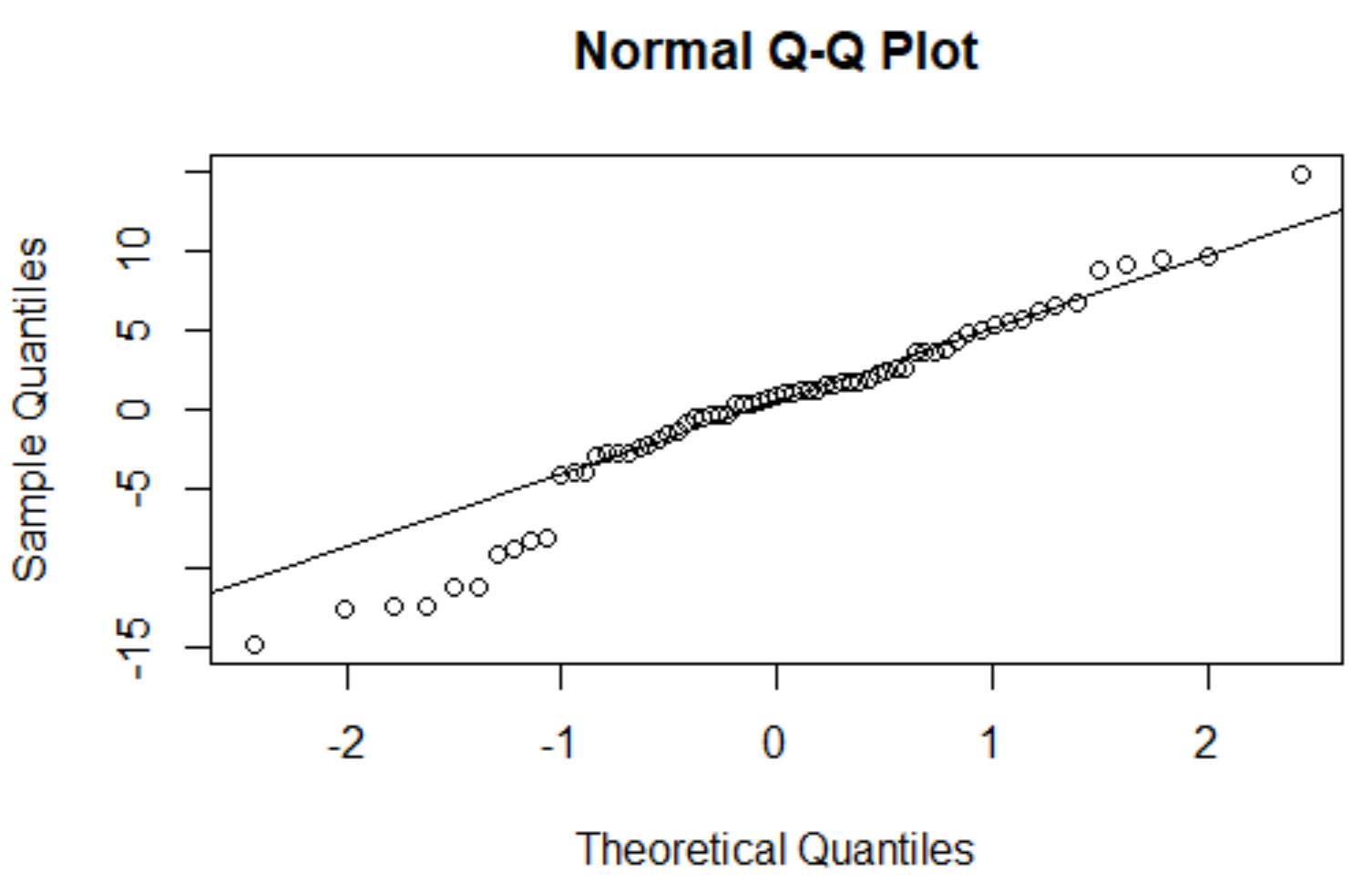


**B**


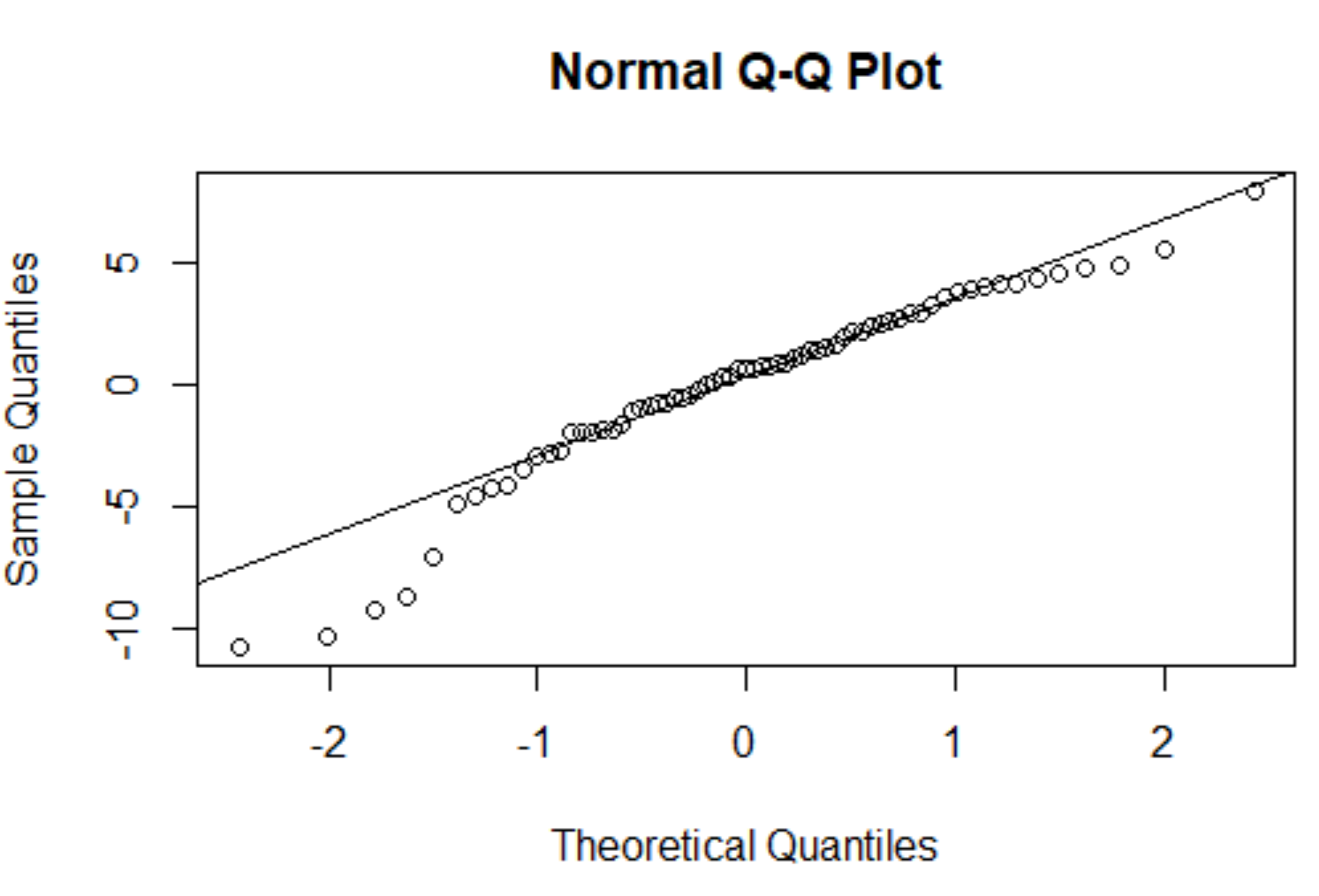


**C**

Native Species Richness


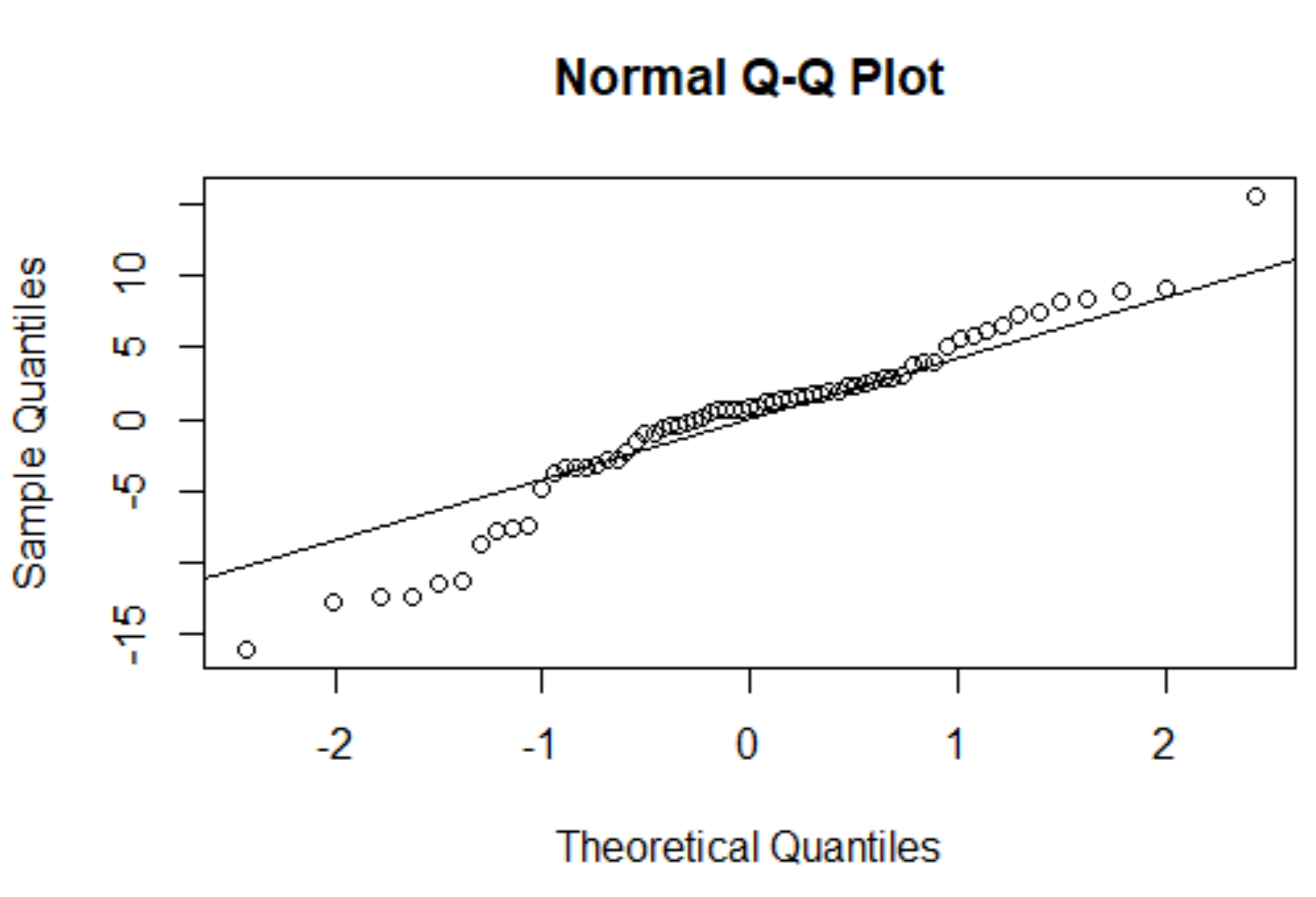

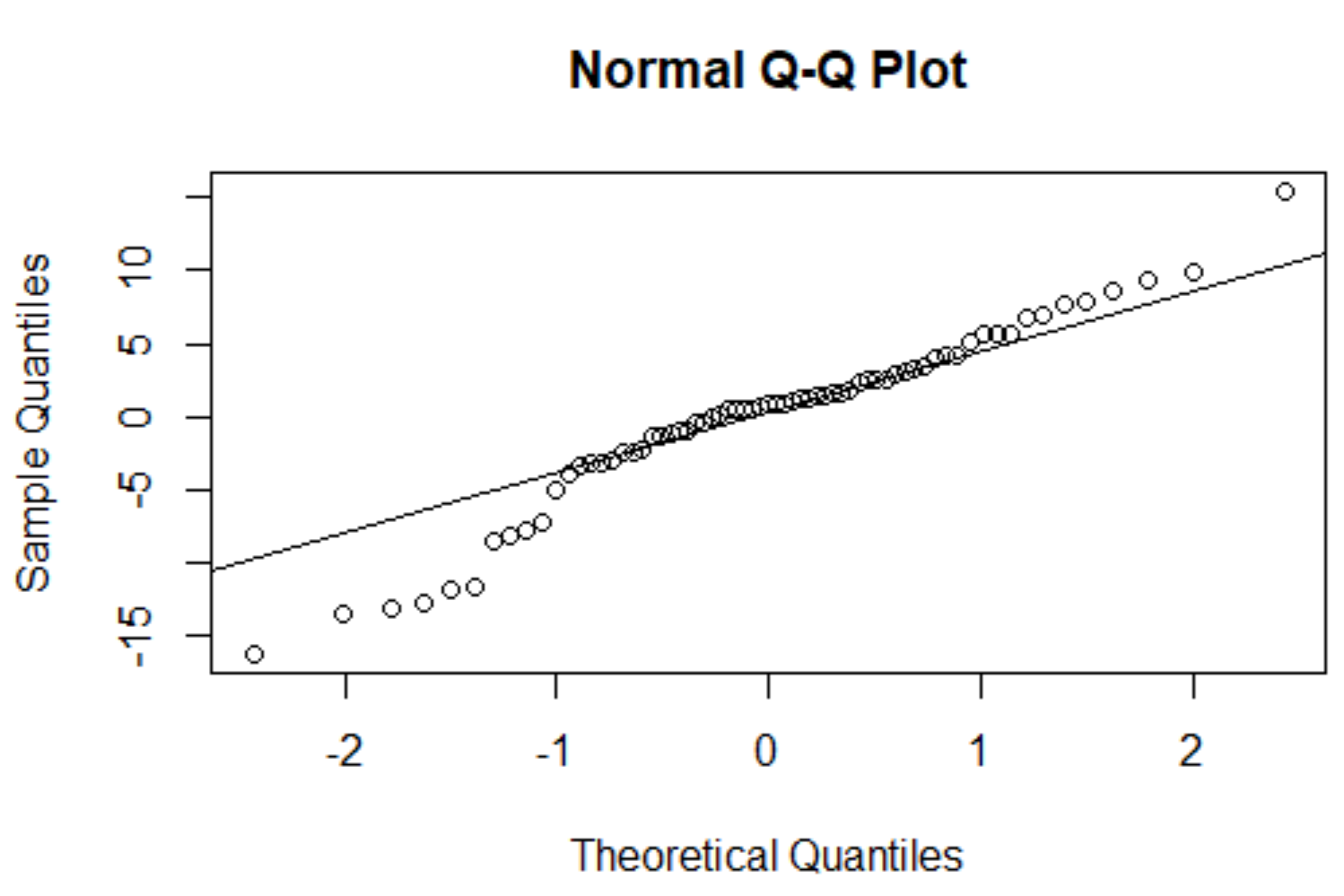


**B**
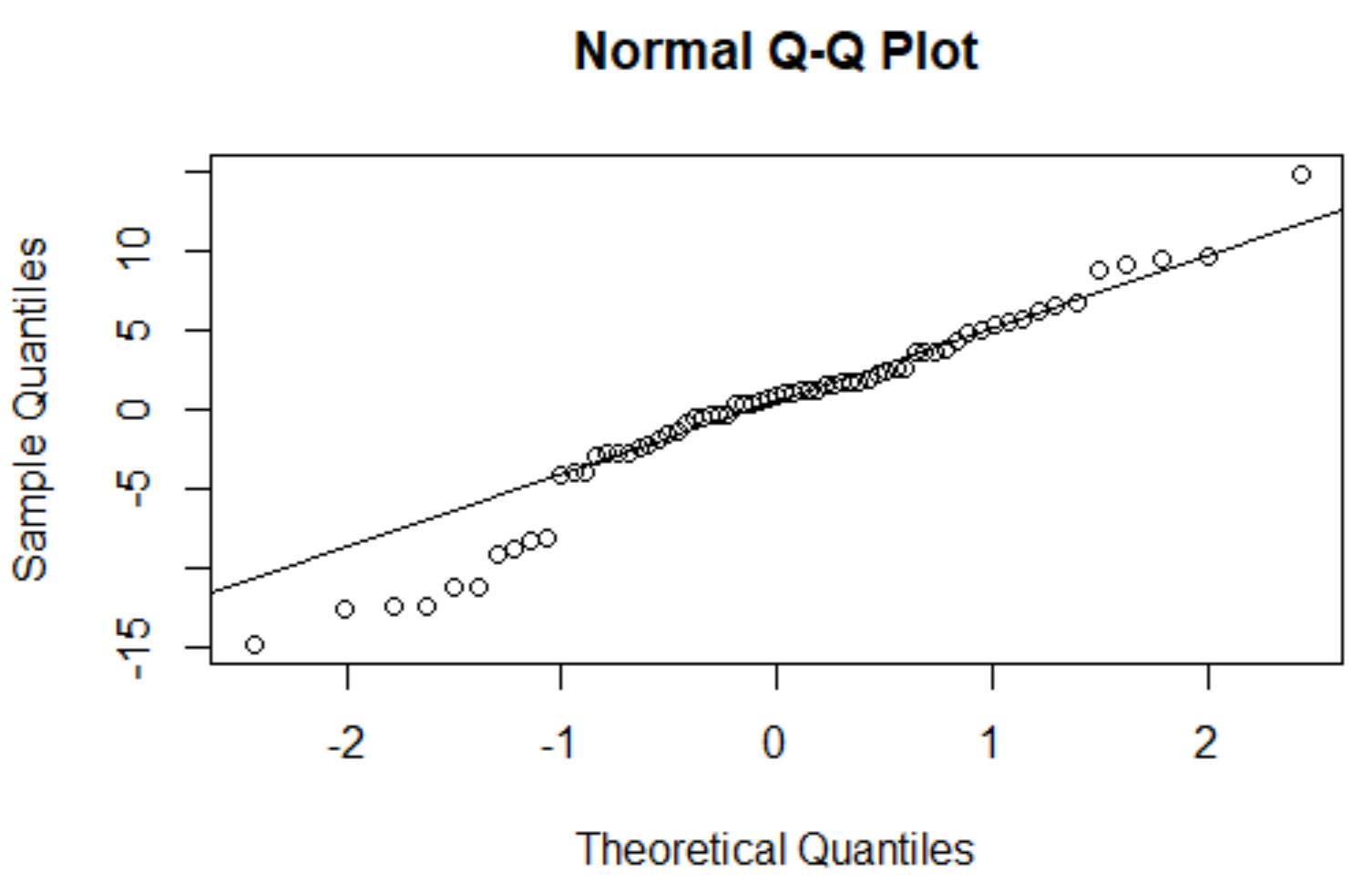


**A**
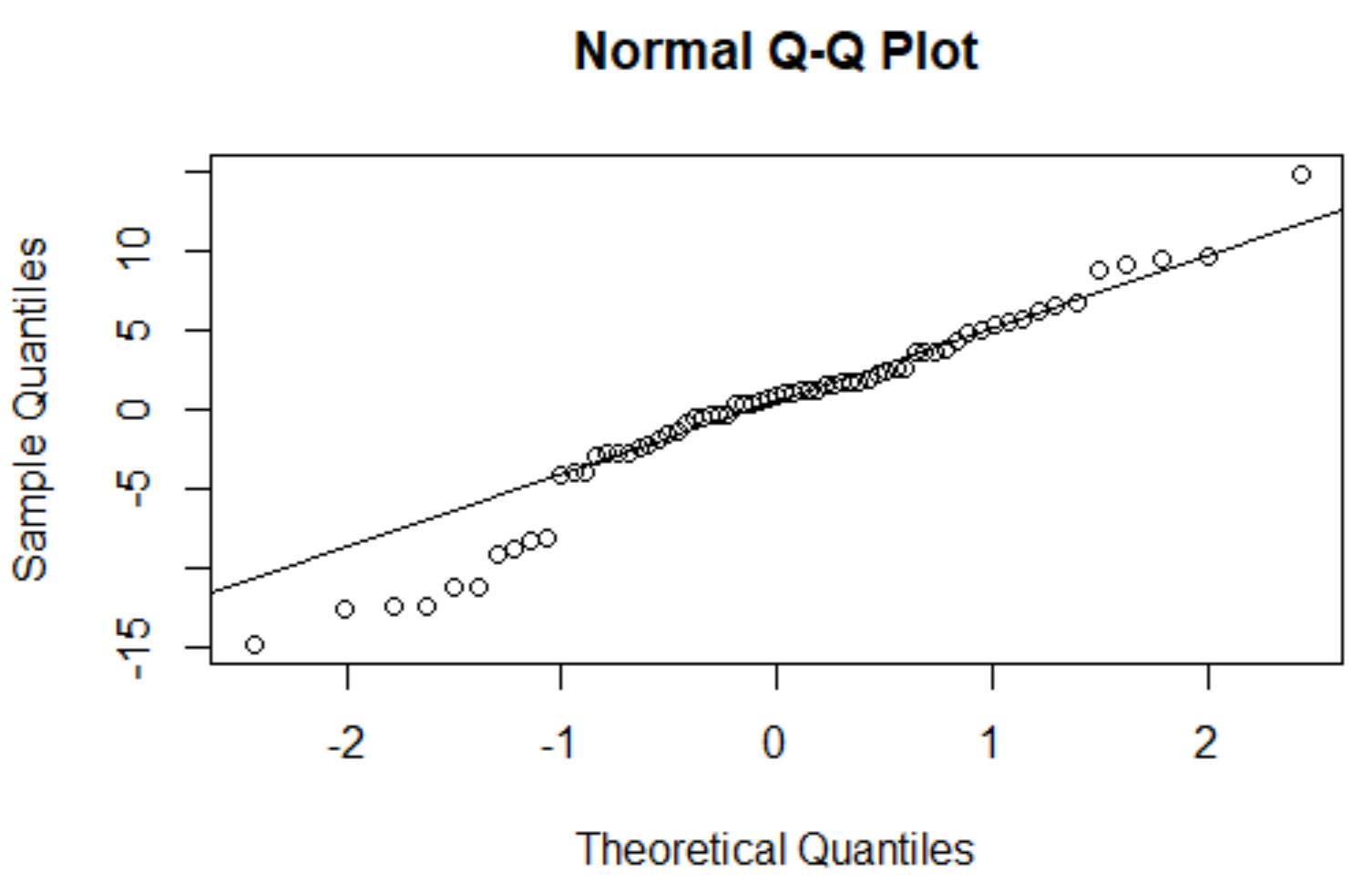


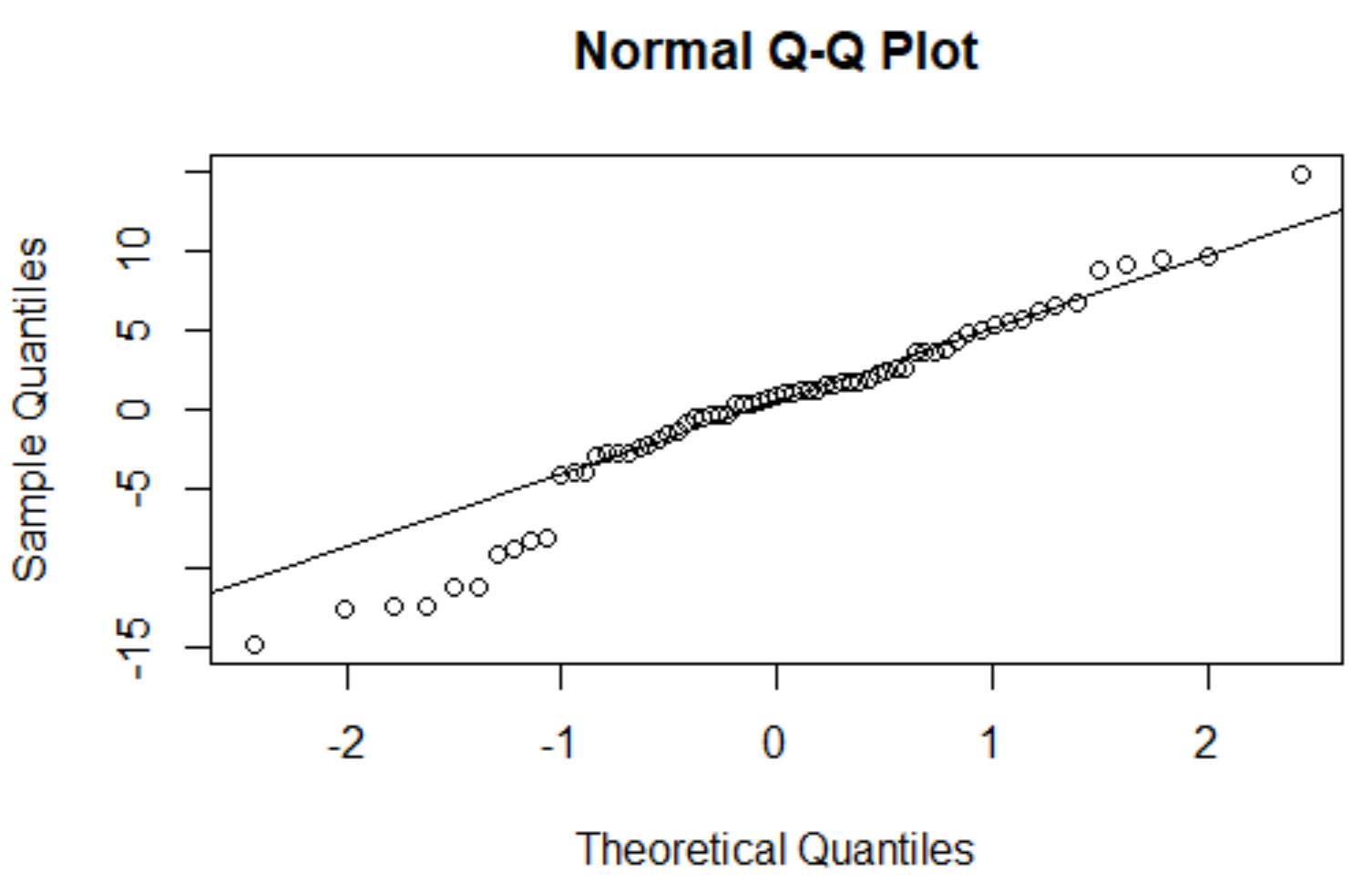


**C**
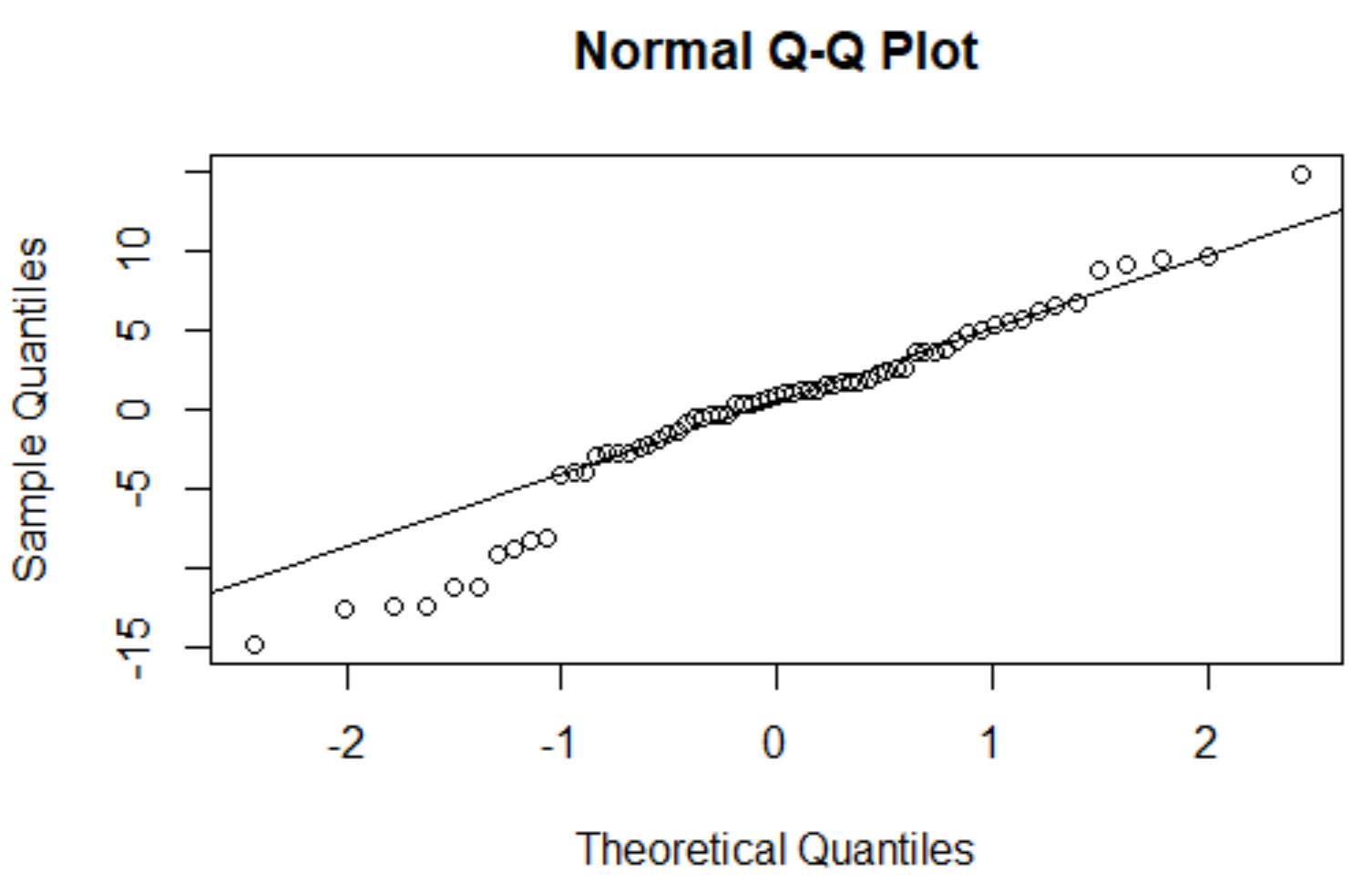


Nonnative Species Richness


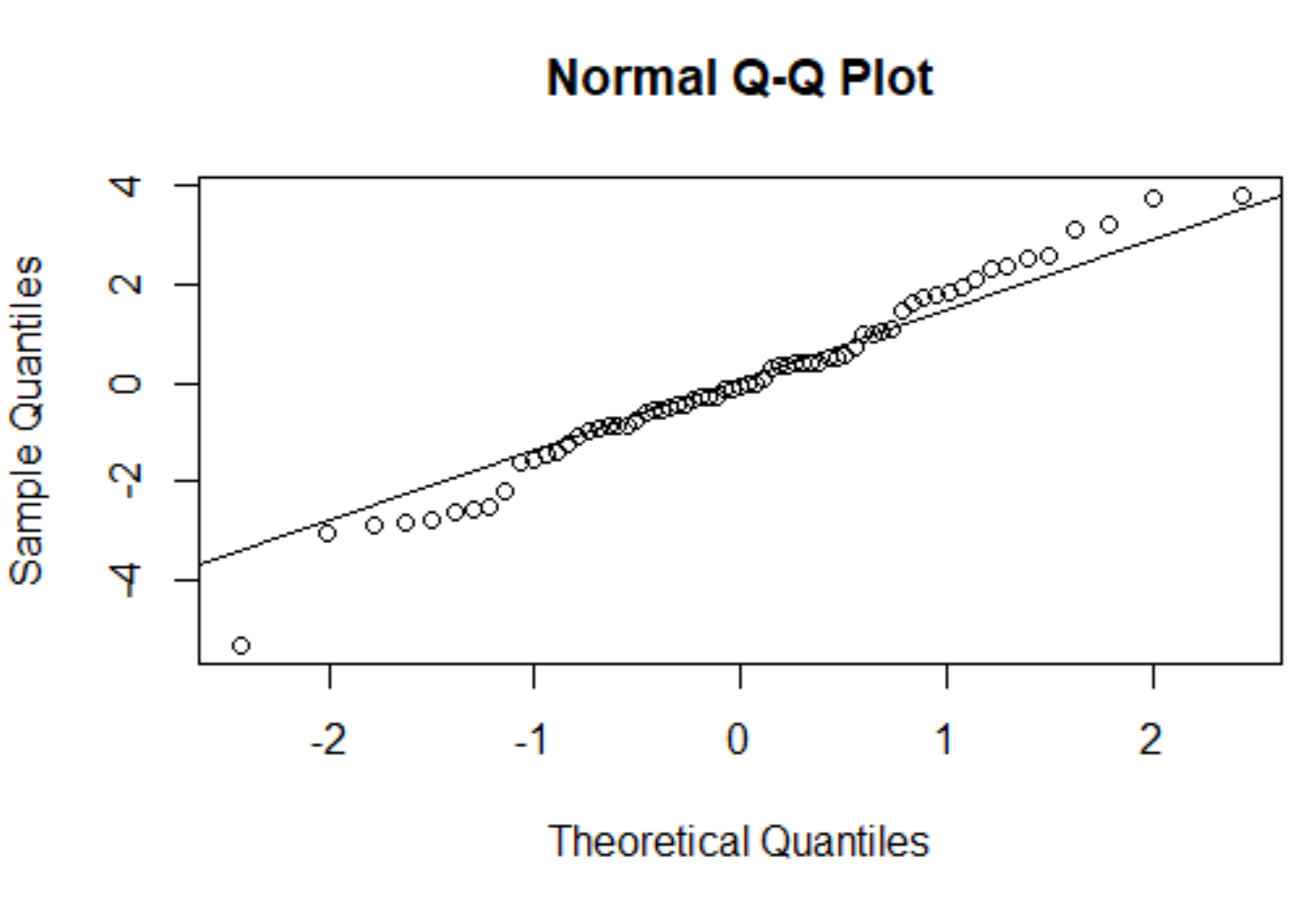

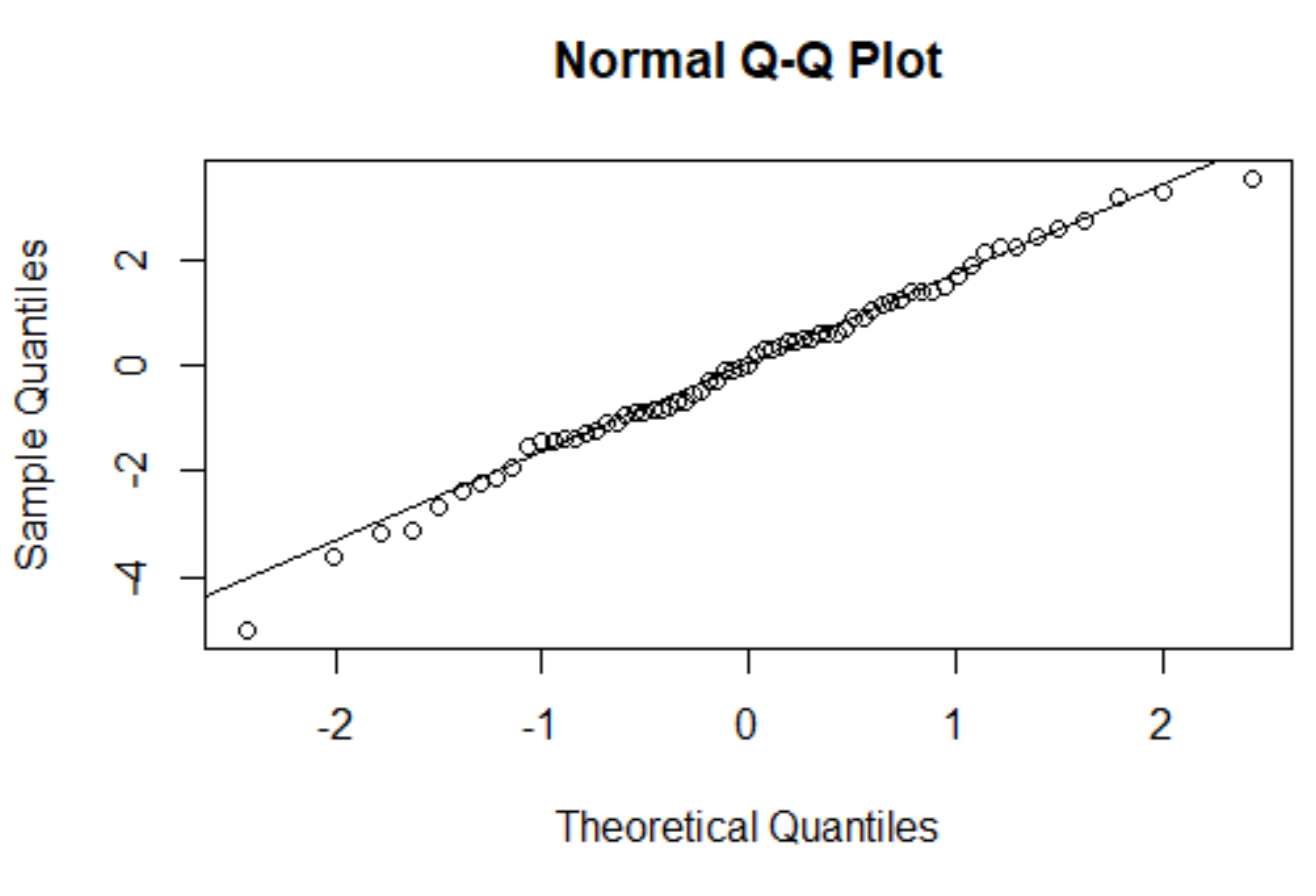


**B**
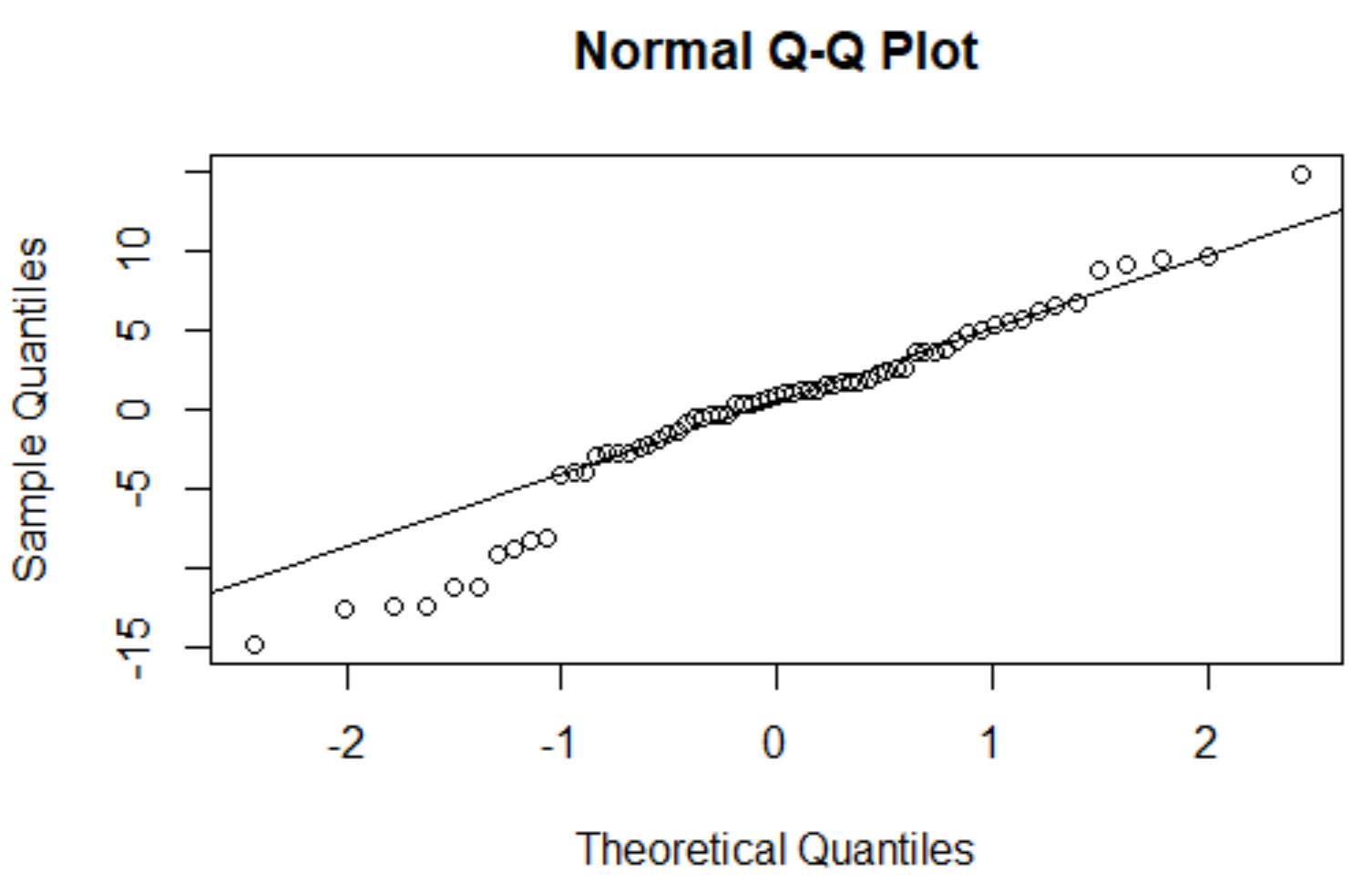


**A**
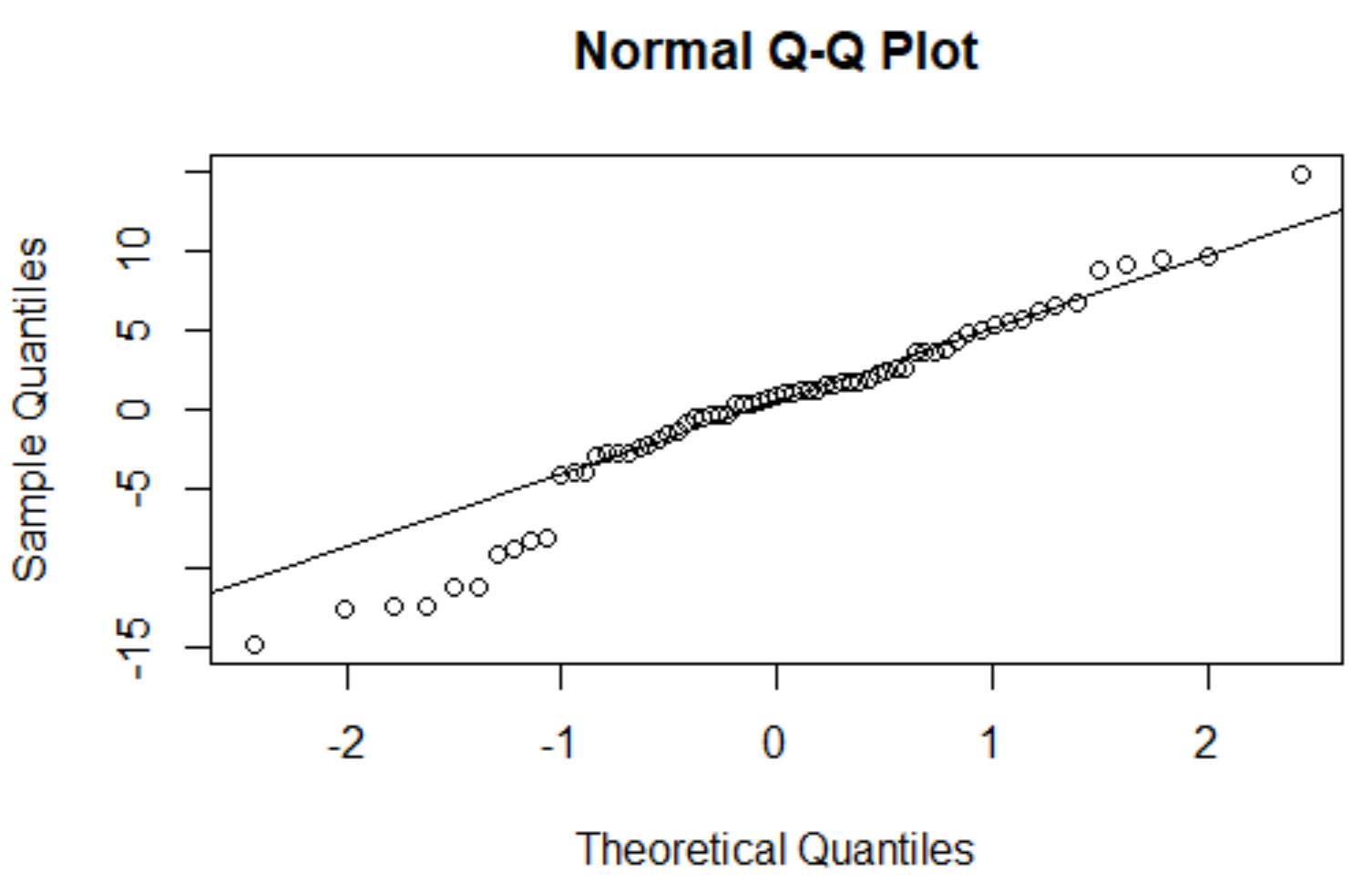


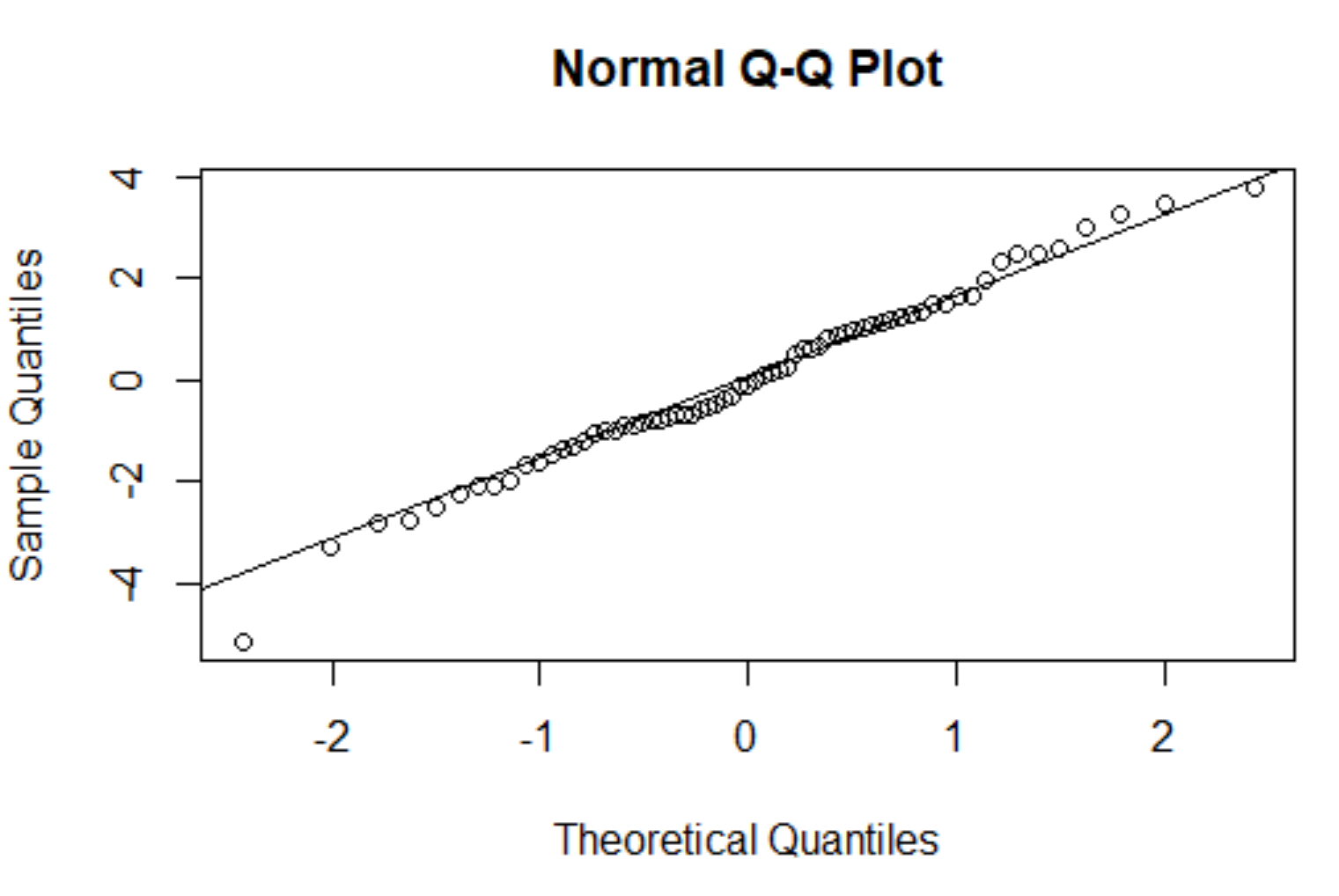


**C**
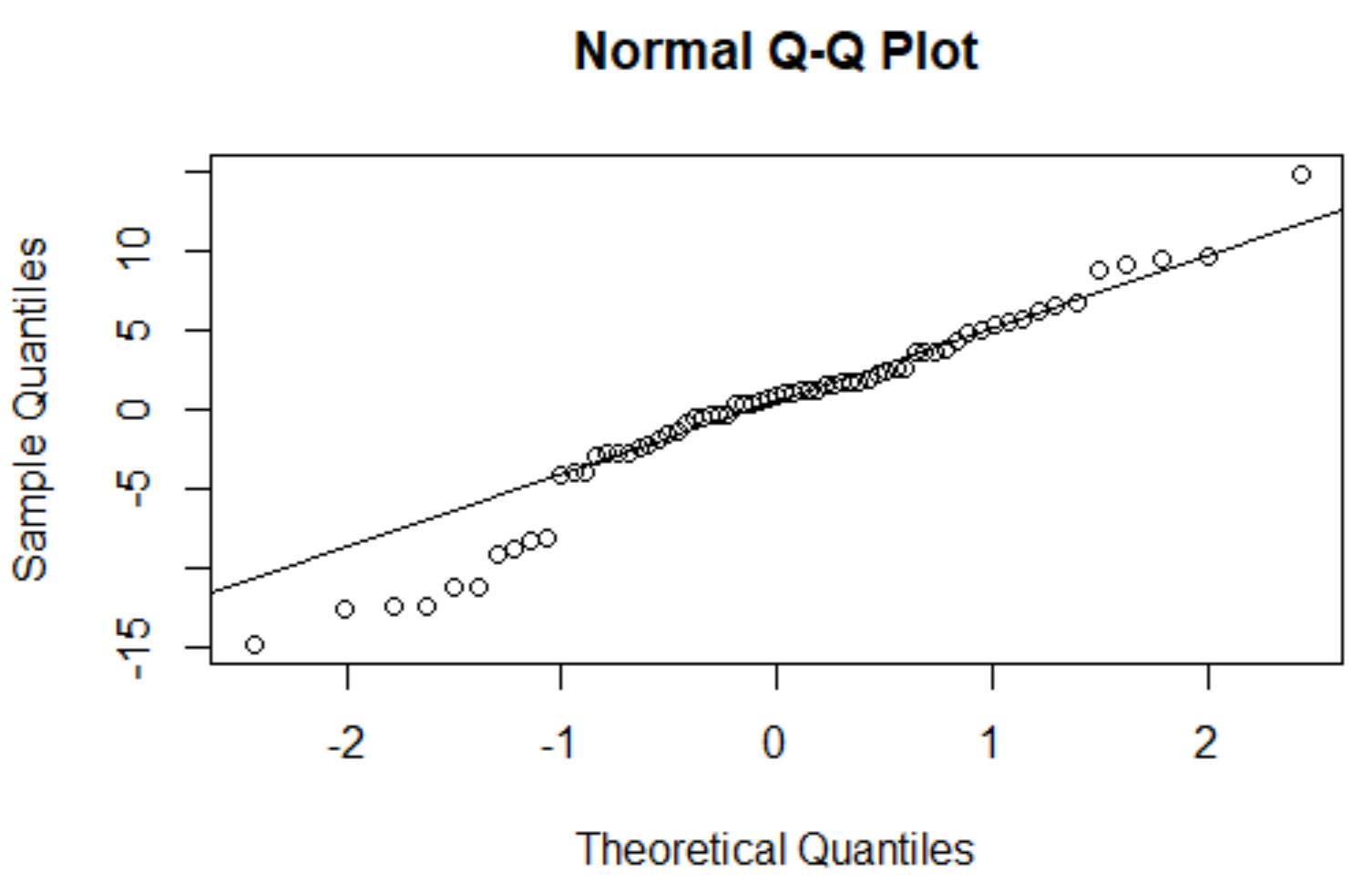

Supplement: S2 Fig — Q-Q plots for normal distribution of the residuals for the models for A. Species richness and agricultural land cover, B. Species richness and natural land cover, C. Species richness and urban land cover. (DOCX) [file pone.0327293.s002.docx]
