# Supplementary material for: Land cover as a driver of fish community changes in New York’s Oswego River Watershed
Source: PLoS One. 2025 Jul 14;20(7):e0327293. doi: 10.1371/journal.pone.0327293 (PMC12258583; doi:10.1371/journal.pone.0327293)

**S3 Figure. Centrarchidae timelines.** Presence timelines for members of the family Cenrarchidae over time in (A) the entire Oswego River Watershed, (B) the Cayuga sub-basin, (C) the Oneida North sub-basin, and (D) the Syracuse sub-basin.

1. Full watershed


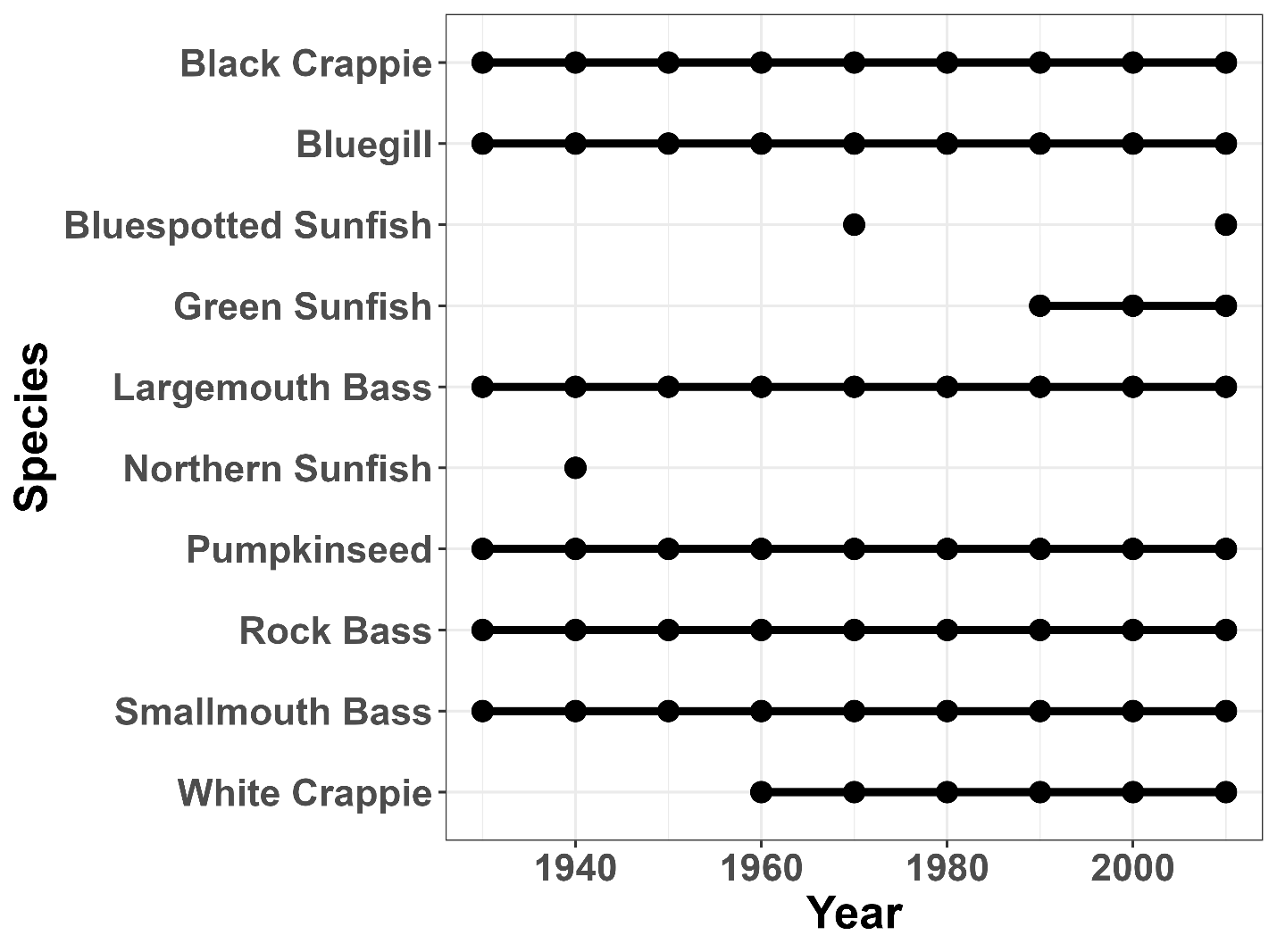


1. Cayuga sub-basin


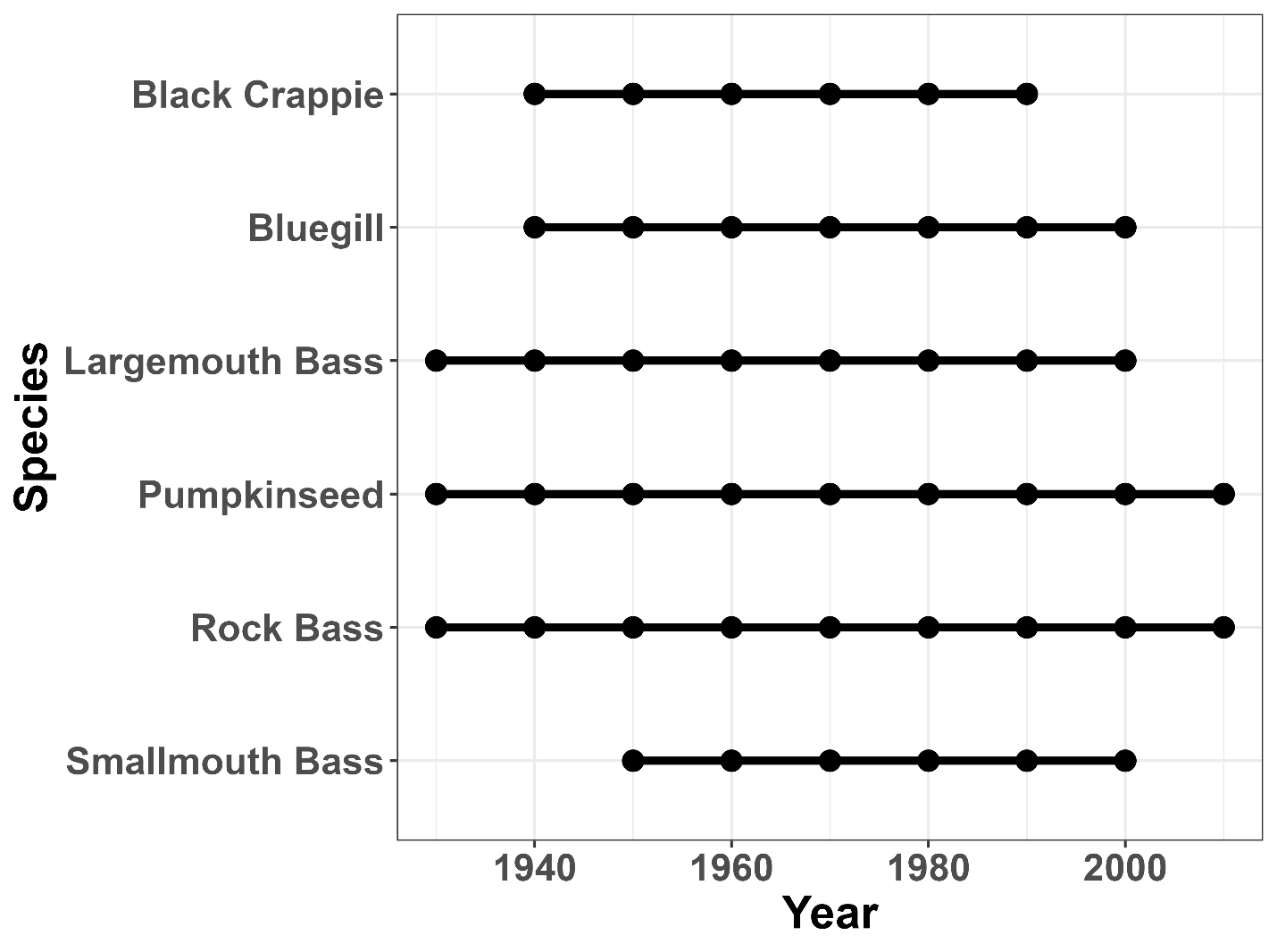


1. Oneida North sub-basin


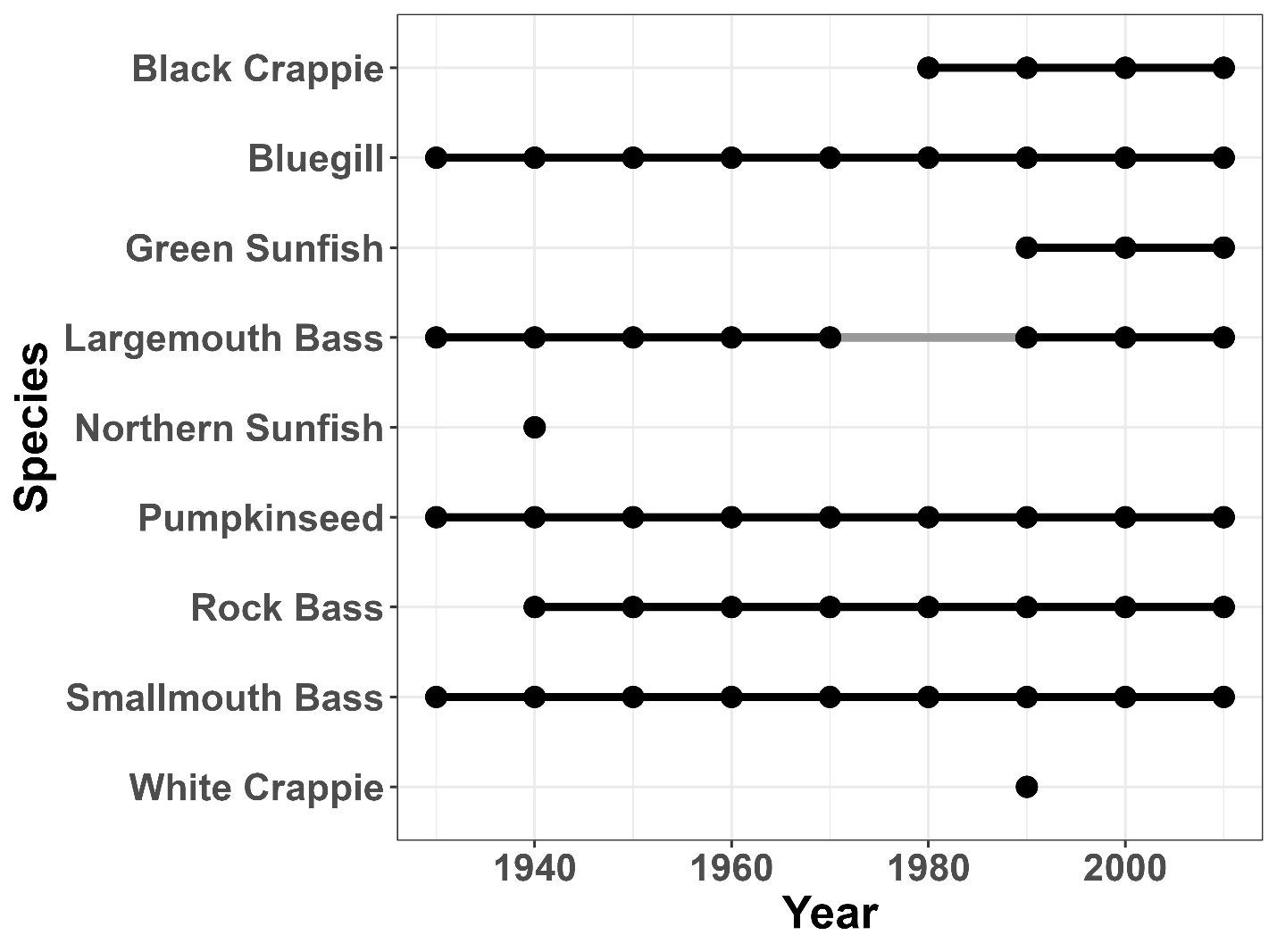


1. Syracuse sub-basin


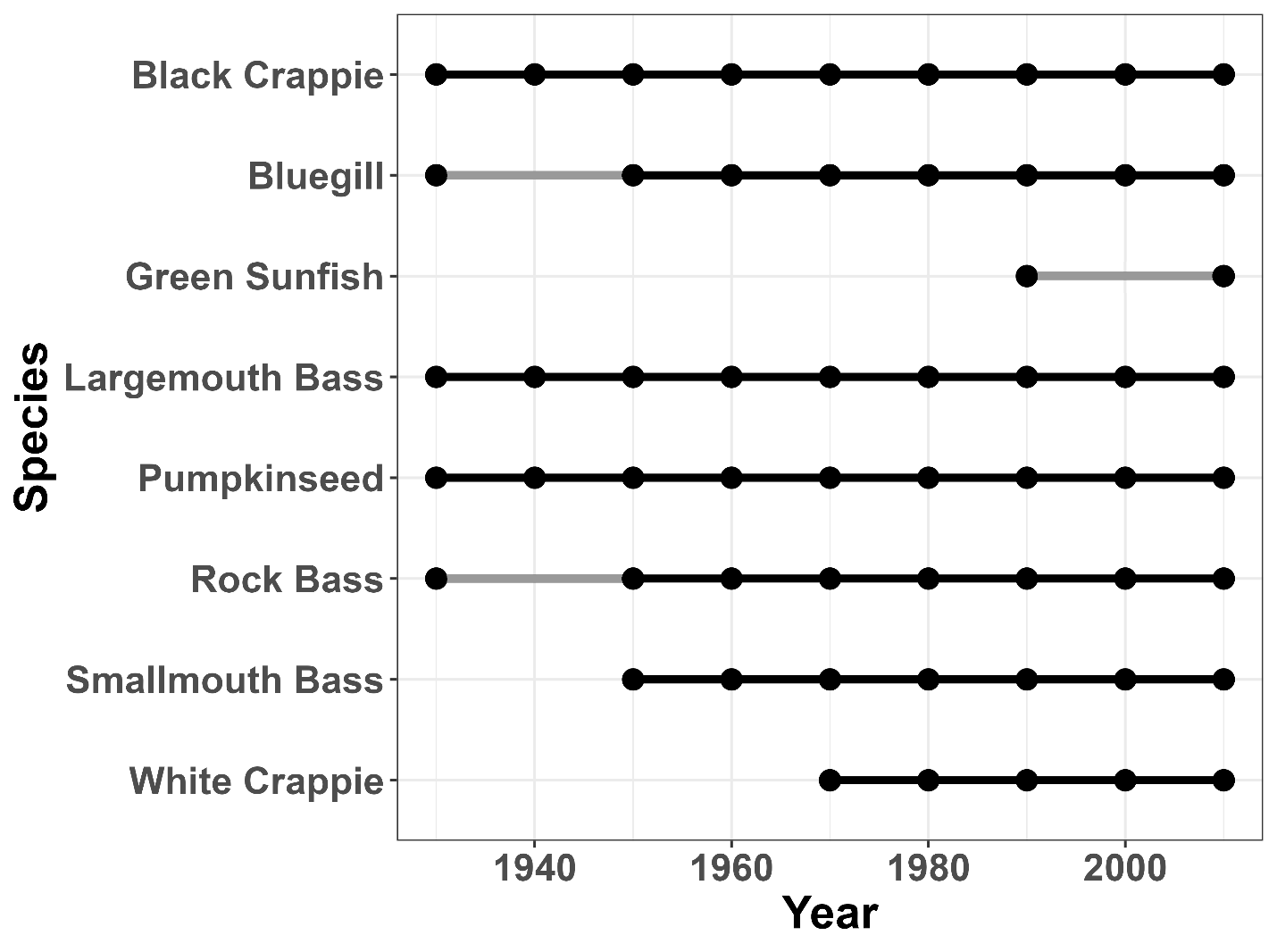

Supplement: S3 Fig — Presence timelines for members of the family Centrarchidae over time in (A) the entire Oswego River Watershed, (B) the Cayuga sub-basin, (C) the Oneida North sub-basin, and (D) the Syracuse sub-basin. (DOCX) [file pone.0327293.s003.docx]
