# Supplementary material for: Land cover as a driver of fish community changes in New York’s Oswego River Watershed
Source: PLoS One. 2025 Jul 14;20(7):e0327293. doi: 10.1371/journal.pone.0327293 (PMC12258583; doi:10.1371/journal.pone.0327293)
Supplement: S1 Table — Sediment tolerance from Trebitz et al. (2007) (marked with 1) and Whittier and Hughes (1998) (marked with 2), critical temperatures from Hazlett (2021) (marked with *), and species origins from the USGS Nonindigenous Aquatic Species List (marked with 3) and the Atlas of the Inland Fishes of New York (marked with 4). (DOCX) [file pone.0327293.s004.docx]

**S1 Table. Species classifications for analysis**. Sediment tolerance from Trebitz et al. (2007) (marked with 1) and Whittier and Hughes (1998) (marked with 2), critical temperatures from Hazlett (2021) (marked with *), and species origins from the USGS Nonindigenous Aquatic Species List (marked with 3) and the *Atlas of the Inland Fishes of New York* (marked with 4).

| **Common Name** | **Scientific Name** | **Sediment Tolerance** | **Critical Temperature (degrees C)** | **Critical Temperature Threshold** | **Origin** | **Origin Source** |
| --- | --- | --- | --- | --- | --- | --- |
| Lake Sturgeon | *Acipenser fulvescens* | NA | 35* | Higher | Native^3^ | USGS |
| Blueback Herring | *Alosa aestivalis* | NA | 27* | Lower | Nonnative^4^ | DEC |
| Alewife | *Alosa pseudoharengus* | Tolerant^1^ | 32* | Higher | Nonnative^3^ | USGS |
| Rock Bass | *Ambloplites rupestris* | Tolerant^1^ | 33* | Higher | Native^3^ | USGS |
| Black Bullhead | *Ameiurus melas* | Tolerant^1^ | 36* | Higher | Native^4^ | DEC |
| Yellow Bullhead | *Ameiurus natalis* | Tolerant^1,2^ | 36* | Higher | Native^3^ | USGS |
| Brown Bullhead | *Ameiurus nebulosus* | Tolerant^1,2^ | 38* | Higher | Native^3^ | USGS |
| Bowfin | *Amia calva* | Tolerant^1^ | 35* | Higher | Native^3^ | USGS |
| American Eel | *Anguilla rostrata* | Tolerant^2^ | 25* | Lower | Native^3^ | USGS |
| Freshwater Drum | *Aplodinotus grunniens* | Tolerant^1^ | 26* | Lower | Native^3^ | USGS |
| Central Stoneroller | *Campostoma anomalum* | NA | 31* | Lower | Native^3^ | USGS |
| Goldfish | *Carassius auratus* | Tolerant^1^ | 41* | Higher | Nonnative^3^ | USGS |
| Quillback | *Carpiodes cyprinus* | Tolerant^1^ | 39* | Higher | Native^4^ | DEC |
| Longnose Sucker | *Catostomus catostomus* | Intolerant^2^ | 27* | Lower | Native^4^ | DEC |
| White Sucker | *Catostomus commersonii* | Tolerant^1,2^ | 32* | Higher | Native^4^ | DEC |
| Redside Dace | *Clinostomus elongatus* | NA | 28* | Lower | Native^3^ | USGS |
| Cisco | *Coregonus artedi* | NA | 26* | Lower | Native^3^ | USGS |
| Lake Whitefish | *Coregonus clupeaformis* | NA | 26* | Lower | Native^3^ | USGS |
| Mottled Sculpin | *Cottus bairdii* | NA | 31* | Lower | Native^3^ | USGS |
| Slimy Sculpin | *Cottus cognatus* | Intolerant^2^ | 31* | Lower | Native^4^ | DEC |
| Lake Chub | *Couesius plumbeus* | Intolerant^2^ | 32* | Higher | Native^3^ | USGS |
| Brook Stickleback | *Culaea inconstans* | Intolerant^1^ | 31* | Lower | Native^3^ | USGS |
| Satinfin Shiner | *Cyprinella analostana* | NA | 37* | Higher | Nonnative^3^ | USGS |
| Spotfin Shiner | *Cyprinella spiloptera* | Tolerant^1^ | 33* | Higher | Native^3^ | USGS |
| Common Carp | *Cyprinus carpio* | Tolerant^1,2^ | 35* | Higher | Nonnative^3^ | USGS |
| Gizzard Shad | *Dorosoma cepedianum* | Tolerant^1^ | 34* | Higher | Native^4^ | DEC |
| Bluespotted Sunfish | *Enneacanthus gloriosus* | NA | 36* | Higher | Nonnative^4^ | DEC |
| Eastern Creek Chubsucker | *Erimyzon oblongus* | NA | 32* | Higher | Native^4^ | DEC |
| Grass Pickerel | *Esox americanus vermiculatus* | Intolerant^1^ | 32* | Higher | Native^4^ | DEC |
| Northern Pike | *Esox lucius* | Tolerant^1^ | 32* | Higher | Native^4^ | DEC |
| Tiger Musky | *Esox lucius x masquinongy* | Tolerant ^(Assumed based^ *^on E. lucius and E. masquinongy^* ^tolerance, 1)^ | 32* | Higher | Nonnative^4^ | DEC |
| Chain Pickerel | *Esox niger* | Tolerant^2^ | 27* | Lower | Native^4^ | DEC |
| Greenside Darter | *Etheostoma blennioides* | NA | 31* | Lower | Native^4^ | DEC |
| Iowa Darter | *Etheostoma exile* | NA | 30* | Lower | Native^3^ | USGS |
| Fantail Darter | *Etheostoma flabellare* | NA | 31* | Lower | Native^4^ | DEC |
| Johnny Darter | *Etheostoma nigrum* | Tolerant^1^ | 31* | Lower | Native^4^ | DEC |
| Tessellated Darter | *Etheostoma olmstedi* | Intolerant^2^ | 31* | Lower | Native^4^ | DEC |
| Cutlip Minnow | *Exoglossum maxillingua* | NA | 26* | Lower | Native^4^ | DEC |
| Banded Killifish | *Fundulus diaphanus* | Tolerant^1^ | 32* | Higher | Native^4^ | DEC |
| Threespine Stickleback | *Gasterosteus aculeatus* | Intolerant^1^ | 31* | Lower | Native^4^ | DEC |
| Mooneye | *Hiodon tergisus* | NA | 35* | Higher | Native^3^ | USGS |
| Brassy Minnow | *Hybognathus hankinsoni* | NA | 29* | Lower | Native^4^ | DEC |
| Eastern Silvery Minnow | *Hybognathus regius* | NA | 30* | Lower | Native^4^ | DEC |
| Bigeye Chub | *Hybopsis amblops* | NA | 34* | Higher | Native^4^ | DEC |
| Northern Hog Sucker | *Hypentelium nigricans* | NA | 31* | Lower | Native^4^ | DEC |
| Channel Catfish | *Ictalurus punctatus* | Tolerant^1^ | 39* | Higher | Native^4^ | DEC |
| Brook Silverside | *Labidesthes sicculus* | Tolerant^1^ | 36* | Higher | Native^4^ | DEC |
| American Brook Lamprey | *Lampetra appendix* | NA | 23* | Lower | Native^4^ | DEC |
| Longnose Gar | *Lepisosteus osseus* | Tolerant^1^ | 31* | Lower | Native^4^ | DEC |
| Green Sunfish | *Lepomis cyanellus* | Tolerant^1^ | 31* | Lower | Nonnative^4^ | DEC |
| Pumpkinseed | *Lepomis gibbosus* | Tolerant^1,2^ | 31* | Lower | Native^4^ | DEC |
| Bluegill | *Lepomis macrochirus* | Tolerant^1,2^ | 36* | Higher | Native^4^ | DEC |
| Northern Sunfish | *Lepomis peltastes* | NA | 38* | Higher | Native^4^ | DEC |
| Burbot | *Lota lota* | Intolerant^2^ | 18* | Lower | Native^4^ | DEC |
| Striped Shiner | *Luxilus chrysocephalus* | NA | 31* | Lower | Native^4^ | DEC |
| Common Shiner | *Luxilus cornutus* | Tolerant^1^ | 31* | Lower | Native^4^ | DEC |
| Redfin Shiner | *Lythrurus umbratilis* | NA | 37* | Higher | Native^3^ | USGS |
| Northern Pearl Dace | *Margaricus nachtriebi* | NA | 32* | Higher | Native^3^ | USGS |
| Allegheny Pearl Dace | *Margariscus margarita* | NA | 32* | Higher | Native^3^ | USGS |
| Smallmouth Bass | *Micropterus dolomieu* | Tolerant^1^ | 37* | Higher | Native^3^ | USGS |
| Largemouth Bass | *Micropterus salmoides* | Tolerant^1,2^ | 37* | Higher | Nonnative^4^ | DEC |
| White Perch | *Morone americana* | Tolerant^1,2^ | 36* | Higher | Nonnative^4^ | DEC |
| White Bass | *Morone chrysops* | Tolerant^1^ | 34* | Higher | Native^4^ | DEC |
| Silver Redhorse | *Moxostoma anisurum* | Intolerant^1^ | 32* | Higher | Native^4^ | DEC |
| Golden Redhorse | *Moxostoma erythrurum* | Tolerant^1^ | 32* | Higher | Native^4^ | DEC |
| Shorthead Redhorse | *Moxostoma macrolepidotum* | Intolerant^1^ | 32* | Higher | Native^4^ | DEC |
| Greater Redhorse | *Moxostoma valenciennesi* | NA | 32* | Higher | Native^4^ | DEC |
| Round Goby | *Neogobius melanostomus* | Tolerant^1^ | 30* | Lower | Nonnative^4^ | DEC |
| Hornyhead Chub | *Nocomis biguttatus* | Intolerant^1^ | 31* | Lower | Native^4^ | DEC |
| River Chub | *Nocomis micropogon* | NA | 31* | Lower | Nonnative^4^ | DEC |
| Golden Shiner | *Notemigonus crysoleucas* | Tolerant^1,2^ | 31* | Lower | Native^4^ | DEC |
| Comely Shiner | *Notropis amoenus* | NA | 33* | Higher | Nonnative^4^ | DEC |
| Pugnose Shiner | *Notropis anogenus* | NA | 33* | Higher | Native^4^ | DEC |
| Emerald Shiner | *Notropis atherinoides* | Tolerant^1^ | 33* | Higher | Native^4^ | DEC |
| Bridle Shiner | *Notropis bifrenatus* | Intolerant^2^ | 33* | Higher | Native^4^ | DEC |
| Bigmouth Shiner | *Notropis dorsalis* | NA | 33* | Higher | Native^4^ | DEC |
| Blackchin Shiner | *Notropis heterodon* | Intolerant^1^ | 33* | Higher | Native^4^ | DEC |
| Blacknose Shiner | *Notropis heterolepis* | Intolerant^1,2^ | 33* | Higher | Native^4^ | DEC |
| Spottail Shiner | *Notropis hudsonius* | Tolerant^1^ | 33* | Higher | Native^4^ | DEC |
| Swallowtail Shiner | *Notropis procne* | NA | 33* | Higher | Nonnative^4^ | DEC |
| Rosyface Shiner | *Notropis rubellus* | NA | 33* | Higher | Native^4^ | DEC |
| Sand Shiner | *Notropis stramineus* | Tolerant^1^ | 33* | Higher | Native^4^ | DEC |
| Mimic Shiner | *Notropis volucellus* | Intolerant^1^ | 33* | Higher | Native^4^ | DEC |
| Stonecat | *Noturus flavus* | NA | 29* | Lower | Native^4^ | DEC |
| Tadpole Madtom | *Noturus gyrinus* | Intolerant^1^ | 38* | Higher | Native^4^ | DEC |
| Margined Madtom | *Noturus insignis* | NA | 33* | Higher | Nonnative^4^ | DEC |
| Brindled Madtom | *Noturus miurus* | NA | 33* | Higher | Native^4^ | DEC |
| Coho Salmon | *Oncorhynchus kisutch* | NA | 26* | Lower | Nonnative^4^ | DEC |
| Rainbow Trout | *Oncorhynchus mykiss* | NA | 25* | Lower | Nonnative^4^ | DEC |
| Sockeye Salmon | *Oncorhynchus nerka* | NA | 24* | Lower | Nonnative^4^ | DEC |
| Chinook Salmon | *Oncorhynchus tshawytscha* | NA | 26* | Lower | Nonnative^4^ | DEC |
| Rainbow Smelt | *Osmerus mordax* | Intolerant^2^ | 26* | Lower | Nonnative^4^ | DEC |
| Yellow Perch | *Perca flavescens* | Tolerant^1,2^ | 33* | Higher | Native^4^ | DEC |
| Logperch | *Percina caprodes* | Tolerant^1^ | 35* | Higher | Native^3^ | USGS |
| Blackside Darter | *Percina maculata* | NA | 35* | Higher | Native^4^ | DEC |
| Trout-Perch | *Percopsis omiscomaycus* | Intolerant^1^ | 23* | Lower | Native^4^ | DEC |
| Sea Lamprey | *Petromyzon marinus* | NA | 30* | Lower | Nonnative^3^ | USGS |
| Northern Redbelly Dace | *Phoxinus eos* | Intolerant^2^ | 29* | Lower | Native^3^ | USGS |
| Bluntnose Minnow | *Pimephales notatus* | Tolerant^1^ | 32* | Higher | Native^4^ | DEC |
| Fathead Minnow | *Pimephales promelas* | Tolerant^1^ | 35* | Higher | Native^4^ | DEC |
| White Crappie | *Pomoxis annularis* | NA | 31* | Lower | Nonnative^4^ | DEC |
| Black Crappie | *Pomoxis nigromaculatus* | Tolerant^1,2^ | 31* | Lower | Nonnative^4^ | DEC |
| Ninespine Stickleback | *Pungitius pungitius* | NA | 31* | Lower | Native^3^ | USGS |
| Eastern Blacknose Dace | *Rhinichthys atratulus* | NA | 30* | Lower | Native^4^ | DEC |
| Longnose Dace | *Rhinichthys cataractae* | Tolerant^1^ | 28* | Lower | Native^4^ | DEC |
| Atlantic Salmon | *Salmo salar* | Intolerant^2^ | 28* | Lower | Native^4^ | DEC |
| Brown Trout | *Salmo trutta* | NA | 28* | Lower | Nonnative^4^ | DEC |
| Brook Trout | *Salvelinus fontinalis* | Intolerant^2^ | 24* | Lower | Native^4^ | DEC |
| Lake Trout | *Salvelinus namaycush* | Intolerant^2^ | 21* | Lower | Native^4^ | DEC |
| Sauger | *Sander canadensis* | NA | 31* | Lower | Native^4^ | DEC |
| Walleye | *Sander vitreus* | Tolerant^1^ | 30* | Lower | Native^3^ | USGS |
| Blue Pike | *Sander vitreus glaucus* | NA | 30* | Lower | Native^4^ | DEC |
| Rudd | *Scardinius erythrophthalmus* | NA | 22* | Lower | Nonnative^4^ | DEC |
| Creek Chub | *Semotilus atromaculatus* | Intolerant^1,2^ | 32* | Higher | Native^4^ | DEC |
| Fallfish | *Semotilus corporalis* | Intolerant^2^ | 27* | Lower | Native^4^ | DEC |
| Central Mudminnow | *Umbra limi* | Intolerant^1^ | 29* | Lower | Native^4^ | DEC |
