# Supplementary material for: Land cover as a driver of fish community changes in New York’s Oswego River Watershed
Source: PLoS One. 2025 Jul 14;20(7):e0327293. doi: 10.1371/journal.pone.0327293 (PMC12258583; doi:10.1371/journal.pone.0327293)
Supplement: S2 Table — The number of fish counted in surveys in each sub-basin of the watershed during each decade, and the species richness from that count. Combinations of sub-basin and decade with fewer than 50 observations were excluded from the analysis for a lack of data, since these species counts are likely artificially low. (DOCX) [file pone.0327293.s005.docx]

**S2 Table. Fish observations and species richness by site and decade.** The number of fish counted in surveys in each sub-basin of the watershed during each decade, and the species richness from that count. Combinations of sub-basin and decade with fewer than 50 observations were excluded from the analysis for a lack of data, since these species counts are likely artificially low.

| **Sub-Basin** | **Decade** | **Observations** | **Species** |
| --- | --- | --- | --- |
| Canandaigua | 1930 | 56 | 15 |
| Canandaigua | 1940 | 216 | 29 |
| Canandaigua | 1950 | 185 | 29 |
| Canandaigua | 1960 | 450 | 37 |
| Canandaigua | 1970 | 302 | 37 |
| Canandaigua | 1980 | 74 | 31 |
| Canandaigua | 1990 | 38 | 12 |
| Canandaigua | 2000 | 302 | 22 |
| Canandaigua | 2010 | 48 | 12 |
| Cayuga | 1930 | 156 | 34 |
| Cayuga | 1940 | 79 | 32 |
| Cayuga | 1950 | 715 | 47 |
| Cayuga | 1960 | 327 | 53 |
| Cayuga | 1970 | 1035 | 48 |
| Cayuga | 1980 | 4668 | 43 |
| Cayuga | 1990 | 2831 | 55 |
| Cayuga | 2000 | 1384 | 47 |
| Cayuga | 2010 | 201 | 23 |
| Keuka | 1930 | 96 | 25 |
| Keuka | 1940 | 67 | 27 |
| Keuka | 1950 | 190 | 32 |
| Keuka | 1960 | 467 | 37 |
| Keuka | 1970 | 937 | 50 |
| Keuka | 1980 | 283 | 38 |
| Keuka | 1990 | 127 | 28 |
| Keuka | 2000 | 734 | 44 |
| Keuka | 2010 | 274 | 32 |
| OneidaN | 1930 | 122 | 27 |
| OneidaN | 1940 | 151 | 43 |
| OneidaN | 1950 | 898 | 39 |
| OneidaN | 1960 | 555 | 36 |
| OneidaN | 1970 | 926 | 41 |
| OneidaN | 1980 | 351 | 48 |
| OneidaN | 1990 | 1216 | 60 |
| OneidaN | 2000 | 571 | 52 |
| OneidaN | 2010 | 249 | 43 |
| OneidaS | 1930 | 81 | 21 |
| OneidaS | 1940 | 359 | 41 |
| OneidaS | 1950 | 294 | 32 |
| OneidaS | 1960 | 412 | 37 |
| OneidaS | 1970 | 702 | 44 |
| OneidaS | 1980 | 99 | 22 |
| OneidaS | 1990 | 830 | 55 |
| OneidaS | 2000 | 621 | 52 |
| OneidaS | 2010 | 134 | 36 |
| Seneca | 1930 | 419 | 51 |
| Seneca | 1940 | 132 | 38 |
| Seneca | 1950 | 341 | 42 |
| Seneca | 1960 | 183 | 31 |
| Seneca | 1970 | 503 | 52 |
| Seneca | 1980 | 451 | 56 |
| Seneca | 1990 | 162 | 37 |
| Seneca | 2000 | 570 | 56 |
| Seneca | 2010 | 37 | 27 |
| Skaneateles | 1930 | 48 | 17 |
| Skaneateles | 1940 | 195 | 29 |
| Skaneateles | 1950 | 58 | 14 |
| Skaneateles | 1960 | 652 | 30 |
| Skaneateles | 1970 | 643 | 34 |
| Skaneateles | 1980 | 913 | 33 |
| Skaneateles | 1990 | 645 | 34 |
| Skaneateles | 2000 | 409 | 34 |
| Skaneateles | 2010 | 4 | 4 |
| Syracuse | 1930 | 305 | 39 |
| Syracuse | 1940 | 61 | 25 |
| Syracuse | 1950 | 322 | 44 |
| Syracuse | 1960 | 399 | 52 |
| Syracuse | 1970 | 1644 | 59 |
| Syracuse | 1980 | 1207 | 53 |
| Syracuse | 1990 | 1334 | 65 |
| Syracuse | 2000 | 874 | 57 |
| Syracuse | 2010 | 248 | 51 |
