# Supplementary material for: Land cover as a driver of fish community changes in New York’s Oswego River Watershed
Source: PLoS One. 2025 Jul 14;20(7):e0327293. doi: 10.1371/journal.pone.0327293 (PMC12258583; doi:10.1371/journal.pone.0327293)
Supplement: S3 Table — Comparison between the results of linear mixed effects models using the full dataset (all site-decade combinations with at least 50 observations) and the reduced/rarefied dataset (only site-decade combinations with at least 250 observations, and only a random subset of 250 observations used for site-decade combinations with larger numbers of observations). (DOCX) [file pone.0327293.s006.docx]

**S3 Table. Full and reduced model comparisons** Comparison between the results of linear mixed effects models using the full dataset (all site-decade combinations with at least 50 observations) and the reduced/rarefied dataset (only site-decade combinations with at least 250 observations, and only a random subset of 250 observations used for site-decade combinations with larger numbers of observations).

|  | **Urban (Full Model)** | **Urban (Rarefied Model)** | **Agriculture (Full Model)** | **Agriculture (Rarefied Model)** | **Natural (Full Model)** | **Natural (Rarefied Model)** |
| --- | --- | --- | --- | --- | --- | --- |
| **Fixed Effect** | 1.51 | 0.72 | -0.58 | -0.19 | 0.74 | 0.21 |
| **Standard Error** | 0.52 | 0.38 | 0.15 | 0.10 | 0.19 | 0.13 |
| **ANOVA p-value** | 0.0056 | 0.09 | 0.0071 | 0.16 | 0.022 | 0.18 |
