# Supplementary material for: Land cover as a driver of fish community changes in New York’s Oswego River Watershed
Source: PLoS One. 2025 Jul 14;20(7):e0327293. doi: 10.1371/journal.pone.0327293 (PMC12258583; doi:10.1371/journal.pone.0327293)
Supplement: S4 Table — Tables depict the amount of variance explained by decade, sub-basin, and residuals for each model. (DOCX) [file pone.0327293.s007.docx]

**S4 Table. Sources of random variation in each model.** Tables depict the amount of variance explained by decade, sub-basin, and residuals for each model.

Full Species Richness

|  | **Decade Variance** | **Sub-basin Variance** | **Residual Variance** |
| --- | --- | --- | --- |
| **Urban** | 20.12 | 31.32 | 59.88 |
| **Agriculture** | 7.23 | 81.77 | 58.72 |
| **Natural** | 5.00 | 106.11 | 7.75 |

Sediment-Tolerant Species Richness

|  | **Decade Variance** | **Sub-basin Variance** | **Residual Variance** |
| --- | --- | --- | --- |
| **Urban** | 3.87 | 12.66 | 17.32 |
| **Agriculture** | 0.37 | 53.19 | 16.39 |
| **Natural** | -- | -- | -- |

Sediment-Intolerant Species Richness

|  | **Decade Variance** | **Sub-basin Variance** | **Residual Variance** |
| --- | --- | --- | --- |
| **Urban** | 0.45 | 1.78 | 5.23 |
| **Agriculture** | 0.35 | 2.19 | 5.14 |
| **Natural** | 0.38 | 2.18 | 5.11 |

High-Temperature-Tolerant Species Richness

|  | **Decade Variance** | **Sub-basin Variance** | **Residual Variance** |
| --- | --- | --- | --- |
| **Urban** | 1.63 | 12.92 | 18.90 |
| **Agriculture** | 0.33 | 35.44 | 19.08 |
| **Natural** | 1.02 | 36.98 | 19.76 |

High-Temperature-Intolerant Species Richness

|  | **Decade Variance** | **Sub-basin Variance** | **Residual Variance** |
| --- | --- | --- | --- |
| **Urban** | 9.66 | 5.33 | 17.02 |
| **Agriculture** | 6.61 | 7.56 | 16.48 |
| **Natural** | 6.60 | 7.18 | 16.67 |

Native Species Richness

|  | **Decade Variance** | **Sub-basin Variance** | **Residual Variance** |
| --- | --- | --- | --- |
| **Urban** | 9.47 | 25.43 | 41.23 |
| **Agriculture** | 3.55 | 48.39 | 40.64 |
| **Natural** | 2.28 | 55.20 | 41.36 |

Nonnative Species Richness

|  | **Decade Variance** | **Sub-basin Variance** | **Residual Variance** |
| --- | --- | --- | --- |
| **Urban** | 2.30 | 1.28 | 3.63 |
| **Agriculture** | 0.70 | 2.27 | 1.91 |
| **Natural** | 2.93 | 2.54 | 3.74 |
