# Supplementary material for: Land cover as a driver of fish community changes in New York’s Oswego River Watershed
Source: PLoS One. 2025 Jul 14;20(7):e0327293. doi: 10.1371/journal.pone.0327293 (PMC12258583; doi:10.1371/journal.pone.0327293)
Supplement: S5 Table — Tables for each sub-basin showing species added (appeared in the watershed for the first time), reappeared (present after having been absent for the previous decade), rejoined (present after having been absent for two or more decades), remained, missing (absent after being present in the previous decade), and lost (absent for two consecutive decades). (DOCX) [file pone.0327293.s008.docx]

**S5 Table. Species gains and losses over time in each sub-basin of the Oswego River Watershed.** Tables for each sub-basin showing species added (appeared in the watershed for the first time), reappeared (present after having been absent for the previous decade), rejoined (present after having been absent for two or more decades), remained, missing (absent after being present in the previous decade), and lost (absent for two consecutive decades).

Canandaigua

|  | **1930** | **1940** | **1950** | **1960** | **1970** | **1980** | **2000** |
| --- | --- | --- | --- | --- | --- | --- | --- |
| Add (First Time) | 15 | 21 | 7 | 7 | 4 | 1 | 0 |
| Reappear (Had been absent 1 period) | NA | NA | 2 | 6 | 1 | 1 | 5 |
| Rejoin (Had been absent 2+ periods) | NA | NA | NA | 1 | 4 | 1 | 2 |
| Remained | NA | 8 | 20 | 23 | 28 | 28 | 26 |
| Missing (absent 1 period) | NA | 7 | 10 | 6 | 9 | 9 | 5 |
| Lost (absent 2 periods) | NA | NA | 5 | 3 | 5 | 8 | 4 |
|  |  |  |  |  |  |  |  |
| **Total Richness** | 15 | 29 | 29 | 37 | 37 | 31 | 33 |
|  |  |  |  |  |  |  |  |

Cayuga

|  | **1930** | **1940** | **1950** | **1960** | **1970** | **1980** | **1990** | **2000** | **2010** |
| --- | --- | --- | --- | --- | --- | --- | --- | --- | --- |
| Add (First Time) | 34 | 11 | 17 | 9 | 4 | 0 | 2 | 0 | 2 |
| Reappear (Had been absent 1 period) | NA | NA | 9 | 5 | 3 | 1 | 5 | 0 | 2 |
| Rejoin (Had been absent 2+ periods) | NA | NA | NA | 1 | 2 | 0 | 7 | 3 | 0 |
| Remained | NA | 21 | 21 | 38 | 39 | 42 | 41 | 44 | 19 |
| Missing (absent 1 period) | NA | 13 | 11 | 9 | 14 | 6 | 2 | 11 | 28 |
| Lost (absent 2 periods) | NA | NA | 4 | 6 | 6 | 13 | 1 | 2 | 9 |
|  |  |  |  |  |  |  |  |  |  |
| **Total Richness** | 34 | 32 | 47 | 53 | 48 | 43 | 55 | 47 | 23 |

Keuka

|  | **1930** | **1940** | **1950** | **1960** | **1970** | **1980** | **1990** | **2000** | **2010** |
| --- | --- | --- | --- | --- | --- | --- | --- | --- | --- |
| Add (First Time) | 25 | 14 | 9 | 6 | 8 | 2 | 2 | 1 | 3 |
| Reappear (Had been absent 1 period) | NA | NA | 5 | 2 | 4 | 2 | 2 | 8 | 0 |
| Rejoin (Had been absent 2+ periods) | NA | NA | NA | 2 | 5 | 0 | 0 | 10 | 1 |
| Remained | NA | 13 | 18 | 27 | 33 | 34 | 24 | 25 | 28 |
| Missing (absent 1 period) | NA | 12 | 9 | 5 | 4 | 16 | 14 | 3 | 16 |
| Lost (absent 2 periods) | NA | NA | 7 | 7 | 1 | 2 | 14 | 6 | 3 |
|  |  |  |  |  |  |  |  |  |  |
| **Total Richness** | 25 | 27 | 32 | 37 | 50 | 38 | 28 | 44 | 32 |

Oneida North

|  | **1930** | **1940** | **1950** | **1960** | **1970** | **1980** | **1990** | **2000** | **2010** |
| --- | --- | --- | --- | --- | --- | --- | --- | --- | --- |
| Add (First Time) | 27 | 24 | 6 | 1 | 2 | 7 | 8 | 3 | 0 |
| Reappear (Had been absent 1 period) | NA | NA | 4 | 1 | 4 | 0 | 5 | 0 | 4 |
| Rejoin (Had been absent 2+ periods) | NA | NA | NA | 1 | 2 | 5 | 4 | 1 | 2 |
| Remained | NA | 19 | 29 | 33 | 33 | 36 | 43 | 48 | 37 |
| Missing (absent 1 period) | NA | 8 | 14 | 6 | 3 | 5 | 5 | 12 | 15 |
| Lost (absent 2 periods) | NA | NA | 4 | 13 | 2 | 3 | 0 | 5 | 8 |
|  |  |  |  |  |  |  |  |  |  |
| **Total Richness** | 27 | 43 | 39 | 36 | 41 | 48 | 60 | 52 | 43 |

Oneida South

|  | **1930** | **1940** | **1950** | **1960** | **1970** | **1980** | **1990** | **2000** | **2010** |
| --- | --- | --- | --- | --- | --- | --- | --- | --- | --- |
| Add (First Time) | 21 | 28 | 9 | 6 | 2 | 1 | 10 | 4 | 1 |
| Reappear (Had been absent 1 period) | NA | NA | 3 | 4 | 3 | 0 | 17 | 0 | 2 |
| Rejoin (Had been absent 2+ periods) | NA | NA | NA | 0 | 6 | 0 | 6 | 4 | 1 |
| Remained | NA | 13 | 20 | 27 | 33 | 21 | 22 | 44 | 32 |
| Missing (absent 1 period) | NA | 8 | 21 | 5 | 4 | 23 | 0 | 11 | 20 |
| Lost (absent 2 periods) | NA | NA | 5 | 17 | 2 | 4 | 6 | 0 | 9 |
|  |  |  |  |  |  |  |  |  |  |
| **Total Richness** | 21 | 41 | 32 | 37 | 44 | 22 | 55 | 52 | 36 |

Seneca River

|  | **1930** | **1940** | **1950** | **1960** | **1970** | **1980** | **1990** | **2000** |
| --- | --- | --- | --- | --- | --- | --- | --- | --- |
| Add (First Time) | 51 | 5 | 6 | 2 | 8 | 2 | 2 | 5 |
| Reappear (Had been absent 1 period) | NA | NA | 7 | 2 | 13 | 3 | 3 | 17 |
| Rejoin (Had been absent 2+ periods) | NA | NA | NA | 3 | 5 | 7 | 0 | 4 |
| Remained | NA | 33 | 29 | 24 | 26 | 44 | 32 | 30 |
| Missing (absent 1 period) | NA | 18 | 9 | 18 | 5 | 8 | 24 | 7 |
| Lost (absent 2 periods) | NA | NA | 11 | 7 | 5 | 2 | 5 | 7 |
|  |  |  |  |  |  |  |  |  |
| **Total Richness** | 51 | 38 | 42 | 31 | 52 | 56 | 37 | 56 |

Skaneateles

|  | **1940** | **1950** | **1960** | **1970** | **1980** | **1990** | **2000** |
| --- | --- | --- | --- | --- | --- | --- | --- |
| Add (First Time) | 29 | 1 | 6 | 5 | 2 | 1 | 3 |
| Reappear (Had been absent 1 period) | NA | NA | 10 | 0 | 1 | 4 | 3 |
| Rejoin (Had been absent 2+ periods) | NA | NA | NA | 1 | 2 | 1 | 0 |
| Remained | NA | 13 | 14 | 28 | 28 | 27 | 28 |
| Missing (absent 1 period) | NA | 16 | 0 | 2 | 6 | 6 | 5 |
| Lost (absent 2 periods) | NA | NA | 6 | 0 | 1 | 2 | 3 |
|  |  |  |  |  |  |  |  |
| **Total Richness** | 29 | 14 | 30 | 34 | 33 | 33 | 34 |

Syracuse

|  | **1930** | **1940** | **1950** | **1960** | **1970** | **1980** | **1990** | **2000** | **2010** |
| --- | --- | --- | --- | --- | --- | --- | --- | --- | --- |
| Add (First Time) | 39 | 5 | 16 | 9 | 6 | 2 | 7 | 0 | 5 |
| Reappear (Had been absent 1 period) | NA | NA | 11 | 5 | 4 | 0 | 6 | 2 | 2 |
| Rejoin (Had been absent 2+ periods) | NA | NA | NA | 0 | 2 | 2 | 4 | 2 | 0 |
| Remained | NA | 20 | 17 | 38 | 47 | 49 | 48 | 53 | 44 |
| Missing (absent 1 period) | NA | 19 | 8 | 6 | 5 | 10 | 5 | 12 | 13 |
| Lost (absent 2 periods) | NA | NA | 8 | 3 | 2 | 5 | 4 | 3 | 10 |
|  |  |  |  |  |  |  |  |  |  |
| **Total Richness** | 39 | 25 | 44 | 52 | 59 | 53 | 65 | 57 | 51 |
