# Supplementary material for: Land cover as a driver of fish community changes in New York’s Oswego River Watershed
Source: PLoS One. 2025 Jul 14;20(7):e0327293. doi: 10.1371/journal.pone.0327293 (PMC12258583; doi:10.1371/journal.pone.0327293)
Supplement: S6 Table — Table tracking presence and absence for each individual species in the full watershed and then in each sub-basin. A name indicates that the species was present, and a blank cell indicates that the species was absent. (DOCX) [file pone.0327293.s009.docx]

**S6 Table. Tracking the presence or absence of each species through the full watershed and each sub-basin over time.** Table tracking presence and absence for each individual species in the full watershed and then in each sub-basin**.** A name indicates that the species was present, and a blank cell indicates that the species was absent.

Full Watershed

| **1930** | **1940** | **1950** | **1960** | **1970** | **1980** | **1990** | **2000** | **2010** |
| --- | --- | --- | --- | --- | --- | --- | --- | --- |
| Alewife | Alewife | Alewife | Alewife | Alewife | Alewife | Alewife | Alewife | Alewife |
| Allegheny Pearl Dace | Allegheny Pearl Dace |  |  | Allegheny Pearl Dace |  |  |  |  |
| American Brook Lamprey |  | American Brook Lamprey | American Brook Lamprey |  | American Brook Lamprey |  |  |  |
| American Eel |  | American Eel | American Eel | American Eel | American Eel | American Eel |  |  |
|  |  | Atlantic Salmon | Atlantic Salmon | Atlantic Salmon | Atlantic Salmon | Atlantic Salmon | Atlantic Salmon | Atlantic Salmon |
| Banded Killifish | Banded Killifish | Banded Killifish | Banded Killifish | Banded Killifish | Banded Killifish | Banded Killifish | Banded Killifish | Banded Killifish |
| Bigmouth Shiner |  |  |  |  |  |  |  |  |
| Black Bullhead | Black Bullhead |  |  |  |  |  |  |  |
| Black Crappie | Black Crappie | Black Crappie | Black Crappie | Black Crappie | Black Crappie | Black Crappie | Black Crappie | Black Crappie |
| Blackchin Shiner | Blackchin Shiner | Blackchin Shiner | Blackchin Shiner |  |  |  |  |  |
| Blacknose Shiner | Blacknose Shiner | Blacknose Shiner |  |  |  | Blacknose Shiner | Blacknose Shiner |  |
| Blackside Darter | Blackside Darter | Blackside Darter |  | Blackside Darter | Blackside Darter | Blackside Darter | Blackside Darter | Blackside Darter |
| Blue Pike |  |  |  |  |  |  |  |  |
|  |  |  |  |  | Blueback Herring | Blueback Herring |  |  |
| Bluegill | Bluegill | Bluegill | Bluegill | Bluegill | Bluegill | Bluegill | Bluegill | Bluegill |
|  |  |  |  | Bluespotted Sunfish |  |  |  | Bluespotted Sunfish |
| Bluntnose Minnow | Bluntnose Minnow | Bluntnose Minnow | Bluntnose Minnow | Bluntnose Minnow | Bluntnose Minnow | Bluntnose Minnow | Bluntnose Minnow | Bluntnose Minnow |
|  |  | Bowfin | Bowfin | Bowfin | Bowfin | Bowfin | Bowfin | Bowfin |
|  |  |  | Brassy Minnow |  | Brassy Minnow | Brassy Minnow | Brassy Minnow |  |
| Bridle Shiner | Bridle Shiner |  | Bridle Shiner | Bridle Shiner | Bridle Shiner | Bridle Shiner | Bridle Shiner |  |
|  | Brindled Madtom | Brindled Madtom | Brindled Madtom |  |  |  | Brindled Madtom | Brindled Madtom |
| Brook Silverside | Brook Silverside | Brook Silverside | Brook Silverside | Brook Silverside | Brook Silverside | Brook Silverside | Brook Silverside | Brook Silverside |
| Brook Stickleback | Brook Stickleback | Brook Stickleback | Brook Stickleback | Brook Stickleback | Brook Stickleback | Brook Stickleback | Brook Stickleback | Brook Stickleback |
| Brook Trout | Brook Trout | Brook Trout | Brook Trout | Brook Trout | Brook Trout | Brook Trout | Brook Trout | Brook Trout |
| Brown Bullhead | Brown Bullhead | Brown Bullhead | Brown Bullhead | Brown Bullhead | Brown Bullhead | Brown Bullhead | Brown Bullhead | Brown Bullhead |
| Brown Trout | Brown Trout | Brown Trout | Brown Trout | Brown Trout | Brown Trout | Brown Trout | Brown Trout | Brown Trout |
| Burbot | Burbot | Burbot | Burbot | Burbot | Burbot | Burbot | Burbot |  |
| Central Mudminnow | Central Mudminnow | Central Mudminnow | Central Mudminnow | Central Mudminnow | Central Mudminnow | Central Mudminnow | Central Mudminnow | Central Mudminnow |
| Central Stoneroller | Central Stoneroller | Central Stoneroller | Central Stoneroller | Central Stoneroller | Central Stoneroller | Central Stoneroller | Central Stoneroller | Central Stoneroller |
| Chain Pickerel | Chain Pickerel | Chain Pickerel | Chain Pickerel | Chain Pickerel | Chain Pickerel | Chain Pickerel | Chain Pickerel | Chain Pickerel |
| Channel Catfish | Channel Catfish | Channel Catfish | Channel Catfish | Channel Catfish | Channel Catfish | Channel Catfish | Channel Catfish | Channel Catfish |
|  |  |  |  |  |  |  |  | Chinook Salmon |
| Cisco | Cisco | Cisco | Cisco | Cisco | Cisco | Cisco | Cisco |  |
|  |  |  |  |  |  |  |  | Coho Salmon |
|  |  |  |  |  |  |  | Comely Shiner | Comely Shiner |
| Common Carp | Common Carp | Common Carp | Common Carp | Common Carp | Common Carp | Common Carp | Common Carp | Common Carp |
| Common Shiner | Common Shiner | Common Shiner | Common Shiner | Common Shiner | Common Shiner | Common Shiner | Common Shiner | Common Shiner |
| Creek Chub | Creek Chub | Creek Chub | Creek Chub | Creek Chub | Creek Chub | Creek Chub | Creek Chub | Creek Chub |
| Cutlip Minnow | Cutlip Minnow | Cutlip Minnow | Cutlip Minnow | Cutlip Minnow | Cutlip Minnow | Cutlip Minnow | Cutlip Minnow | Cutlip Minnow |
| Eastern Blacknose Dace | Eastern Blacknose Dace | Eastern Blacknose Dace | Eastern Blacknose Dace | Eastern Blacknose Dace | Eastern Blacknose Dace | Eastern Blacknose Dace | Eastern Blacknose Dace | Eastern Blacknose Dace |
| Eastern Creek Chubsucker | Eastern Creek Chubsucker | Eastern Creek Chubsucker | Eastern Creek Chubsucker | Eastern Creek Chubsucker |  | Eastern Creek Chubsucker | Eastern Creek Chubsucker | Eastern Creek Chubsucker |
| Eastern Silvery Minnow | Eastern Silvery Minnow | Eastern Silvery Minnow | Eastern Silvery Minnow |  | Eastern Silvery Minnow | Eastern Silvery Minnow |  | Eastern Silvery Minnow |
| Emerald Shiner | Emerald Shiner | Emerald Shiner | Emerald Shiner | Emerald Shiner | Emerald Shiner | Emerald Shiner | Emerald Shiner | Emerald Shiner |
| Fallfish | Fallfish | Fallfish | Fallfish | Fallfish | Fallfish | Fallfish | Fallfish | Fallfish |
| Fantail Darter | Fantail Darter | Fantail Darter | Fantail Darter | Fantail Darter | Fantail Darter | Fantail Darter | Fantail Darter | Fantail Darter |
| Fathead Minnow | Fathead Minnow | Fathead Minnow | Fathead Minnow | Fathead Minnow | Fathead Minnow | Fathead Minnow | Fathead Minnow | Fathead Minnow |
| Freshwater Drum | Freshwater Drum | Freshwater Drum | Freshwater Drum | Freshwater Drum | Freshwater Drum | Freshwater Drum | Freshwater Drum | Freshwater Drum |
|  |  | Gizzard Shad | Gizzard Shad | Gizzard Shad | Gizzard Shad | Gizzard Shad | Gizzard Shad | Gizzard Shad |
|  |  | Golden Redhorse |  | Golden Redhorse |  |  |  |  |
| Golden Shiner | Golden Shiner | Golden Shiner | Golden Shiner | Golden Shiner | Golden Shiner | Golden Shiner | Golden Shiner | Golden Shiner |
| Goldfish |  | Goldfish | Goldfish | Goldfish | Goldfish | Goldfish | Goldfish |  |
| Grass Pickerel |  | Grass Pickerel | Grass Pickerel | Grass Pickerel |  | Grass Pickerel | Grass Pickerel | Grass Pickerel |
|  |  | Greater Redhorse | Greater Redhorse | Greater Redhorse |  | Greater Redhorse |  |  |
|  |  |  |  |  |  | Green Sunfish | Green Sunfish | Green Sunfish |
| Greenside Darter | Greenside Darter | Greenside Darter |  | Greenside Darter | Greenside Darter |  | Greenside Darter |  |
| Hornyhead Chub | Hornyhead Chub | Hornyhead Chub |  | Hornyhead Chub | Hornyhead Chub | Hornyhead Chub | Hornyhead Chub | Hornyhead Chub |
| Iowa Darter | Iowa Darter |  |  |  | Iowa Darter |  | Iowa Darter | Iowa Darter |
|  |  |  |  |  |  |  | Johnny Darter |  |
| Lake Chub |  |  |  |  |  |  |  |  |
|  |  | Lake Sturgeon | Lake Sturgeon | Lake Sturgeon | Lake Sturgeon | Lake Sturgeon | Lake Sturgeon | Lake Sturgeon |
| Lake Trout | Lake Trout | Lake Trout | Lake Trout | Lake Trout | Lake Trout | Lake Trout | Lake Trout | Lake Trout |
| Lake Whitefish | Lake Whitefish | Lake Whitefish | Lake Whitefish | Lake Whitefish | Lake Whitefish |  |  |  |
| Largemouth Bass | Largemouth Bass | Largemouth Bass | Largemouth Bass | Largemouth Bass | Largemouth Bass | Largemouth Bass | Largemouth Bass | Largemouth Bass |
| Logperch | Logperch | Logperch | Logperch | Logperch | Logperch | Logperch | Logperch | Logperch |
| Longnose Dace | Longnose Dace | Longnose Dace | Longnose Dace | Longnose Dace | Longnose Dace | Longnose Dace | Longnose Dace | Longnose Dace |
| Longnose Gar |  | Longnose Gar | Longnose Gar | Longnose Gar | Longnose Gar | Longnose Gar | Longnose Gar |  |
| Longnose Sucker | Longnose Sucker | Longnose Sucker | Longnose Sucker | Longnose Sucker | Longnose Sucker | Longnose Sucker | Longnose Sucker |  |
|  | Margined Madtom | Margined Madtom | Margined Madtom | Margined Madtom | Margined Madtom | Margined Madtom | Margined Madtom | Margined Madtom |
| Mimic Shiner | Mimic Shiner |  |  |  | Mimic Shiner | Mimic Shiner | Mimic Shiner | Mimic Shiner |
| Mottled Sculpin | Mottled Sculpin | Mottled Sculpin | Mottled Sculpin | Mottled Sculpin | Mottled Sculpin | Mottled Sculpin | Mottled Sculpin | Mottled Sculpin |
| Ninespine Stickleback | Ninespine Stickleback |  |  | Ninespine Stickleback |  |  |  |  |
| Northern Hogsucker | Northern Hogsucker | Northern Hogsucker | Northern Hogsucker | Northern Hogsucker | Northern Hogsucker | Northern Hogsucker | Northern Hogsucker | Northern Hogsucker |
| Northern Pearl Dace |  |  |  |  |  |  |  |  |
| Northern Pike | Northern Pike | Northern Pike | Northern Pike | Northern Pike | Northern Pike | Northern Pike | Northern Pike | Northern Pike |
| Northern Redbelly Dace | Northern Redbelly Dace | Northern Redbelly Dace | Northern Redbelly Dace | Northern Redbelly Dace |  | Northern Redbelly Dace | Northern Redbelly Dace |  |
|  | Northern Sunfish |  |  |  |  |  |  |  |
| Pumpkinseed | Pumpkinseed | Pumpkinseed | Pumpkinseed | Pumpkinseed | Pumpkinseed | Pumpkinseed | Pumpkinseed | Pumpkinseed |
|  |  | Quillback |  |  |  | Quillback |  |  |
|  | Rainbow Smelt | Rainbow Smelt | Rainbow Smelt | Rainbow Smelt | Rainbow Smelt | Rainbow Smelt | Rainbow Smelt | Rainbow Smelt |
| Rainbow Trout | Rainbow Trout | Rainbow Trout | Rainbow Trout | Rainbow Trout | Rainbow Trout | Rainbow Trout | Rainbow Trout | Rainbow Trout |
| Redside Dace | Redside Dace | Redside Dace | Redside Dace | Redside Dace | Redside Dace | Redside Dace | Redside Dace | Redside Dace |
| River Chub |  | River Chub | River Chub |  |  |  | River Chub |  |
| Rock Bass | Rock Bass | Rock Bass | Rock Bass | Rock Bass | Rock Bass | Rock Bass | Rock Bass | Rock Bass |
| Rosyface Shiner | Rosyface Shiner | Rosyface Shiner |  | Rosyface Shiner |  | Rosyface Shiner | Rosyface Shiner | Rosyface Shiner |
|  |  |  |  |  |  |  | Round Goby | Round Goby |
|  |  |  |  |  |  | Rudd | Rudd | Rudd |
|  |  |  |  |  |  | Sand Shiner |  |  |
|  | Satinfin Shiner | Satinfin Shiner | Satinfin Shiner | Satinfin Shiner |  | Satinfin Shiner | Satinfin Shiner | Satinfin Shiner |
|  | Sea Lamprey | Sea Lamprey | Sea Lamprey | Sea Lamprey | Sea Lamprey | Sea Lamprey | Sea Lamprey | Sea Lamprey |
| Shorthead Redhorse | Shorthead Redhorse | Shorthead Redhorse | Shorthead Redhorse | Shorthead Redhorse | Shorthead Redhorse | Shorthead Redhorse | Shorthead Redhorse | Shorthead Redhorse |
| Silver Redhorse | Silver Redhorse | Silver Redhorse | Silver Redhorse |  |  | Silver Redhorse | Silver Redhorse |  |
|  | Slimy Scuplin | Slimy Scuplin | Slimy Scuplin | Slimy Scuplin | Slimy Scuplin | Slimy Scuplin | Slimy Scuplin | Slimy Scuplin |
| Smallmouth Bass | Smallmouth Bass | Smallmouth Bass | Smallmouth Bass | Smallmouth Bass | Smallmouth Bass | Smallmouth Bass | Smallmouth Bass | Smallmouth Bass |
|  |  |  | Sockeye Salmon |  |  |  |  |  |
| Spotfin Shiner | Spotfin Shiner | Spotfin Shiner | Spotfin Shiner | Spotfin Shiner | Spotfin Shiner | Spotfin Shiner | Spotfin Shiner | Spotfin Shiner |
| Spottail Shiner | Spottail Shiner | Spottail Shiner | Spottail Shiner | Spottail Shiner | Spottail Shiner | Spottail Shiner | Spottail Shiner | Spottail Shiner |
|  | Stonecat | Stonecat | Stonecat | Stonecat | Stonecat | Stonecat | Stonecat | Stonecat |
| Striped Shiner | Striped Shiner |  |  | Striped Shiner | Striped Shiner |  | Striped Shiner | Striped Shiner |
| Swallowtail Shiner |  |  |  | Swallowtail Shiner |  |  |  |  |
| Tadpole Madtom | Tadpole Madtom |  | Tadpole Madtom | Tadpole Madtom | Tadpole Madtom | Tadpole Madtom | Tadpole Madtom | Tadpole Madtom |
| Tessellated Darter | Tessellated Darter | Tessellated Darter | Tessellated Darter | Tessellated Darter | Tessellated Darter | Tessellated Darter | Tessellated Darter | Tessellated Darter |
| Threespine Stickleback |  |  | Threespine Stickleback |  |  |  |  |  |
|  |  |  |  | Tiger Musky | Tiger Musky | Tiger Musky | Tiger Musky | Tiger Musky |
|  | Trout-Perch | Trout-Perch | Trout-Perch | Trout-Perch | Trout-Perch | Trout-Perch | Trout-Perch | Trout-Perch |
| Walleye | Walleye | Walleye | Walleye | Walleye | Walleye | Walleye | Walleye | Walleye |
| White Bass | White Bass |  |  | White Bass | White Bass | White Bass | White Bass |  |
|  |  |  | White Crappie | White Crappie | White Crappie | White Crappie | White Crappie | White Crappie |
|  | White Perch | White Perch | White Perch | White Perch | White Perch | White Perch | White Perch | White Perch |
| White Sucker | White Sucker | White Sucker | White Sucker | White Sucker | White Sucker | White Sucker | White Sucker | White Sucker |
| Yellow Bullhead | Yellow Bullhead |  | Yellow Bullhead | Yellow Bullhead | Yellow Bullhead | Yellow Bullhead | Yellow Bullhead | Yellow Bullhead |
| Yellow Perch | Yellow Perch | Yellow Perch | Yellow Perch | Yellow Perch | Yellow Perch | Yellow Perch | Yellow Perch | Yellow Perch |

Canandaigua

| **1930** | **1940** | **1950** | **1960** | **1970** | **1980** | **2000** |
| --- | --- | --- | --- | --- | --- | --- |
|  |  | Alewife | Alewife | Alewife |  | Alewife |
|  |  |  | Atlantic Salmon |  |  |  |
|  | Banded Killifish |  | Banded Killifish | Banded Killifish | Banded Killifish | Banded Killifish |
|  |  |  | Black Crappie | Black Crappie | Black Crappie |  |
|  |  | Blackside Darter |  |  |  |  |
|  |  |  | Bluegill | Bluegill | Bluegill | Bluegill |
| Bluntnose Minnow | Bluntnose Minnow | Bluntnose Minnow | Bluntnose Minnow | Bluntnose Minnow | Bluntnose Minnow | Bluntnose Minnow |
|  |  |  | Brassy Minnow |  |  |  |
| Brook Stickleback |  |  |  | Brook Stickleback | Brook Stickleback |  |
|  | Brook Trout | Brook Trout | Brook Trout | Brook Trout |  |  |
|  | Brown Bullhead |  | Brown Bullhead | Brown Bullhead | Brown Bullhead | Brown Bullhead |
|  |  | Brown Trout | Brown Trout | Brown Trout | Brown Trout | Brown Trout |
|  | Burbot | Burbot |  |  |  |  |
|  |  | Central Mudminnow |  | Central Mudminnow |  | Central Mudminnow |
| Central Stoneroller |  | Central Stoneroller | Central Stoneroller | Central Stoneroller | Central Stoneroller | Central Stoneroller |
| Chain Pickerel | Chain Pickerel | Chain Pickerel | Chain Pickerel | Chain Pickerel | Chain Pickerel | Chain Pickerel |
|  | Cisco | Cisco | Cisco |  |  |  |
|  | Common Carp |  | Common Carp | Common Carp | Common Carp | Common Carp |
| Common Shiner |  | Common Shiner | Common Shiner | Common Shiner | Common Shiner | Common Shiner |
| Creek Chub | Creek Chub | Creek Chub | Creek Chub | Creek Chub | Creek Chub | Creek Chub |
| Cutlip Minnow |  |  | Cutlip Minnow | Cutlip Minnow |  | Cutlip Minnow |
|  | Eastern Blacknose Dace | Eastern Blacknose Dace | Eastern Blacknose Dace | Eastern Blacknose Dace | Eastern Blacknose Dace | Eastern Blacknose Dace |
|  | Eastern Silvery Minnow | Eastern Silvery Minnow | Eastern Silvery Minnow |  |  |  |
| Fallfish |  |  |  |  | Fallfish |  |
|  | Fantail Darter | Fantail Darter | Fantail Darter | Fantail Darter | Fantail Darter | Fantail Darter |
|  |  |  |  | Fathead Minnow | Fathead Minnow | Fathead Minnow |
| Golden Shiner | Golden Shiner | Golden Shiner | Golden Shiner | Golden Shiner | Golden Shiner | Golden Shiner |
| Goldfish |  |  |  |  |  |  |
|  |  |  | Grass Pickerel | Grass Pickerel |  |  |
|  | Lake Trout | Lake Trout | Lake Trout | Lake Trout | Lake Trout | Lake Trout |
| Lake Whitefish | Lake Whitefish |  | Lake Whitefish |  |  |  |
|  | Largemouth Bass |  | Largemouth Bass | Largemouth Bass | Largemouth Bass | Largemouth Bass |
|  | Logperch | Logperch |  |  |  |  |
|  |  | Longnose Dace | Longnose Dace | Longnose Dace | Longnose Dace | Longnose Dace |
|  |  | Mottled Sculpin | Mottled Sculpin | Mottled Sculpin | Mottled Sculpin |  |
| Ninespine Stickleback |  |  |  | Ninespine Stickleback |  |  |
|  |  |  | Northern Hogsucker | Northern Hogsucker |  | Northern Hogsucker |
|  |  |  |  | Northern Pike |  |  |
|  | Pumpkinseed | Pumpkinseed | Pumpkinseed | Pumpkinseed | Pumpkinseed | Pumpkinseed |
|  |  | Quillback |  |  |  |  |
|  | Rainbow Smelt | Rainbow Smelt | Rainbow Smelt |  |  | Rainbow Smelt |
|  | Rainbow Trout | Rainbow Trout | Rainbow Trout | Rainbow Trout | Rainbow Trout | Rainbow Trout |
|  |  |  | Redside Dace |  |  |  |
| Rock Bass | Rock Bass | Rock Bass | Rock Bass | Rock Bass | Rock Bass | Rock Bass |
|  | Satinfin Shiner |  |  |  |  |  |
|  | Slimy Sculpin |  | Slimy Sculpin |  |  | Slimy Sculpin |
|  | Smallmouth Bass | Smallmouth Bass | Smallmouth Bass |  | Smallmouth Bass | Smallmouth Bass |
|  | Spotfin Shiner |  |  | Spotfin Shiner |  | Spotfin Shiner |
|  | Spottail Shiner |  |  | Spottail Shiner | Spottail Shiner | Spottail Shiner |
|  |  |  |  |  | Stonecat | Stonecat |
|  |  |  |  | Tadpole Madtom | Tadpole Madtom |  |
|  |  |  |  | Tessellated Darter | Tessellated Darter | Tessellated Darter |
|  | Walleye | Walleye |  |  |  |  |
| White Sucker | White Sucker | White Sucker | White Sucker | White Sucker | White Sucker | White Sucker |
| Yellow Perch | Yellow Perch | Yellow Perch | Yellow Perch | Yellow Perch | Yellow Perch | Yellow Perch |

Cayuga

| **1930** | **1940** | **1950** | **1960** | **1970** | **1980** | **1990** | **2000** | **2010** |
| --- | --- | --- | --- | --- | --- | --- | --- | --- |
|  | Alewife | Alewife | Alewife | Alewife | Alewife | Alewife | Alewife | Alewife |
| Allegheny Pearl Dace | Allegheny Pearl Dace |  |  |  |  |  |  |  |
| American Brook Lamprey |  | American Brook Lamprey | American Brook Lamprey |  | American Brook Lamprey |  |  |  |
|  |  | American Eel | American Eel |  |  | American Eel |  |  |
|  |  | Atlantic Salmon |  | Atlantic Salmon | Atlantic Salmon | Atlantic Salmon | Atlantic Salmon | Atlantic Salmon |
| Banded Killifish | Banded Killifish |  | Banded Killifish |  |  | Banded Killifish | Banded Killifish |  |
|  | Black Bullhead |  |  |  |  |  |  |  |
|  | Black Crappie | Black Crappie | Black Crappie | Black Crappie | Black Crappie | Black Crappie |  |  |
| Blackchin Shiner |  | Blackchin Shiner | Blackchin Shiner |  |  |  |  |  |
| Blacknose Shiner | Blacknose Shiner | Blacknose Shiner |  |  |  |  |  |  |
|  | Bluegill | Bluegill | Bluegill | Bluegill | Bluegill | Bluegill | Bluegill |  |
| Bluntnose Minnow | Bluntnose Minnow | Bluntnose Minnow | Bluntnose Minnow | Bluntnose Minnow | Bluntnose Minnow | Bluntnose Minnow | Bluntnose Minnow |  |
| Bridle Shiner |  |  |  |  |  | Bridle Shiner |  |  |
|  |  |  | Brindled Madtom |  |  |  |  |  |
|  |  |  | Brook Silverside |  |  | Brook Silverside | Brook Silverside |  |
| Brook Stickleback | Brook Stickleback |  |  | Brook Stickleback |  | Brook Stickleback | Brook Stickleback |  |
| Brook Trout |  | Brook Trout | Brook Trout | Brook Trout | Brook Trout | Brook Trout | Brook Trout | Brook Trout |
| Brown Bullhead | Brown Bullhead | Brown Bullhead | Brown Bullhead | Brown Bullhead | Brown Bullhead | Brown Bullhead | Brown Bullhead |  |
| Brown Trout |  | Brown Trout | Brown Trout | Brown Trout | Brown Trout | Brown Trout | Brown Trout |  |
|  |  | Central Stoneroller | Central Stoneroller | Central Stoneroller | Central Stoneroller | Central Stoneroller | Central Stoneroller |  |
| Chain Pickerel | Chain Pickerel |  | Chain Pickerel | Chain Pickerel | Chain Pickerel | Chain Pickerel | Chain Pickerel |  |
|  |  |  |  | Channel Catfish | Channel Catfish | Channel Catfish |  | Channel Catfish |
|  | Cisco |  |  | Cisco | Cisco |  |  |  |
|  |  |  | Common Carp | Common Carp | Common Carp | Common Carp | Common Carp | Common Carp |
| Common Shiner | Common Shiner | Common Shiner | Common Shiner | Common Shiner | Common Shiner | Common Shiner | Common Shiner | Common Shiner |
| Creek Chub | Creek Chub | Creek Chub | Creek Chub | Creek Chub | Creek Chub | Creek Chub | Creek Chub | Creek Chub |
| Cutlip Minnow | Cutlip Minnow | Cutlip Minnow | Cutlip Minnow | Cutlip Minnow | Cutlip Minnow | Cutlip Minnow | Cutlip Minnow | Cutlip Minnow |
| Eastern Blacknose Dace | Eastern Blacknose Dace | Eastern Blacknose Dace | Eastern Blacknose Dace | Eastern Blacknose Dace | Eastern Blacknose Dace | Eastern Blacknose Dace | Eastern Blacknose Dace | Eastern Blacknose Dace |
|  | Eastern Creek Chubsucker | Eastern Creek Chubsucker |  | Eastern Creek Chubsucker |  | Eastern Creek Chubsucker | Eastern Creek Chubsucker |  |
|  |  | Eastern Silvery Minnow |  |  |  |  |  |  |
|  |  | Emerald Shiner |  |  |  | Emerald Shiner |  |  |
|  |  | Fallfish |  | Fallfish | Fallfish | Fallfish | Fallfish |  |
| Fantail Darter |  | Fantail Darter | Fantail Darter | Fantail Darter | Fantail Darter | Fantail Darter | Fantail Darter | Fantail Darter |
|  |  | Fathead Minnow | Fathead Minnow | Fathead Minnow | Fathead Minnow | Fathead Minnow | Fathead Minnow |  |
|  |  |  |  |  |  |  |  | Freshwater Drum |
|  |  |  | Gizzard Shad | Gizzard Shad | Gizzard Shad | Gizzard Shad |  |  |
| Golden Shiner | Golden Shiner | Golden Shiner | Golden Shiner | Golden Shiner | Golden Shiner | Golden Shiner | Golden Shiner |  |
|  |  |  | Goldfish | Goldfish | Goldfish | Goldfish | Goldfish |  |
| Grass Pickerel |  |  |  |  |  |  |  |  |
|  |  | Lake Sturgeon | Lake Sturgeon |  |  | Lake Sturgeon | Lake Sturgeon | Lake Sturgeon |
|  | Lake Trout |  | Lake Trout | Lake Trout | Lake Trout | Lake Trout | Lake Trout | Lake Trout |
|  |  | Lake Whitefish |  |  |  |  |  |  |
| Largemouth Bass | Largemouth Bass | Largemouth Bass | Largemouth Bass | Largemouth Bass | Largemouth Bass | Largemouth Bass | Largemouth Bass |  |
| Longnose Dace | Longnose Dace | Longnose Dace | Longnose Dace | Longnose Dace | Longnose Dace | Longnose Dace | Longnose Dace |  |
|  |  | Longnose Gar | Longnose Gar | Longnose Gar | Longnose Gar | Longnose Gar | Longnose Gar |  |
|  |  | Margined Madtom |  |  |  | Margined Madtom | Margined Madtom |  |
|  | Mimic Shiner |  |  |  |  |  |  |  |
| Mottled Sculpin |  | Mottled Sculpin | Mottled Sculpin | Mottled Sculpin | Mottled Sculpin | Mottled Sculpin | Mottled Sculpin |  |
|  | Northern Hogsucker | Northern Hogsucker | Northern Hogsucker | Northern Hogsucker | Northern Hogsucker | Northern Hogsucker | Northern Hogsucker |  |
| Northern Pike |  |  | Northern Pike | Northern Pike | Northern Pike | Northern Pike |  |  |
|  | Northern Redbelly Dace | Northern Redbelly Dace | Northern Redbelly Dace |  |  |  |  |  |
| Pumpkinseed | Pumpkinseed | Pumpkinseed | Pumpkinseed | Pumpkinseed | Pumpkinseed | Pumpkinseed | Pumpkinseed | Pumpkinseed |
|  | Rainbow Smelt |  | Rainbow Smelt | Rainbow Smelt | Rainbow Smelt | Rainbow Smelt | Rainbow Smelt | Rainbow Smelt |
| Rainbow Trout |  | Rainbow Trout | Rainbow Trout | Rainbow Trout | Rainbow Trout | Rainbow Trout | Rainbow Trout | Rainbow Trout |
|  |  |  | Redside Dace |  |  |  | Redside Dace |  |
|  |  | River Chub | River Chub |  |  |  |  |  |
| Rock Bass | Rock Bass | Rock Bass | Rock Bass | Rock Bass | Rock Bass | Rock Bass | Rock Bass | Rock Bass |
|  |  | Rosyface Shiner |  |  |  |  |  |  |
|  |  |  |  |  |  |  |  | Round Goby |
|  |  |  |  |  |  | Rudd |  |  |
|  |  | Satinfin Shiner | Satinfin Shiner |  |  |  |  |  |
|  |  | Sea Lamprey | Sea Lamprey | Sea Lamprey | Sea Lamprey | Sea Lamprey | Sea Lamprey | Sea Lamprey |
|  |  |  |  | Slimy Sculpin | Slimy Sculpin | Slimy Sculpin | Slimy Sculpin | Slimy Sculpin |
|  |  | Smallmouth Bass | Smallmouth Bass | Smallmouth Bass | Smallmouth Bass | Smallmouth Bass | Smallmouth Bass |  |
| Spotfin Shiner |  | Spotfin Shiner | Spotfin Shiner | Spotfin Shiner |  | Spotfin Shiner | Spotfin Shiner |  |
| Spottail Shiner |  | Spottail Shiner | Spottail Shiner | Spottail Shiner |  | Spottail Shiner | Spottail Shiner |  |
|  |  |  |  | Stonecat |  |  |  |  |
| Striped Shiner | Striped Shiner |  |  |  |  |  | Striped Shiner |  |
|  |  |  | Tadpole Madtom |  |  |  | Tadpole Madtom |  |
| Tessellated Darter | Tessellated Darter | Tessellated Darter | Tessellated Darter | Tessellated Darter |  | Tessellated Darter | Tessellated Darter |  |
|  |  |  | Threespine Stickleback |  |  |  |  |  |
|  |  |  |  |  |  | Tiger Musky |  |  |
|  |  | Trout-Perch | Trout-Perch | Trout-Perch | Trout-Perch | Trout-Perch |  | Trout-Perch |
| Walleye | Walleye |  | Walleye | Walleye | Walleye | Walleye | Walleye |  |
| White Bass |  |  |  |  |  |  |  |  |
|  |  |  |  | White Perch | White Perch | White Perch |  |  |
| White Sucker | White Sucker | White Sucker | White Sucker | White Sucker | White Sucker | White Sucker | White Sucker | White Sucker |
|  |  |  | Yellow Bullhead |  |  |  |  |  |
| Yellow Perch | Yellow Perch | Yellow Perch | Yellow Perch | Yellow Perch | Yellow Perch | Yellow Perch | Yellow Perch | Yellow Perch |

Keuka

| **1930** | **1940** | **1950** | **1960** | **1970** | **1980** | **1990** | **2000** | **2010** |
| --- | --- | --- | --- | --- | --- | --- | --- | --- |
| Alewife | Alewife | Alewife | Alewife | Alewife | Alewife | Alewife | Alewife | Alewife |
|  | Allegheny Pearl Dace |  |  |  |  |  |  |  |
|  |  |  | Atlantic Salmon |  |  |  | Atlantic Salmon | Atlantic Salmon |
| Banded Killifish |  |  | Banded Killifish | Banded Killifish | Banded Killifish |  | Banded Killifish | Banded Killifish |
|  |  |  |  | Black Crappie | Black Crappie |  |  |  |
| Bluegill |  | Bluegill | Bluegill | Bluegill | Bluegill | Bluegill | Bluegill | Bluegill |
| Bluntnose Minnow |  | Bluntnose Minnow | Bluntnose Minnow | Bluntnose Minnow | Bluntnose Minnow | Bluntnose Minnow | Bluntnose Minnow | Bluntnose Minnow |
|  |  |  | Bridle Shiner | Bridle Shiner |  |  | Bridle Shiner |  |
|  | Brindled Madtom |  |  |  |  |  |  |  |
|  | Brook Silverside |  |  | Brook Silverside | Brook Silverside | Brook Silverside | Brook Silverside |  |
| Brook Stickleback |  |  |  | Brook Stickleback |  |  | Brook Stickleback |  |
|  |  | Brook Trout | Brook Trout | Brook Trout |  |  |  |  |
| Brown Bullhead | Brown Bullhead | Brown Bullhead | Brown Bullhead | Brown Bullhead | Brown Bullhead | Brown Bullhead | Brown Bullhead | Brown Bullhead |
|  | Brown Trout | Brown Trout | Brown Trout | Brown Trout | Brown Trout | Brown Trout | Brown Trout | Brown Trout |
|  |  |  |  |  |  | Central Mudminnow | Central Mudminnow |  |
| Central Stoneroller | Central Stoneroller | Central Stoneroller | Central Stoneroller | Central Stoneroller | Central Stoneroller | Central Stoneroller | Central Stoneroller |  |
| Chain Pickerel | Chain Pickerel | Chain Pickerel | Chain Pickerel | Chain Pickerel | Chain Pickerel | Chain Pickerel | Chain Pickerel | Chain Pickerel |
|  |  | Channel Catfish | Channel Catfish |  | Channel Catfish |  |  |  |
| Cisco | Cisco | Cisco | Cisco | Cisco | Cisco |  |  |  |
|  |  |  |  |  |  |  | Comely Shiner | Comely Shiner |
| Common Carp |  | Common Carp | Common Carp | Common Carp | Common Carp | Common Carp | Common Carp | Common Carp |
|  | Common Shiner | Common Shiner | Common Shiner | Common Shiner | Common Shiner | Common Shiner | Common Shiner |  |
| Creek Chub | Creek Chub | Creek Chub | Creek Chub | Creek Chub | Creek Chub | Creek Chub | Creek Chub | Creek Chub |
| Cutlip Minnow |  |  |  | Cutlip Minnow |  |  | Cutlip Minnow |  |
|  | Eastern Blacknose Dace | Eastern Blacknose Dace | Eastern Blacknose Dace | Eastern Blacknose Dace | Eastern Blacknose Dace |  | Eastern Blacknose Dace | Eastern Blacknose Dace |
|  | Eastern Silvery Minnow |  | Eastern Silvery Minnow |  | Eastern Silvery Minnow |  |  |  |
|  |  | Fantail Darter |  | Fantail Darter | Fantail Darter |  | Fantail Darter |  |
|  |  |  |  | Fathead Minnow |  |  | Fathead Minnow |  |
|  |  |  |  |  |  | Freshwater Drum |  |  |
|  |  |  |  |  | Gizzard Shad | Gizzard Shad |  |  |
| Golden Shiner | Golden Shiner |  | Golden Shiner | Golden Shiner | Golden Shiner | Golden Shiner | Golden Shiner | Golden Shiner |
|  |  | Goldfish |  | Goldfish |  |  |  |  |
|  |  |  | Lake Sturgeon |  |  |  |  |  |
|  | Lake Trout | Lake Trout | Lake Trout | Lake Trout | Lake Trout |  | Lake Trout | Lake Trout |
| Lake Whitefish |  |  |  |  |  |  |  |  |
| Largemouth Bass | Largemouth Bass | Largemouth Bass | Largemouth Bass | Largemouth Bass | Largemouth Bass | Largemouth Bass | Largemouth Bass | Largemouth Bass |
|  |  |  |  | Logperch |  | Logperch |  |  |
| Longnose Dace |  | Longnose Dace | Longnose Dace | Longnose Dace | Longnose Dace |  | Longnose Dace |  |
|  |  | Longnose Sucker |  |  |  |  |  |  |
|  |  |  |  | Margined Madtom | Margined Madtom | Margined Madtom | Margined Madtom |  |
|  |  | Mottled Sculpin |  | Mottled Sculpin |  |  |  |  |
|  | Ninespine Stickleback |  |  |  |  |  |  |  |
| Northern Hogsucker | Northern Hogsucker | Northern Hogsucker | Northern Hogsucker | Northern Hogsucker | Northern Hogsucker | Northern Hogsucker | Northern Hogsucker |  |
|  |  | Northern Pike | Northern Pike | Northern Pike | Northern Pike | Northern Pike | Northern Pike | Northern Pike |
| Pumpkinseed | Pumpkinseed | Pumpkinseed | Pumpkinseed | Pumpkinseed | Pumpkinseed | Pumpkinseed | Pumpkinseed | Pumpkinseed |
|  |  |  | Rainbow Smelt | Rainbow Smelt | Rainbow Smelt |  | Rainbow Smelt | Rainbow Smelt |
| Rainbow Trout | Rainbow Trout | Rainbow Trout | Rainbow Trout | Rainbow Trout | Rainbow Trout | Rainbow Trout | Rainbow Trout | Rainbow Trout |
| Redside Dace |  | Redside Dace |  | Redside Dace |  |  |  | Redside Dace |
| River Chub |  |  |  |  |  |  | River Chub |  |
|  | Rock Bass | Rock Bass | Rock Bass | Rock Bass | Rock Bass | Rock Bass | Rock Bass | Rock Bass |
|  |  |  |  |  |  |  |  | Rosyface Shiner |
|  |  |  |  |  |  |  |  | Rudd |
|  |  |  |  | Satinfin Shiner |  | Satinfin Shiner | Satinfin Shiner |  |
|  |  | Sea Lamprey | Sea Lamprey | Sea Lamprey | Sea Lamprey |  | Sea Lamprey | Sea Lamprey |
|  |  |  |  |  | Shorthead Redhorse |  |  |  |
|  |  |  |  | Slimy Sculpin |  |  | Slimy Sculpin | Slimy Sculpin |
|  | Smallmouth Bass | Smallmouth Bass | Smallmouth Bass | Smallmouth Bass | Smallmouth Bass | Smallmouth Bass | Smallmouth Bass | Smallmouth Bass |
|  |  |  | Spotfin Shiner | Spotfin Shiner | Spotfin Shiner | Spotfin Shiner | Spotfin Shiner | Spotfin Shiner |
| Spottail Shiner |  |  | Spottail Shiner | Spottail Shiner |  |  | Spottail Shiner | Spottail Shiner |
|  | Stonecat |  |  | Stonecat | Stonecat |  |  |  |
|  | Striped Shiner |  |  |  |  |  |  |  |
| Swallowtail Shiner |  |  |  | Swallowtail Shiner |  |  |  |  |
|  |  |  |  | Tadpole Madtom |  |  | Tadpole Madtom |  |
|  |  | Tessellated Darter | Tessellated Darter | Tessellated Darter | Tessellated Darter |  | Tessellated Darter | Tessellated Darter |
|  |  |  |  | Trout-Perch |  |  | Trout-Perch |  |
|  | White Bass |  |  |  |  |  |  |  |
|  |  |  | White Perch | White Perch |  |  |  |  |
| White Sucker | White Sucker | White Sucker | White Sucker | White Sucker | White Sucker | White Sucker | White Sucker | White Sucker |
|  |  |  |  |  |  |  |  | Yellow Bullhead |
| Yellow Perch | Yellow Perch | Yellow Perch | Yellow Perch | Yellow Perch | Yellow Perch | Yellow Perch | Yellow Perch | Yellow Perch |

Oneida North

| **1930** | **1940** | **1950** | **1960** | **1970** | **1980** | **1990** | **2000** | **2010** |
| --- | --- | --- | --- | --- | --- | --- | --- | --- |
|  |  |  | American Eel |  |  |  |  |  |
|  |  |  |  |  |  | Atlantic Salmon | Atlantic Salmon | Atlantic Salmon |
|  | Banded Killifish |  |  |  | Banded Killifish |  |  | Banded Killifish |
| Bigmouth Shiner |  |  |  |  |  |  |  |  |
|  |  |  |  |  | Black Crappie | Black Crappie | Black Crappie | Black Crappie |
| Blacknose Shiner |  |  |  |  |  | Blacknose Shiner | Blacknose Shiner |  |
|  | Blackside Darter | Blackside Darter |  | Blackside Darter | Blackside Darter | Blackside Darter | Blackside Darter | Blackside Darter |
|  |  |  |  |  | Blueback Herring | Blueback Herring |  |  |
| Bluegill | Bluegill | Bluegill | Bluegill | Bluegill | Bluegill | Bluegill | Bluegill | Bluegill |
|  | Bluntnose Minnow | Bluntnose Minnow | Bluntnose Minnow | Bluntnose Minnow | Bluntnose Minnow | Bluntnose Minnow | Bluntnose Minnow | Bluntnose Minnow |
|  |  |  |  |  |  |  | Bowfin | Bowfin |
|  |  |  |  |  |  | Brassy Minnow | Brassy Minnow |  |
|  | Bridle Shiner |  |  |  |  |  |  |  |
| Brook Stickleback |  | Brook Stickleback | Brook Stickleback | Brook Stickleback | Brook Stickleback | Brook Stickleback | Brook Stickleback | Brook Stickleback |
| Brook Trout | Brook Trout | Brook Trout | Brook Trout | Brook Trout | Brook Trout | Brook Trout | Brook Trout | Brook Trout |
|  | Brown Bullhead | Brown Bullhead | Brown Bullhead | Brown Bullhead | Brown Bullhead | Brown Bullhead | Brown Bullhead | Brown Bullhead |
| Brown Trout | Brown Trout | Brown Trout | Brown Trout | Brown Trout | Brown Trout | Brown Trout | Brown Trout | Brown Trout |
|  |  | Burbot | Burbot | Burbot | Burbot | Burbot |  |  |
| Central Mudminnow |  | Central Mudminnow |  | Central Mudminnow | Central Mudminnow | Central Mudminnow | Central Mudminnow | Central Mudminnow |
|  | Central Stoneroller |  |  | Central Stoneroller |  | Central Stoneroller |  |  |
| Chain Pickerel | Chain Pickerel | Chain Pickerel | Chain Pickerel | Chain Pickerel | Chain Pickerel | Chain Pickerel | Chain Pickerel | Chain Pickerel |
|  |  |  |  |  |  |  | Channel Catfish |  |
|  | Common Carp | Common Carp |  |  | Common Carp | Common Carp | Common Carp | Common Carp |
|  | Common Shiner | Common Shiner | Common Shiner | Common Shiner | Common Shiner | Common Shiner | Common Shiner | Common Shiner |
| Creek Chub | Creek Chub | Creek Chub | Creek Chub | Creek Chub | Creek Chub | Creek Chub | Creek Chub | Creek Chub |
| Cutlip Minnow | Cutlip Minnow | Cutlip Minnow | Cutlip Minnow | Cutlip Minnow | Cutlip Minnow | Cutlip Minnow | Cutlip Minnow | Cutlip Minnow |
|  | Eastern Blacknose Dace | Eastern Blacknose Dace | Eastern Blacknose Dace | Eastern Blacknose Dace | Eastern Blacknose Dace | Eastern Blacknose Dace | Eastern Blacknose Dace | Eastern Blacknose Dace |
|  |  | Eastern Creek Chubsucker | Eastern Creek Chubsucker | Eastern Creek Chubsucker |  | Eastern Creek Chubsucker | Eastern Creek Chubsucker | Eastern Creek Chubsucker |
|  | Eastern Silvery Minnow |  |  |  |  | Eastern Silvery Minnow |  | Eastern Silvery Minnow |
|  | Emerald Shiner | Emerald Shiner |  | Emerald Shiner |  | Emerald Shiner |  | Emerald Shiner |
| Fallfish | Fallfish | Fallfish | Fallfish | Fallfish | Fallfish | Fallfish | Fallfish | Fallfish |
| Fantail Darter | Fantail Darter | Fantail Darter | Fantail Darter | Fantail Darter | Fantail Darter | Fantail Darter | Fantail Darter | Fantail Darter |
|  | Fathead Minnow | Fathead Minnow | Fathead Minnow | Fathead Minnow | Fathead Minnow | Fathead Minnow | Fathead Minnow |  |
|  |  |  |  |  | Freshwater Drum | Freshwater Drum | Freshwater Drum | Freshwater Drum |
|  |  |  |  |  | Gizzard Shad | Gizzard Shad |  | Gizzard Shad |
| Golden Shiner | Golden Shiner | Golden Shiner | Golden Shiner | Golden Shiner | Golden Shiner | Golden Shiner | Golden Shiner | Golden Shiner |
| Grass Pickerel |  |  | Grass Pickerel |  |  |  | Grass Pickerel |  |
|  |  | Greater Redhorse | Greater Redhorse |  |  |  |  |  |
|  |  |  |  |  |  | Green Sunfish | Green Sunfish | Green Sunfish |
|  |  |  |  |  |  | Hornyhead Chub | Hornyhead Chub |  |
| Iowa Darter | Iowa Darter |  |  |  |  |  |  | Iowa Darter |
|  |  |  |  |  |  |  | Lake Sturgeon |  |
| Largemouth Bass | Largemouth Bass | Largemouth Bass | Largemouth Bass | Largemouth Bass |  | Largemouth Bass | Largemouth Bass | Largemouth Bass |
|  | Logperch | Logperch | Logperch | Logperch | Logperch | Logperch | Logperch | Logperch |
|  | Longnose Dace | Longnose Dace | Longnose Dace | Longnose Dace | Longnose Dace | Longnose Dace | Longnose Dace | Longnose Dace |
|  |  |  |  |  | Longnose Sucker |  |  |  |
|  |  | Margined Madtom | Margined Madtom | Margined Madtom | Margined Madtom | Margined Madtom | Margined Madtom |  |
|  |  |  |  |  |  | Mimic Shiner |  |  |
| Northern Hogsucker |  | Northern Hogsucker | Northern Hogsucker | Northern Hogsucker | Northern Hogsucker | Northern Hogsucker | Northern Hogsucker | Northern Hogsucker |
| Northern Pearl Dace |  |  |  |  |  |  |  |  |
|  |  |  |  | Northern Pike | Northern Pike | Northern Pike | Northern Pike |  |
|  |  |  |  | Northern Redbelly Dace |  | Northern Redbelly Dace | Northern Redbelly Dace |  |
|  | Northern Sunfish |  |  |  |  |  |  |  |
| Pumpkinseed | Pumpkinseed | Pumpkinseed | Pumpkinseed | Pumpkinseed | Pumpkinseed | Pumpkinseed | Pumpkinseed | Pumpkinseed |
| Rainbow Trout |  | Rainbow Trout | Rainbow Trout | Rainbow Trout | Rainbow Trout | Rainbow Trout | Rainbow Trout |  |
| Redside Dace | Redside Dace | Redside Dace | Redside Dace | Redside Dace | Redside Dace | Redside Dace | Redside Dace |  |
|  | Rock Bass | Rock Bass | Rock Bass | Rock Bass | Rock Bass | Rock Bass | Rock Bass | Rock Bass |
|  | Rosyface Shiner |  |  |  |  | Rosyface Shiner | Rosyface Shiner | Rosyface Shiner |
|  |  |  |  |  |  | Sand Shiner |  |  |
|  | Satinfin Shiner |  |  |  |  | Satinfin Shiner | Satinfin Shiner | Satinfin Shiner |
|  | Sea Lamprey |  |  | Sea Lamprey | Sea Lamprey | Sea Lamprey |  |  |
|  |  |  |  |  | Shorthead Redhorse | Shorthead Redhorse |  | Shorthead Redhorse |
|  |  | Silver Redhorse |  |  |  |  |  |  |
|  |  | Slimy Sculpin | Slimy Sculpin | Slimy Sculpin | Slimy Sculpin | Slimy Sculpin | Slimy Sculpin | Slimy Sculpin |
| Smallmouth Bass | Smallmouth Bass | Smallmouth Bass | Smallmouth Bass | Smallmouth Bass | Smallmouth Bass | Smallmouth Bass | Smallmouth Bass | Smallmouth Bass |
|  | Spottail Shiner |  | Spottail Shiner | Spottail Shiner | Spottail Shiner | Spottail Shiner |  |  |
|  | Stonecat | Stonecat | Stonecat | Stonecat | Stonecat | Stonecat | Stonecat |  |
| Tadpole Madtom | Tadpole Madtom |  |  |  | Tadpole Madtom |  |  |  |
|  | Tessellated Darter | Tessellated Darter |  | Tessellated Darter | Tessellated Darter | Tessellated Darter | Tessellated Darter | Tessellated Darter |
|  |  |  |  |  |  | Tiger Musky | Tiger Musky |  |
|  | Trout-Perch |  |  |  |  |  |  |  |
| Walleye | Walleye | Walleye | Walleye | Walleye | Walleye | Walleye | Walleye | Walleye |
|  |  |  |  |  | White Bass |  |  |  |
|  |  |  |  |  |  | White Crappie |  |  |
|  | White Perch |  |  |  | White Perch |  |  |  |
| White Sucker | White Sucker | White Sucker | White Sucker | White Sucker | White Sucker | White Sucker | White Sucker | White Sucker |
| Yellow Bullhead | Yellow Bullhead |  |  |  | Yellow Bullhead | Yellow Bullhead | Yellow Bullhead |  |
| Yellow Perch | Yellow Perch | Yellow Perch | Yellow Perch | Yellow Perch | Yellow Perch | Yellow Perch | Yellow Perch | Yellow Perch |

Oneida South

| **1930** | **1940** | **1950** | **1960** | **1970** | **1980** | **1990** | **2000** | **2010** |
| --- | --- | --- | --- | --- | --- | --- | --- | --- |
|  |  |  |  |  |  |  | Alewife |  |
| Allegheny Pearl Dace |  |  |  | Allegheny Pearl Dace |  |  |  |  |
| Banded Killifish | Banded Killifish | Banded Killifish | Banded Killifish | Banded Killifish | Banded Killifish | Banded Killifish | Banded Killifish | Banded Killifish |
|  | Black Crappie |  |  | Black Crappie |  | Black Crappie | Black Crappie | Black Crappie |
| Blacknose Shiner |  |  |  |  |  |  |  |  |
|  | Blackside Darter |  |  |  |  | Blackside Darter | Blackside Darter |  |
|  |  |  |  |  |  | Blueback Herring |  |  |
|  |  | Bluegill | Bluegill | Bluegill | Bluegill | Bluegill | Bluegill | Bluegill |
|  |  |  |  | Bluespotted Sunfish |  |  |  | Bluespotted Sunfish |
|  | Bluntnose Minnow |  | Bluntnose Minnow | Bluntnose Minnow | Bluntnose Minnow | Bluntnose Minnow | Bluntnose Minnow | Bluntnose Minnow |
|  |  |  |  |  |  |  | Bowfin |  |
|  | Bridle Shiner |  |  |  |  |  |  |  |
|  |  | Brindled Madtom |  |  |  |  |  |  |
|  |  |  |  |  |  |  | Brook Silverside | Brook Silverside |
|  |  | Brook Stickleback |  | Brook Stickleback |  |  | Brook Stickleback | Brook Stickleback |
| Brook Trout |  | Brook Trout | Brook Trout | Brook Trout |  | Brook Trout | Brook Trout | Brook Trout |
|  | Brown Bullhead | Brown Bullhead | Brown Bullhead | Brown Bullhead |  | Brown Bullhead | Brown Bullhead | Brown Bullhead |
| Brown Trout | Brown Trout | Brown Trout | Brown Trout | Brown Trout | Brown Trout | Brown Trout | Brown Trout |  |
|  | Burbot |  | Burbot | Burbot |  | Burbot | Burbot |  |
|  |  | Central Mudminnow | Central Mudminnow | Central Mudminnow |  | Central Mudminnow | Central Mudminnow |  |
|  |  | Central Stoneroller | Central Stoneroller | Central Stoneroller |  | Central Stoneroller | Central Stoneroller |  |
| Chain Pickerel | Chain Pickerel | Chain Pickerel | Chain Pickerel | Chain Pickerel |  | Chain Pickerel | Chain Pickerel | Chain Pickerel |
|  | Channel Catfish |  |  |  |  |  |  |  |
|  | Cisco |  |  |  |  |  |  |  |
| Common Carp | Common Carp |  | Common Carp | Common Carp | Common Carp | Common Carp | Common Carp | Common Carp |
|  | Common Shiner | Common Shiner | Common Shiner | Common Shiner | Common Shiner | Common Shiner | Common Shiner | Common Shiner |
|  | Creek Chub | Creek Chub | Creek Chub | Creek Chub | Creek Chub | Creek Chub | Creek Chub | Creek Chub |
| Cutlip Minnow |  | Cutlip Minnow | Cutlip Minnow | Cutlip Minnow | Cutlip Minnow | Cutlip Minnow | Cutlip Minnow | Cutlip Minnow |
|  | Eastern Blacknose Dace | Eastern Blacknose Dace | Eastern Blacknose Dace | Eastern Blacknose Dace | Eastern Blacknose Dace | Eastern Blacknose Dace | Eastern Blacknose Dace | Eastern Blacknose Dace |
|  | Eastern Creek Chubsucker |  |  |  |  | Eastern Creek Chubsucker |  |  |
|  | Eastern Silvery Minnow |  |  |  |  | Eastern Silvery Minnow |  |  |
|  | Emerald Shiner |  |  | Emerald Shiner |  | Emerald Shiner | Emerald Shiner | Emerald Shiner |
|  | Fallfish | Fallfish | Fallfish | Fallfish | Fallfish | Fallfish | Fallfish |  |
|  | Fantail Darter | Fantail Darter | Fantail Darter | Fantail Darter | Fantail Darter | Fantail Darter | Fantail Darter |  |
|  |  |  | Fathead Minnow | Fathead Minnow |  | Fathead Minnow | Fathead Minnow |  |
|  |  |  |  |  |  | Freshwater Drum | Freshwater Drum |  |
|  |  |  |  |  |  | Gizzard Shad |  | Gizzard Shad |
|  | Golden Shiner |  | Golden Shiner | Golden Shiner |  | Golden Shiner | Golden Shiner | Golden Shiner |
|  |  |  |  |  |  | Goldfish |  |  |
|  |  |  |  |  |  | Greater Redhorse |  |  |
|  |  |  |  |  |  | Green Sunfish | Green Sunfish | Green Sunfish |
|  | Hornyhead Chub |  |  |  |  |  |  |  |
|  | Iowa Darter |  |  |  |  |  |  |  |
|  |  |  |  | Lake Sturgeon |  |  | Lake Sturgeon |  |
| Lake Trout |  |  |  |  |  |  |  |  |
| Largemouth Bass | Largemouth Bass | Largemouth Bass | Largemouth Bass | Largemouth Bass |  | Largemouth Bass | Largemouth Bass | Largemouth Bass |
|  | Logperch | Logperch |  | Logperch | Logperch | Logperch | Logperch | Logperch |
|  |  | Longnose Dace | Longnose Dace | Longnose Dace | Longnose Dace | Longnose Dace | Longnose Dace |  |
|  |  |  | Longnose Sucker |  |  |  |  |  |
|  | Margined Madtom |  |  | Margined Madtom |  | Margined Madtom |  |  |
|  |  |  |  |  | Mimic Shiner | Mimic Shiner |  | Mimic Shiner |
|  | Mottled Sculpin |  |  |  |  |  | Mottled Sculpin |  |
| Northern Hogsucker |  | Northern Hogsucker | Northern Hogsucker | Northern Hogsucker | Northern Hogsucker | Northern Hogsucker | Northern Hogsucker |  |
| Northern Pearl Dace |  |  |  |  |  |  |  |  |
| Northern Pike | Northern Pike | Northern Pike | Northern Pike |  |  | Northern Pike | Northern Pike | Northern Pike |
| Northern Redbelly Dace |  |  |  |  |  |  |  |  |
| Pumpkinseed | Pumpkinseed | Pumpkinseed | Pumpkinseed | Pumpkinseed | Pumpkinseed | Pumpkinseed | Pumpkinseed | Pumpkinseed |
| Rainbow Trout | Rainbow Trout | Rainbow Trout | Rainbow Trout | Rainbow Trout |  | Rainbow Trout |  |  |
|  |  | Redside Dace |  | Redside Dace |  | Redside Dace |  |  |
| Rock Bass | Rock Bass | Rock Bass | Rock Bass | Rock Bass | Rock Bass | Rock Bass | Rock Bass | Rock Bass |
|  |  |  |  |  |  |  | Rosyface Shiner | Rosyface Shiner |
|  | Satinfin Shiner |  |  |  |  |  |  |  |
|  |  | Sea Lamprey |  |  |  |  |  |  |
|  | Shorthead Redhorse |  |  |  |  | Shorthead Redhorse |  |  |
|  |  | Silver Redhorse | Silver Redhorse |  |  |  |  |  |
|  |  |  | Slimy Sculpin | Slimy Sculpin |  | Slimy Sculpin | Slimy Sculpin |  |
| Smallmouth Bass | Smallmouth Bass | Smallmouth Bass | Smallmouth Bass | Smallmouth Bass | Smallmouth Bass | Smallmouth Bass | Smallmouth Bass | Smallmouth Bass |
|  |  |  | Sockeye Salmon |  |  |  |  |  |
|  |  |  |  |  |  | Spotfin Shiner | Spotfin Shiner | Spotfin Shiner |
|  | Spottail Shiner |  |  |  |  | Spottail Shiner | Spottail Shiner | Spottail Shiner |
|  |  |  | Stonecat | Stonecat |  | Stonecat | Stonecat |  |
|  |  |  |  |  |  | Tadpole Madtom | Tadpole Madtom |  |
|  | Tessellated Darter | Tessellated Darter | Tessellated Darter | Tessellated Darter | Tessellated Darter | Tessellated Darter | Tessellated Darter | Tessellated Darter |
|  |  |  |  |  |  | Tiger Musky | Tiger Musky | Tiger Musky |
|  |  |  |  |  |  | Trout-Perch | Trout-Perch |  |
| Walleye | Walleye | Walleye | Walleye | Walleye | Walleye | Walleye | Walleye | Walleye |
|  | White Bass |  |  | White Bass |  |  | White Bass |  |
|  |  |  | White Crappie | White Crappie |  |  |  |  |
|  | White Perch |  |  | White Perch |  | White Perch | White Perch | White Perch |
| White Sucker | White Sucker | White Sucker | White Sucker | White Sucker | White Sucker | White Sucker | White Sucker | White Sucker |
|  |  |  |  |  |  |  |  | Yellow Bullhead |
| Yellow Perch | Yellow Perch | Yellow Perch | Yellow Perch | Yellow Perch | Yellow Perch | Yellow Perch | Yellow Perch | Yellow Perch |

Seneca River

| **1930** | **1940** | **1950** | **1960** | **1970** | **1980** | **1990** | **2000** |
| --- | --- | --- | --- | --- | --- | --- | --- |
|  |  |  |  | Alewife | Alewife |  |  |
|  |  |  |  |  |  | Atlantic Salmon |  |
| Banded Killifish | Banded Killifish | Banded Killifish | Banded Killifish | Banded Killifish | Banded Killifish |  | Banded Killifish |
| Black Crappie | Black Crappie | Black Crappie | Black Crappie | Black Crappie | Black Crappie | Black Crappie | Black Crappie |
|  | Blackchin Shiner |  |  |  |  |  |  |
| Blacknose Shiner |  |  |  |  |  |  |  |
| Blackside Darter | Blackside Darter | Blackside Darter |  | Blackside Darter |  |  |  |
| Bluegill | Bluegill | Bluegill | Bluegill | Bluegill | Bluegill | Bluegill | Bluegill |
| Bluntnose Minnow | Bluntnose Minnow | Bluntnose Minnow | Bluntnose Minnow | Bluntnose Minnow | Bluntnose Minnow | Bluntnose Minnow | Bluntnose Minnow |
|  |  |  |  |  | Bowfin | Bowfin |  |
| Bridle Shiner |  |  |  |  | Bridle Shiner |  |  |
|  |  | Brindled Madtom |  |  |  |  | Brindled Madtom |
| Brook Silverside | Brook Silverside | Brook Silverside |  | Brook Silverside | Brook Silverside | Brook Silverside | Brook Silverside |
| Brook Stickleback | Brook Stickleback | Brook Stickleback |  | Brook Stickleback | Brook Stickleback | Brook Stickleback | Brook Stickleback |
| Brook Trout | Brook Trout | Brook Trout |  | Brook Trout |  | Brook Trout |  |
| Brown Bullhead | Brown Bullhead | Brown Bullhead | Brown Bullhead | Brown Bullhead | Brown Bullhead | Brown Bullhead | Brown Bullhead |
|  | Brown Trout | Brown Trout | Brown Trout | Brown Trout | Brown Trout | Brown Trout | Brown Trout |
| Butbot |  |  |  |  |  |  |  |
| Central Mudminnow | Central Mudminnow | Central Mudminnow |  | Central Mudminnow | Central Mudminnow | Central Mudminnow | Central Mudminnow |
| Central Stoneroller |  | Central Stoneroller | Central Stoneroller | Central Stoneroller | Central Stoneroller | Central Stoneroller | Central Stoneroller |
| Chain Pickerel |  | Chain Pickerel | Chain Pickerel |  | Chain Pickerel | Chain Pickerel | Chain Pickerel |
| Channel Catfish |  |  | Channel Catfish | Channel Catfish | Channel Catfish |  |  |
| Common Carp | Common Carp | Common Carp | Common Carp | Common Carp | Common Carp | Common Carp | Common Carp |
| Common Shiner | Common Shiner | Common Shiner | Common Shiner | Common Shiner | Common Shiner | Common Shiner | Common Shiner |
| Creek Chub | Creek Chub | Creek Chub | Creek Chub | Creek Chub | Creek Chub | Creek Chub | Creek Chub |
| Cutlip Minnow |  | Cutlip Minnow | Cutlip Minnow | Cutlip Minnow | Cutlip Minnow | Cutlip Minnow | Cutlip Minnow |
|  | Eastern Blacknose Dace | Eastern Blacknose Dace | Eastern Blacknose Dace | Eastern Blacknose Dace | Eastern Blacknose Dace | Eastern Blacknose Dace | Eastern Blacknose Dace |
| Eastern Creek Chubsucker |  |  | Eastern Creek Chubsucker |  |  |  |  |
|  |  | Eastern Silvery Minnow |  |  |  |  |  |
| Emerald Shiner |  |  |  | Emerald Shiner | Emerald Shiner |  | Emerald Shiner |
| Fallfish |  | Fallfish | Fallfish |  | Fallfish |  | Fallfish |
|  |  | Fantail Darter |  | Fantail Darter | Fantail Darter | Fantail Darter | Fantail Darter |
| Fathead Minnow |  | Fathead Minnow |  | Fathead Minnow | Fathead Minnow | Fathead Minnow | Fathead Minnow |
| Freshwater Drum | Freshwater Drum |  |  | Freshwater Drum | Freshwater Drum |  | Freshwater Drum |
|  |  |  |  | Gizzard Shad | Gizzard Shad |  | Gizzard Shad |
|  |  | Golden Redhorse |  | Golden Redhorse |  |  |  |
| Golden Shiner | Golden Shiner | Golden Shiner | Golden Shiner | Golden Shiner | Golden Shiner | Golden Shiner | Golden Shiner |
|  |  |  |  | Goldfish | Goldfish | Goldfish | Goldfish |
|  |  |  |  | Greater Redhorse |  |  |  |
|  |  |  |  |  |  |  | Green Sunfish |
| Greenside Darter | Greenside Darter | Greenside Darter |  | Greenside Darter | Greenside Darter |  | Greenside Darter |
| Hornyhead Chub | Hornyhead Chub | Hornyhead Chub |  | Hornyhead Chub | Hornyhead Chub |  | Hornyhead Chub |
| Iowa Darter | Iowa Darter |  |  |  | Iowa Darter |  | Iowa Darter |
|  |  |  |  |  |  |  | Johnny Darter |
|  |  |  |  |  |  |  | Lake Sturgeon |
|  |  |  |  |  | Lake Trout |  |  |
| Largemouth Bass | Largemouth Bass | Largemouth Bass | Largemouth Bass | Largemouth Bass | Largemouth Bass | Largemouth Bass | Largemouth Bass |
| Logperch | Logperch |  | Logperch | Logperch | Logperch |  | Logperch |
|  | Longnose Dace | Longnose Dace | Longnose Dace | Longnose Dace | Longnose Dace | Longnose Dace | Longnose Dace |
| Longnose Gar |  |  |  |  |  |  |  |
| Mimic Shiner | Mimic Shiner |  |  |  | Mimic Shiner |  | Mimic Shiner |
|  |  |  |  | Mottled Sculpin |  | Mottled Sculpin |  |
| Northern Hogsucker | Northern Hogsucker | Northern Hogsucker | Northern Hogsucker | Northern Hogsucker | Northern Hogsucker | Northern Hogsucker | Northern Hogsucker |
| Northern Pearl Dace |  |  |  |  |  |  |  |
| Northern Pike | Northern Pike | Northern Pike | Northern Pike | Northern Pike | Northern Pike | Northern Pike | Northern Pike |
| Northern Redelly Dace |  | Northern Redelly Dace |  |  |  |  | Northern Redelly Dace |
| Pumpkinseed | Pumpkinseed | Pumpkinseed | Pumpkinseed | Pumpkinseed | Pumpkinseed | Pumpkinseed | Pumpkinseed |
|  |  |  |  | Rainbow Trout | Rainbow Trout | Rainbow Trout |  |
| Rock Bass | Rock Bass | Rock Bass | Rock Bass | Rock Bass | Rock Bass | Rock Bass | Rock Bass |
| Rosyface Shiner |  | Rosyface Shiner |  | Rosyface Shiner |  |  | Rosyface Shiner |
|  |  |  |  |  |  |  | Round Goby |
|  |  |  |  | Sea Lamprey |  |  |  |
| Shorthead Redhorse | Shorthead Redhorse |  |  | Shorthead Redhorse | Shorthead Redhorse |  |  |
| Silver Redhorse |  |  | Silver Redhorse |  |  |  | Silver Redhorse |
|  |  |  | Slimy Sculpin | Slimy Sculpin |  | Slimy Sculpin |  |
| Smallmouth Bass | Smallmouth Bass | Smallmouth Bass | Smallmouth Bass | Smallmouth Bass | Smallmouth Bass |  | Smallmouth Bass |
|  | Spotfin Shiner | Spotfin Shiner |  | Spotfin Shiner | Spotfin Shiner |  | Spotfin Shiner |
|  |  | Spottail Shiner |  |  | Spottail Shiner |  | Spottail Shiner |
|  |  | Stonecat |  |  | Stonecat |  | Stonecat |
| Striped Shiner |  |  |  | Striped Shiner | Striped Shiner |  | Striped Shiner |
| Tadpole Madtom |  |  |  |  | Tadpole Madtom |  | Tadpole Madtom |
| Tessellated Darter | Tessellated Darter | Tessellated Darter |  | Tessellated Darter | Tessellated Darter | Tessellated Darter | Tessellated Darter |
|  |  |  |  |  |  | Tiger Musky |  |
|  |  |  |  |  |  |  | Trout-Perch |
| Walleye | Walleye |  | Walleye | Walleye | Walleye |  |  |
| White Bass | White Bass |  |  |  | White Bass |  |  |
|  |  |  | White Crappie |  | White Crappie | White Crappie | White Crappie |
|  |  |  |  | White Perch | White Perch |  | White Perch |
| White Sucker | White Sucker | White Sucker | White Sucker | White Sucker | White Sucker | White Sucker | White Sucker |
| Yellow Bullhead | Yellow Bullhead |  |  | Yellow Bullhead | Yellow Bullhead | Yellow Bullhead | Yellow Bullhead |
| Yellow Perch | Yellow Perch | Yellow Perch | Yellow Perch | Yellow Perch | Yellow Perch | Yellow Perch | Yellow Perch |

Skaneateles

| **1940** | **1950** | **1960** | **1970** | **1980** | **1990** | **2000** |
| --- | --- | --- | --- | --- | --- | --- |
|  |  |  | Alewife | Alewife | Alewife | Alewife |
|  |  |  | Atlantic Salmon | Atlantic Salmon | Atlantic Salmon | Atlantic Salmon |
|  |  |  | Bluegill | Bluegill | Bluegill | Bluegill |
| Bluntnose Minnow |  |  |  | Bluntnose Minnow | Bluntnose Minnow | Bluntnose Minnow |
|  |  |  | Brook Stickleback | Brook Stickleback | Brook Stickleback |  |
|  | Brook Trout | Brook Trout | Brook Trout | Brook Trout | Brook Trout |  |
| Brown Bullhead | Brown Bullhead | Brown Bullhead | Brown Bullhead | Brown Bullhead | Brown Bullhead | Brown Bullhead |
| Brown Trout | Brown Trout | Brown Trout | Brown Trout | Brown Trout | Brown Trout | Brown Trout |
| Central Stoneroller | Central Stoneroller | Central Stoneroller | Central Stoneroller |  | Central Stoneroller |  |
|  |  | Chain Pickerel | Chain Pickerel |  |  |  |
| Cisco |  | Cisco | Cisco | Cisco | Cisco | Cisco |
| Common Carp | Common Carp | Common Carp | Common Carp | Common Carp | Common Carp | Common Carp |
| Common Shiner | Common Shiner | Common Shiner | Common Shiner | Common Shiner | Common Shiner | Common Shiner |
| Creek Chub | Creek Chub | Creek Chub | Creek Chub | Creek Chub | Creek Chub | Creek Chub |
| Cutlip Minnow | Cutlip Minnow | Cutlip Minnow | Cutlip Minnow | Cutlip Minnow | Cutlip Minnow | Cutlip Minnow |
| Eastern Blacknose Dace | Eastern Blacknose Dace | Eastern Blacknose Dace | Eastern Blacknose Dace | Eastern Blacknose Dace | Eastern Blacknose Dace | Eastern Blacknose Dace |
| Eastern Silvery Minnow |  |  |  |  |  |  |
| Fallfish | Fallfish | Fallfish |  |  | Fallfish | Fallfish |
| Fantail Darter |  |  | Fantail Darter | Fantail Darter |  | Fantail Darter |
|  |  |  |  |  | Fathead Minnow | Fathead Minnow |
|  |  | Golden Shiner | Golden Shiner | Golden Shiner |  | Golden Shiner |
| Lake Trout |  | Lake Trout | Lake Trout | Lake Trout | Lake Trout | Lake Trout |
| Lake Whitefish |  | Lake Whitefish | Lake Whitefish | Lake Whitefish |  |  |
|  |  |  |  | Largemouth Bass | Largemouth Bass | Largemouth Bass |
|  |  |  |  |  |  | Logperch |
| Longnose Dace | Longnose Dace | Longnose Dace | Longnose Dace | Longnose Dace | Longnose Dace | Longnose Dace |
| Longnose Sucker |  | Longnose Sucker | Longnose Sucker | Longnose Sucker | Longnose Sucker | Longnose Sucker |
|  |  |  | Margined Madtom |  |  |  |
|  |  |  |  | Mottled Scuplin |  |  |
|  |  | Northern Hogsucker | Northern Hogsucker |  | Northern Hogsucker | Northern Hogsucker |
|  |  | Northern Pike | Northern Pike | Northern Pike | Northern Pike | Northern Pike |
| Pumpkinseed |  | Pumpkinseed | Pumpkinseed | Pumpkinseed | Pumpkinseed | Pumpkinseed |
| Rainbow Smelt |  | Rainbow Smelt | Rainbow Smelt | Rainbow Smelt | Rainbow Smelt | Rainbow Smelt |
| Rainbow Trout | Rainbow Trout | Rainbow Trout | Rainbow Trout | Rainbow Trout | Rainbow Trout | Rainbow Trout |
| Rock Bass |  | Rock Bass | Rock Bass | Rock Bass | Rock Bass | Rock Bass |
|  |  |  |  |  |  | Rudd |
| Satinfin Shiner |  |  |  |  |  |  |
|  |  | Slimy Sculpin |  | Slimy Sculpin |  |  |
| Smallmouth Bass | Smallmouth Bass | Smallmouth Bass | Smallmouth Bass | Smallmouth Bass | Smallmouth Bass | Smallmouth Bass |
| Spotfin Shiner |  |  |  |  |  |  |
| Spottail Shiner |  | Spottail Shiner | Spottail Shiner | Spottail Shiner | Spottail Shiner |  |
|  |  | Stonecat | Stonecat |  | Stonecat |  |
| Tessellated Darter |  |  |  | Tessellated Darter |  | Tessellated Darter |
|  |  |  |  |  |  | Tiger Musky |
| Walleye |  | Walleye | Walleye |  | Walleye | Walleye |
| White Sucker | White Sucker | White Sucker | White Sucker | White Sucker | White Sucker | White Sucker |
| Yellow Perch |  | Yellow Perch | Yellow Perch | Yellow Perch | Yellow Perch | Yellow Perch |

Syracuse

| **1930** | **1940** | **1950** | **1960** | **1970** | **1980** | **1990** | **2000** | **2010** |
| --- | --- | --- | --- | --- | --- | --- | --- | --- |
| Alewife | Alewife |  | Alewife | Alewife | Alewife | Alewife | Alewife | Alewife |
| American Eel |  | American Eel | American Eel | American Eel | American Eel | American Eel |  |  |
|  |  |  |  |  | Atlantic Salmon | Atlantic Salmon |  |  |
| Banded Killifish | Banded Killifish |  | Banded Killifish |  |  | Banded Killifish | Banded Killifish | Banded Killifish |
| Black Bullhead |  |  |  |  |  |  |  |  |
| Black Crappie | Black Crappie | Black Crappie | Black Crappie | Black Crappie | Black Crappie | Black Crappie | Black Crappie | Black Crappie |
|  |  |  |  |  |  |  |  | Blackside Darter |
| Blue Pike |  |  |  |  |  |  |  |  |
|  |  |  |  |  |  | Blueback Herring |  |  |
| Bluegill |  | Bluegill | Bluegill | Bluegill | Bluegill | Bluegill | Bluegill | Bluegill |
| Bluntnose Minnow | Bluntnose Minnow | Bluntnose Minnow | Bluntnose Minnow | Bluntnose Minnow | Bluntnose Minnow | Bluntnose Minnow | Bluntnose Minnow | Bluntnose Minnow |
|  |  | Bowfin | Bowfin | Bowfin | Bowfin | Bowfin | Bowfin | Bowfin |
|  |  |  |  |  | Brassy Minnow |  |  |  |
| Bridle Shiner |  |  |  |  |  |  |  |  |
|  |  |  |  |  |  |  |  | Brindled Madtom |
|  |  | Brook Silverside | Brook Silverside | Brook Silverside | Brook Silverside | Brook Silverside | Brook Silverside | Brook Silverside |
| Brook Stickleback |  | Brook Stickleback | Brook Stickleback | Brook Stickleback | Brook Stickleback | Brook Stickleback | Brook Stickleback | Brook Stickleback |
|  |  | Brook Trout | Brook Trout | Brook Trout | Brook Trout | Brook Trout | Brook Trout | Brook Trout |
| Brown Bullhead | Brown Bullhead | Brown Bullhead | Brown Bullhead | Brown Bullhead | Brown Bullhead | Brown Bullhead | Brown Bullhead | Brown Bullhead |
| Brown Trout |  | Brown Trout | Brown Trout | Brown Trout | Brown Trout | Brown Trout | Brown Trout |  |
|  |  |  |  | Burbot |  | Burbot |  |  |
| Central Mudminnow | Central Mudminnow | Central Mudminnow | Central Mudminnow | Central Mudminnow | Central Mudminnow | Central Mudminnow | Central Mudminnow | Central Mudminnow |
|  |  | Central Stoneroller | Central Stoneroller | Central Stoneroller |  | Central Stoneroller | Central Stoneroller | Central Stoneroller |
| Chain Pickerel |  | Chain Pickerel | Chain Pickerel | Chain Pickerel | Chain Pickerel | Chain Pickerel | Chain Pickerel | Chain Pickerel |
|  | Channel Catfish | Channel Catfish |  | Channel Catfish | Channel Catfish | Channel Catfish | Channel Catfish | Channel Catfish |
|  |  |  |  |  |  |  |  | Chinook Salmon |
|  |  | Cisco | Cisco | Cisco | Cisco |  |  |  |
|  |  |  |  |  |  |  |  | Coho Salmon |
| Common Carp | Common Carp | Common Carp | Common Carp | Common Carp | Common Carp | Common Carp | Common Carp | Common Carp |
| Common Shiner |  | Common Shiner | Common Shiner | Common Shiner | Common Shiner | Common Shiner | Common Shiner | Common Shiner |
|  | Creek Chub | Creek Chub | Creek Chub | Creek Chub | Creek Chub | Creek Chub | Creek Chub | Creek Chub |
| Cutlip Minnow | Cutlip Minnow | Cutlip Minnow | Cutlip Minnow | Cutlip Minnow | Cutlip Minnow | Cutlip Minnow | Cutlip Minnow | Cutlip Minnow |
| Eastern Blacknose Dace |  | Eastern Blacknose Dace | Eastern Blacknose Dace | Eastern Blacknose Dace | Eastern Blacknose Dace | Eastern Blacknose Dace | Eastern Blacknose Dace | Eastern Blacknose Dace |
| Eastern Creek Chubsucker | | Eastern Creek Chubsucker | Eastern Creek Chubsucker | Eastern Creek Chubsucker |  | Eastern Creek Chubsucker | Eastern Creek Chubsucker | Eastern Creek Chubsucker |
| Emerald Shiner | Emerald Shiner |  | Emerald Shiner | Emerald Shiner | Emerald Shiner | Emerald Shiner | Emerald Shiner |  |
| Fallfish | Fallfish | Fallfish | Fallfish | Fallfish | Fallfish | Fallfish | Fallfish | Fallfish |
|  |  | Fantail Darter |  | Fantail Darter | Fantail Darter | Fantail Darter | Fantail Darter | Fantail Darter |
|  |  |  | Fathead Minnow | Fathead Minnow | Fathead Minnow | Fathead Minnow | Fathead Minnow | Fathead Minnow |
|  |  | Freshwater Drum | Freshwater Drum | Freshwater Drum | Freshwater Drum | Freshwater Drum | Freshwater Drum |  |
|  |  | Gizzard Shad | Gizzard Shad | Gizzard Shad | Gizzard Shad | Gizzard Shad | Gizzard Shad | Gizzard Shad |
| Golden Shiner | Golden Shiner | Golden Shiner | Golden Shiner | Golden Shiner | Golden Shiner | Golden Shiner | Golden Shiner | Golden Shiner |
|  |  |  | Goldfish | Goldfish |  |  | Goldfish |  |
| Grass Pickerel |  | Grass Pickerel |  | Grass Pickerel |  | Grass Pickerel | Grass Pickerel | Grass Pickerel |
|  |  |  |  |  |  | Greater Redhorse |  |  |
|  |  |  |  |  |  | Green Sunfish |  | Green Sunfish |
| Iowa Darter |  |  |  |  |  |  |  |  |
|  |  | Lake Sturgeon |  |  | Lake Sturgeon |  | Lake Sturgeon | Lake Sturgeon |
|  |  | Lake Trout |  |  |  |  |  |  |
| Largemouth Bass | Largemouth Bass | Largemouth Bass | Largemouth Bass | Largemouth Bass | Largemouth Bass | Largemouth Bass | Largemouth Bass | Largemouth Bass |
| Logperch | Logperch |  | Logperch | Logperch | Logperch | Logperch | Logperch | Logperch |
|  |  | Longnose Dace | Longnose Dace | Longnose Dace | Longnose Dace | Longnose Dace | Longnose Dace |  |
|  |  | Longnose Gar |  | Longnose Gar | Longnose Gar | Longnose Gar | Longnose Gar |  |
|  |  |  |  | Longnose Sucker |  |  |  |  |
|  |  |  | Margined Madtom |  |  | Margined Madtom |  | Margined Madtom |
| Mimic Shiner |  |  |  |  |  |  |  |  |
|  |  |  |  |  |  | Mottled Sculpin | Mottled Sculpin |  |
|  |  | Northern Hogsucker | Northern Hogsucker | Northern Hogsucker |  | Northern Hogsucker | Northern Hogsucker | Northern Hogsucker |
| Northern Pike | Northern Pike | Northern Pike | Northern Pike | Northern Pike | Northern Pike | Northern Pike | Northern Pike | Northern Pike |
| Northern Redbelly Dace | Northern Redbelly Dace |  |  |  |  |  |  |  |
| Pumpkinseed | Pumpkinseed | Pumpkinseed | Pumpkinseed | Pumpkinseed | Pumpkinseed | Pumpkinseed | Pumpkinseed | Pumpkinseed |
|  |  |  |  |  |  | Quillback |  |  |
|  |  |  |  |  |  | Rainbow Smelt |  |  |
|  |  |  | Rainbow Trout | Rainbow Trout | Rainbow Trout | Rainbow Trout | Rainbow Trout |  |
|  |  |  | Redside Dace |  |  |  |  |  |
| Rock Bass |  | Rock Bass | Rock Bass | Rock Bass | Rock Bass | Rock Bass | Rock Bass | Rock Bass |
| Rosyface Shiner |  |  |  |  |  |  |  |  |
|  |  |  |  |  |  |  |  | Round Goby |
|  |  |  |  |  |  | Rudd | Rudd |  |
|  |  | Satinfin Shiner | Satinfin Shiner |  |  | Satinfin Shiner |  |  |
|  |  |  | Sea Lamprey | Sea Lamprey | Sea Lamprey |  |  |  |
|  | Shorthead Redhorse | Shorthead Redhorse | Shorthead Redhorse | Shorthead Redhorse | Shorthead Redhorse | Shorthead Redhorse | Shorthead Redhorse |  |
|  | Silver Redhorse |  | Silver Redhorse |  |  | Silver Redhorse | Silver Redhorse |  |
|  |  |  | Slimy Sculpin | Slimy Sculpin | Slimy Sculpin |  | Slimy Sculpin |  |
|  |  | Smallmouth Bass | Smallmouth Bass | Smallmouth Bass | Smallmouth Bass | Smallmouth Bass | Smallmouth Bass | Smallmouth Bass |
|  |  |  | Spotfin Shiner | Spotfin Shiner | Spotfin Shiner | Spotfin Shiner | Spotfin Shiner |  |
| Spottail Shiner |  | Spottail Shiner | Spottail Shiner | Spottail Shiner | Spottail Shiner | Spottail Shiner | Spottail Shiner | Spottail Shiner |
|  |  |  |  | Stonecat |  |  |  |  |
| Tadpole Madtom | Tadpole Madtom |  |  | Tadpole Madtom |  |  | Tadpole Madtom | Tadpole Madtom |
|  |  |  | Tessellated Darter | Tessellated Darter | Tessellated Darter | Tessellated Darter | Tessellated Darter | Tessellated Darter |
| Threespine Stickleback |  |  |  |  |  |  |  |  |
|  |  |  |  | Tiger Musky | Tiger Musky | Tiger Musky | Tiger Musky | Tiger Musky |
|  |  |  |  | Trout-Perch |  | Trout-Perch |  |  |
| Walleye | Walleye | Walleye | Walleye | Walleye | Walleye | Walleye | Walleye | Walleye |
|  | White Bass |  |  | White Bass | White Bass | White Bass |  |  |
|  |  |  |  | White Crappie | White Crappie | White Crappie | White Crappie | White Crappie |
|  |  | White Perch | White Perch | White Perch | White Perch | White Perch | White Perch | White Perch |
| White Sucker | White Sucker | White Sucker | White Sucker | White Sucker | White Sucker | White Sucker | White Sucker | White Sucker |
| Yellow Bullhead |  |  |  |  | Yellow Bullhead | Yellow Bullhead | Yellow Bullhead | Yellow Bullhead |
| Yellow Perch | Yellow Perch | Yellow Perch | Yellow Perch | Yellow Perch | Yellow Perch | Yellow Perch | Yellow Perch | Yellow Perch |
