# Supplementary material for: Land cover as a driver of fish community changes in New York’s Oswego River Watershed
Source: PLoS One. 2025 Jul 14;20(7):e0327293. doi: 10.1371/journal.pone.0327293 (PMC12258583; doi:10.1371/journal.pone.0327293)
Supplement: S7 Table — Tables showing the fixed effect, standard error of the fixed effect, fixed effect intercept, standard error of the fixed effect intercept, and ANOVA p-value (α = 0.05) for the models for each land cover type for each species grouping. (DOCX) [file pone.0327293.s010.docx]

**S7 Table. Results of the linear mixed effects model for each grouping of species.** Tables showing the fixed effect, standard error of the fixed effect, fixed effect intercept, standard error of the fixed effect intercept, and ANOVA p-value (α=0.05) for the models for each land cover type for each species grouping.

Sediment-Tolerant

|  | **Fixed effect** | **Fixed effect standard error** | **Fixed effect intercept** | **Fixed effect intercept standard error** | **ANOVA p-value** |
| --- | --- | --- | --- | --- | --- |
| **Urban** | 1.15 | 0.29 | 16.37 | 1.91 | 0.00035 |
| **Agriculture** | -0.45 | 0.073 | 42.52 | 4.38 | 0.00071 |
| **Natural** | Singular Fit Error | -- | -- | -- | -- |

Sediment-Intolerant Species

|  | **Fixed effect** | **Fixed effect standard error** | **Fixed effect intercept** | **Fixed effect intercept standard error** | **ANOVA p-value** |
| --- | --- | --- | --- | --- | --- |
| **Urban** | 0.20 | 0.13 | 7.076 | 0.78 | 0.14 |
| **Agriculture** | -0.071 | 0.035 | 11.31 | 1.78 | 0.076 |
| **Natural** | 0.084 | 0.040 | 4.41 | 1.79 | 0.068 |

High-Temperature-Tolerant Species

|  | **Fixed effect** | **Fixed effect standard error** | **Fixed effect intercept** | **Fixed effect intercept standard error** | **ANOVA p-value** |
| --- | --- | --- | --- | --- | --- |
| **Urban** | 1.15 | 0.27 | 14.20 | 1.81 | 0.00025 |
| **Agriculture** | -0.32 | 0.075 | 34.17 | 4.21 | 0.0031 |
| **Natural** | 0.34 | 0.10 | 4.85 | 4.83 | 0.030 |

High-Temperature-Intolerant Species

|  | **Fixed effect** | **Fixed effect standard error** | **Fixed effect intercept** | **Fixed effect intercept standard error** | **ANOVA p-value** |
| --- | --- | --- | --- | --- | --- |
| **Urban** | 0.36 | 0.25 | 18.77 | 1.75 | 0.16 |
| **Agriculture** | -0.17 | 0.075 | 28.56 | 3.84 | 0.040 |
| **Natural** | 0.18 | 0.082 | 12.63 | 3.67 | 0.047 |

Native Species

|  | **Fixed effect** | **Fixed effect standard error** | **Fixed effect intercept** | **Fixed effect intercept standard error** | **ANOVA p-value** |
| --- | --- | --- | --- | --- | --- |
| **Urban** | 1.11 | 0.43 | 27.64 | 2.81 | 0.014 |
| **Agriculture** | -0.39 | 0.12 | 50.64 | 6.27 | 0.010 |
| **Natural** | 0.49 | 0.14 | 11.91 | 6.54 | 0.016 |

Nonnative Species

|  | **Fixed effect** | **Fixed effect standard error** | **Fixed effect intercept** | **Fixed effect intercept standard error** | **ANOVA p-value** |
| --- | --- | --- | --- | --- | --- |
| **Urban** | 0.37 | 0.12 | 5.49 | 0.85 | 0.0046 |
| **Agriculture** | -0.20 | 0.040 | 16.58 | 2.18 | 0.036 |
| **Natural** | 0.047 | 0.047 | 5.023 | 2.11 | 0.44 |
