# Supplementary material for: Land cover as a driver of fish community changes in New York’s Oswego River Watershed
Source: PLoS One. 2025 Jul 14;20(7):e0327293. doi: 10.1371/journal.pone.0327293 (PMC12258583; doi:10.1371/journal.pone.0327293)
Supplement: S8 Table — Comparing linear mixed effect model results for full land cover and buffer land cover. (DOCX) [file pone.0327293.s011.docx]

**S8 Table. Full and Buffer Models.** Comparing linear mixed effect model results for full land cover and buffer land cover.

| **Species Grouping** | **Land Cover** | **Fixed Effect**  **(Full Land Cover)** | **p-value**  **(Full Land Cover)** | **Fixed Effect (Buffer Land Cover)** | **p-value (Buffer Land Cover)** |
| --- | --- | --- | --- | --- | --- |
| Full Dataset | Urban | 1.51 | **0.0056** | 1.96 | **0.013** |
| Full Dataset | Agriculture | -0.58 | **0.0071** | -0.50 | **0.0055** |
| Full Dataset | Natural | 0.74 | **0.022** | 0.57 | **0.029** |
| Sediment-Tolerant | Urban | 1.15 | **<0.001** | 1.58 | **<0.001** |
| Sediment-Tolerant | Agriculture | -0.45 | **<0.001** | -0.40 | **<0.001** |
| Sediment-Tolerant | Natural | Error | Error | Error | Error |
| Sediment-Intolerant | Urban | 0.20 | 0.14 | 0.30 | 0.10 |
| Sediment-Intolerant | Agriculture | -0.071 | 0.076 | -0.074 | 0.053 |
| Sediment-Intolerant | Natural | 0.084 | 0.068 | 0.088 | **0.050** |
| Temperature-Tolerant | Urban | 1.15 | **<0.001** | 1.44 | **<0.001** |
| Temperature-Tolerant | Agriculture | -0.32 | **0.0031** | Error | Error |
| Temperature-Tolerant | Natural | 0.34 | **0.030** | Error | Error |
| Temperature-Intolerant | Urban | 0.36 | 0.16 | 0.44 | 0.26 |
| Temperature-Intolerant | Agriculture | -0.17 | **0.040** | -0.16 | **0.044** |
| Temperature-Intolerant | Natural | 0.18 | **0.047** | 0.14 | 0.082 |
| Native | Urban | 1.11 | **0.014** | 1.35 | **0.030** |
| Native | Agriculture | -0.39 | **0.010** | -0.35 | **0.0078** |
| Native | Natural | 0.49 | **0.016** | 0.45 | **0.013** |
| Nonnative | Urban | 0.37 | **0.0046** | 0.57 | **0.0043** |
| Nonnative | Agriculture | -0.20 | **0.036** | -0.13 | **0.035** |
| Nonnative | Natural | 0.047 | 0.44 | 0.031 | 0.55 |
